# *O*‑Thien-2-yl Esters: A Synthetic Approach to a Rare Class of Materials Using a Modified Steglich Esterification

**DOI:** 10.1021/acs.joc.5c02523

**Published:** 2026-01-20

**Authors:** Anthony V. Bertolino, Megan A. Sroka, Emily P. Richards, Alexander J. Seed

**Affiliations:** Department of Chemistry and Biochemistry, 4229 Kent State University, Kent, Ohio 44242-0001, United States

## Abstract

Herein, we describe a simple procedure for the transformation of 3-thien-2-one to *O*-thien-2-yl esters via modified Steglich esterification. Attempted esterification using standard Steglich conditions gave low yields due to a competing O–N acyl migration, resulting in the formation of *N*-acylurea as the major product. The addition of a catalytic amount of *p*-TSA·H_2_O eliminates the formation of the *N*-acylurea. Various *O*-thien-2-yl esters have been synthesized using this procedure in yields of 51–85%.

## Graphical abstract

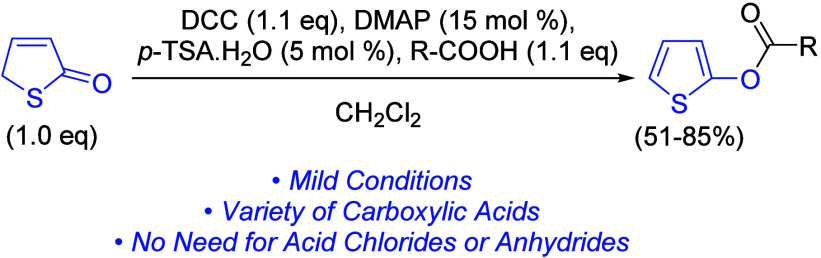

Thiophene-2-carboxylate esters have been utilized in the synthesis of a variety of mesogenic systems including ferroelectric, nematic, and other derivatives.^1–3^ These materials have displayed advanced properties when compared to analogous benzoate esters including increased birefringence, polarizability, and electrooptic response, for example.^4,5^ The incorporation of thiophene-2-carboxylate esters in materials for applications in organic solar cells and photovoltaics has also been reported.^6^ All of these known materials have the ester carbonyl group directly attached to the thiophene ring due primarily to the ease of synthesis and predicted mesomorphic behavior. Recently, our group has been interested in the preparation of analogous reversed ester systems, where the ethereal oxygen of the ester group is directly attached to the thiophene ring. Surprisingly, there is almost a dearth of methodology that may be used to access *O-*thien-2-yl esters. While traditional esterification methods for phenols work with ease, similar methods using thienols do not work.^7^ Unlike phenols, the vast majority of 2-thienols exist almost entirely as tautomeric mixtures of thienones (Scheme 1),^7,8^ with the exception of thienols that possess an intramolecular hydrogen bond.^9^

**Scheme 1:**
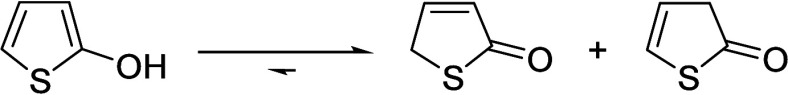
Tautomerization of Thienol to Thienones

The first reported transformation of thienones to *O-*thien-2-yl esters was conducted by Hurd and Kreuz.^10^ They reacted thien-2-one with aqueous sodium hydroxide and benzoyl chloride to form 2-thienyl benzoate (65%). Similarly, they also synthesized 2-thienyl acetate using thien-2-one in the presence of aqueous sodium hydroxide and acetic anhydride (56%). Analogous methodology was also reported by Ford and MacKay.^11^ Subsequently, Lee et al. reacted thien-2-one with four different acid chlorides in triethylamine to make the *O-*thien-2-yl esters.^12^ Tsuchimoto et al. reported the reaction of thien-2-one with an excess of acetic anhydride in the presence of triethylamine to give the *O*-thien-2-yl ester in a good yield (79%).^13^ Other reports^14,15^ describe an indirect route whereby thien-2-one is reacted with triphosgene to give di-2-thienyl carbonate, which is subsequently reacted with a carboxylic acid to provide *O-*thien-2-yl ester derivatives (<50 mg scale).

These very limited studies demonstrate the clear need for methodological development toward *O*-thienyl-2-ester targets. The great electrophilicity of acid chlorides and anhydrides and their moisture sensitivity make the current methodology somewhat unattractive and limited in scope. Additionally, the acid chloride functionality is incompatible with the other nucleophilic groups contained within the same substrate. Thus, we sought to find a mild one-pot method for the synthesis of these esters that would allow for broad functional group tolerance.

We postulated that *O-*thien-2-yl esters might be synthesized via a one-pot method based on previous work by our group. We were interested in how thien-2-ones could be converted to 2-alkoxythiophenes, and the products subsequently incorporated into the construction of thieno­[3,2-*b*]­thiophenes and thieno­[2,3-*b*]­thiophenes. We developed a single-step etherification protocol that utilized a Mitsunobu reaction between various thienones and octan-1-ol.^16^ The p*K*_a_ of 3-thien-2-one is reported to be 10.63.^17^ As far as we know, deprotonation of the thienone is crucial for the formation of the thienoxide (A, Scheme 2), which is a key intermediate for the formation of alkoxythiophenes via the Mitsunobu reaction.

**Scheme 2:**
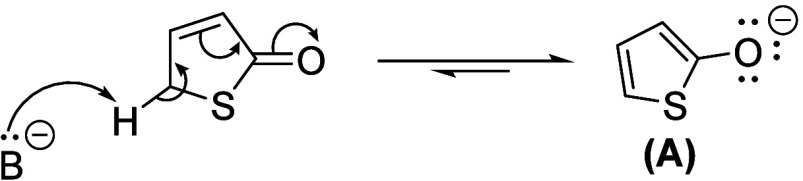
Deprotonation of 3-Thien-2-one to Form the Thienoxide (A)

We hypothesized that a similar deprotonation might allow for the formation of *O*-thienyl-2-esters using Steglich esterification. This would allow for an operationally very simple procedure that additionally eliminates the need for acid chlorides and anhydrides, allowing for a direct one-step coupling of 3-thien-2-one with carboxylic acids. This would be a particularly attractive approach given the broad functional group tolerance of the Steglich esterification.

Our initial attempt for this transformation was carried out in THF using DCC, *p*-anisic acid, DMAP, and 3-thien-2-one to form the *O-*thien-2-yl benzoate ester **1** (Scheme 3). We found that following chromatographic purification, **1** was isolated in only 37% yield. Analysis of the crude ^1^H NMR spectrum revealed the formation of an *N*-acylurea byproduct (approximately 1.5:1.0, *N*-acylurea:**1** by ^1^H NMR analysis), which was responsible for the low yield of **1** due to the consumption of the carboxylate (see Supporting Information for more details).

**Scheme 3:**
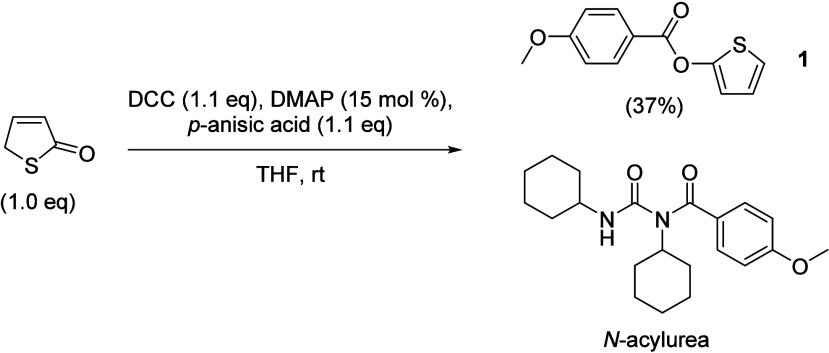
Initial Synthesis of **1** Using THF as the Solvent

*N*-Acylureas have been identified as byproducts in the Steglich esterification through an O–N acyl migration of the *O*-acylisourea intermediate (Scheme 4).^18,19^ It was noted by Sheehan et al. that solvents including tetrahydrofuran (THF) and dioxane are more likely to lead to the formation of the *N*-acylurea byproduct.^20^ In contrast, dichloromethane was shown to limit its formation.

**Scheme 4:**
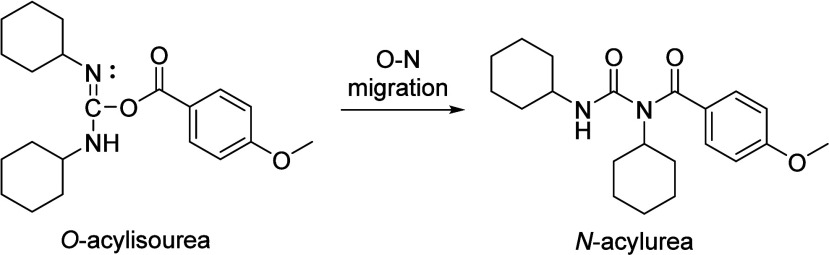
*N*-Acylurea Formation from the *O*-Acylisourea Intermediate

Upon changing the solvent to dichloromethane, we were pleased to see an increase in the formation of **1** and a decrease in the formation of *N*-acylurea (3.3:1.0, **1**:*N*-acylurea by ^1^H NMR analysis). After chromatographic purification, the isolated yield increased from 37 to 63%. However, still unsatisfied with the amount of *N*-acylurea byproduct, we sought additional means to limit its formation

Holmberg et al. reported that O–N acyl migration could be limited by adding a catalytic amount of *p*-toluenesulfonic acid (*p*-TSA) to the reaction.^18^ We were delighted to observe that the addition of a catalytic amount of *p*-TSA·H_2_O (∼5 mol %) eliminates the *N*-acylurea byproduct (^1^H NMR analysis). After column chromatography, **1** was isolated in an 85% yield (Table 1).

**Table 1:**
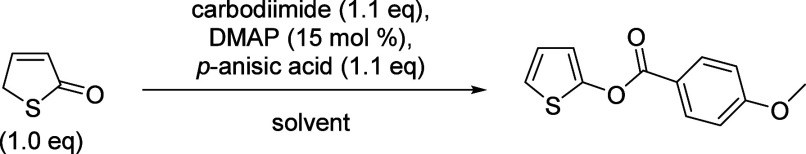
Optimization of the Synthetic Procedure

entry	solvent	*p*-TSA·H_2_O (mol %)	carbodiimide	time	temp	yield
1	THF		DCC	o/n	rt	37%
2	CH_2_CI_2_		DCC	o/n	rt	63%
3^a^	CH_2_CI_2_	5%	DCC	o/n	rt	85%
4	CH_2_CI_2_	5%	DIC	o/n	rt	63%
5	CH_2_CI_2_	5%	EDC	o/n	rt	75%
6	MeCN	5%	DCC	o/n	rt	75%
7	toluene	5%	DCC	o/n	rt	75%

a Optimized conditions.

Following this discovery, we subsequently evaluated acetonitrile and toluene as solvents for this reaction. Slightly lower yields of 75% were recorded in both cases (see Table 1 and **1c** and **1d** in the Supporting Information). Different carbodiimides were also evaluated as alternatives to DCC. The use of *N*,*N′*-diisopropylcarbodiimide (DIC) gave a 63% yield (see **1a** in the Supporting Information). Similarly, the use of *N*-(3-dimethylaminopropyl)-*N*-ethylcarbodiimide hydrochloride (EDC) gave a 75% yield (see **1b** in the Supporting Information).

A postulated mechanism for the effect of the addition of *p*-TSA·H_2_O is presented in Scheme 5. We propose that the addition of *p*-TSA·H_2_O will protonate the basic nitrogen atom of the *O*-acylisourea, which will result in the formation of a strong intramolecular hydrogen bond, thus inhibiting the acyl migration. Additionally, this will further enhance the electrophilicity of the carbonyl carbon, accelerating nucleophilic attack by DMAP. Once the activated DMAP-ester is formed, the thienoxide can readily react, thus forming **1.**

**Scheme 5:**
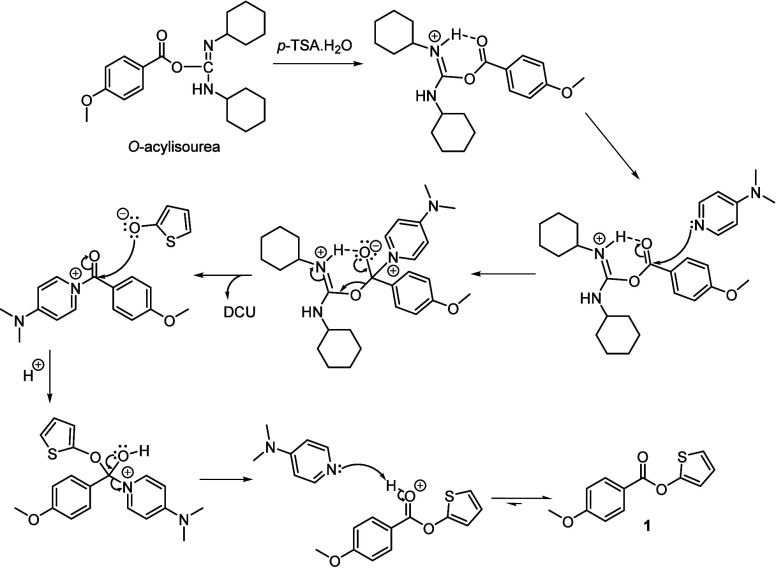
Proposed Mechanism of the Steglich Esterification Using Catalytic Amounts of *p*-TSA·H_2_O

Once we optimized the reaction conditions, we turned our attention to the scope of the reaction. We selected a variety of aryl, heteroaryl, and alkyl carboxylic acids with both electron-donating and electron-withdrawing groups (including sterically hindered systems) to react with 3-thien-2-one (Table 2). Aryl carboxylic acids containing electron-donating groups gave good yields from 72 to 85%. Aryl carboxylic acids with electron-withdrawing groups gave slightly lower yields ranging from 63 to 82% (the stronger the electron-withdrawing group, the lower the yield; this is consistent with a reduction in nucleophilicity of the carboxylate). The sterically hindered ester **8** was synthesized in a moderate 55% yield. We also wanted to ensure that the reaction would work with alkyl carboxylic acids, and we found that γ-keto ester **10** and butanoate ester **11** could be synthesized in 61 and 82% yields, respectively.

**Table 2:**
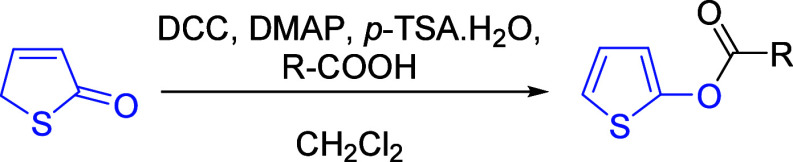

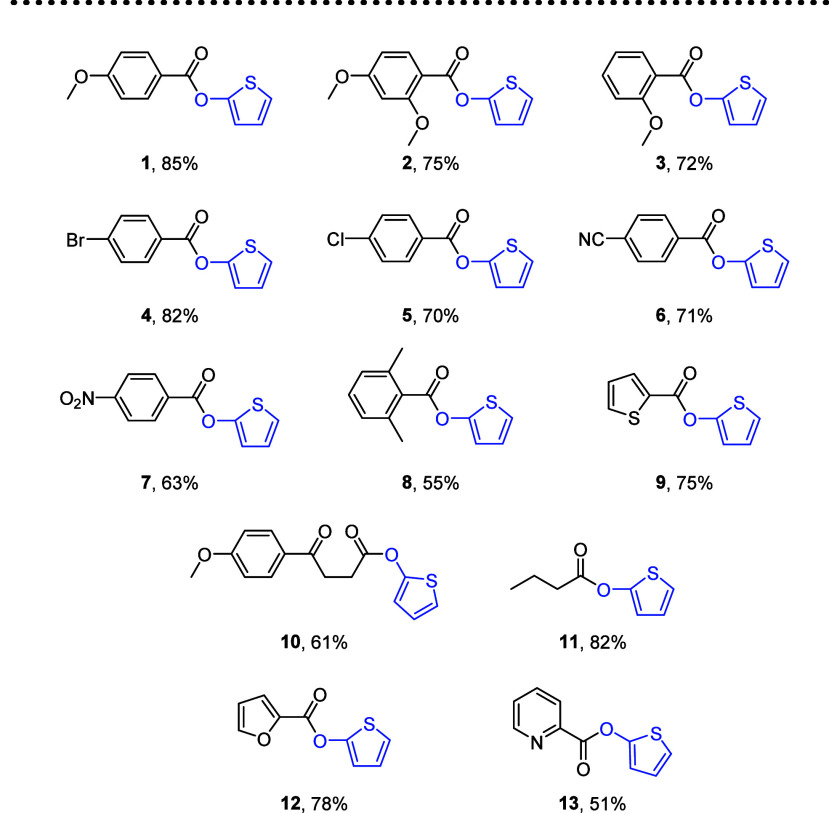
Substrate Scope of the Carboxylic Acids^a^

a Reaction conditions: 3-thien-2-one (1.0 equiv), DCC (1.1 equiv), carboxylic acid (1.1 equiv), DMAP (15 mol %), *p*-TSA·H_2_O (5 mol %), CH_2_Cl_2_, room temperature, overnight.

It should be noted that 3-thien-2-one is stable when stored under an argon atmosphere in the freezer (orange solid). After 1 week of storage, the physical properties and ^1^H NMR spectrum remained unchanged. We presume the storage of this material is indefinite; however, we have not used the material after a 2-week period. If the 3-thien-2-one is left out in the open air at room temperature, then it will become a black tar within 24–48 h. Therefore, it is imperative that this material is kept under an argon atmosphere and in the freezer for a prolonged life.

In summary, we have presented an experimentally simple one-pot synthesis of *O*-thienyl-esters in good yields. Our method eliminates the need for using acid chlorides and anhydrides and has a broad functional group tolerance. This reaction works with a broad scope of various carboxylic acids (aryl-EDG, aryl-EWG, heteroaryl, alkyl, and sterically hindered). The incorporation of *O-*thienyl-esters into advanced materials for electrooptic applications is also underway in our laboratory.

## Supplementary Material



## Data Availability

The data underlying this study are available in the published article and its online Supporting Information.

## Notes

Detailed experimental procedures, ^1^H and ^13^C {^1^H} NMR spectra, and HRMS data (PDF)
