# Supplementary material for: O‑Thien-2-yl Esters: A Synthetic Approach to a Rare Class of Materials Using a Modified Steglich Esterification
Source: J Org Chem. 2026 Jan 20;91(4):1856–9. doi: 10.1021/acs.joc.5c02523 (PMC12865768; doi:10.1021/acs.joc.5c02523)
Supplement: Supplementary file 1 [file jo5c02523_si_001.pdf]

Supporting Information for:

O-Thien-2-yl esters: A synthetic approach to a rare class of materials using a modified Steglich esterification

Anthony V. Bertolino, Megan A. Sroka, Emily P. Richards, and Alexander J. Seed*

Department of Chemistry and Biochemistry, Kent State University, Kent, Ohio 44242-0001, USA

Table of Contents:

I. *Materials and Methods*……………………………………………………………*S2*

II. *Synthesis of Compounds **1-13***…………………………………………..……..……..*S3*

III. *References*…..………………………………………………………….……….*S15*

IV. *NMR Spectra*…………………...…………………………………………*S16-S111*

Materials and Methods

Reaction flasks were oven dried prior to use. Anhydrous tetrahydrofuran (THF), dichloromethane (CH_2Cl_2) and toluene were dried using a solvent purification system by passing the degassed solvents through activated alumina columns (built by Pure Process Technology, LLC using a J-KEM Digital Vacuum Regulator, model 280). Anhydrous acetonitrile (MeCN) was purchased from Sigma Aldrich. Solvents used for workups and purification met ACS reagent grade specifications. Petroleum ether was redistilled before use. 3-Thien-2-one [1] and 3-(4-methoxybenzoyl)propanoic acid [2] were prepared using previously described procedures. 2,4-Dimethoxybenzoic acid, and thiophene-2-carboxylic acid were purchased from Ambeed. 2,6-Dimethylbenzoic acid and furan-2-carboxylic acid were purchased from Oakwood Chemical. All other reagents were purchased from Sigma Aldrich and TCI. Thin layer chromatography (TLC) was carried out using Silicycle brand aluminum backed plates (200μm thick layer of 60Å silica gel with UV 254nm and 365nm fluorescence indicator). Column chromatography (flash) was carried out using Silicycle brand 60Å, 40-63μm particle size silica. Melting points were determined by polarizing optical microscopy using a Laborlux 12 POLS polarizing microscope combined with a Mettler FP82HT hot stage and a Mettler FP90 central processor. Confirmation of the structures of products was obtained by ^{1}H (500MHz) and ^{13}C {^{1}H} (126MHz) NMR (Agilent 500 MHz, MestReNova v 14.2.2-28739) spectroscopy in $CDCl_3$ (The spectra for compound **7** were recorded in DMSO-d_6 solvent) with tetramethylsilane as an internal standard. High-resolution mass spectra (HRMS) were recorded using a Thermo Scientific Exactive Plus Orbitrap Mass Spectrometer.

(1) *O*-Thien-2-yl 4-methoxybenzoate

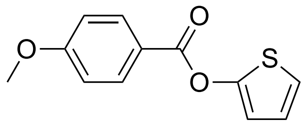

Dichloromethane (35 mL, anhydrous) was added to an argon purged flask containing *p*-anisic acid (1.1330 g, 7.4466 mmol), *N*,*N'*-dicyclohexylcarbodiimide (DCC) (1.5380 g, 7.4541 mmol), 4-(*N*,*N*-dimethylamino)pyridine (DMAP) (122.6 mg, 1.004 mmol) and *p*-toluenesulfonic acid monohydrate (*p*-TSA.H_2O) (64.3 mg, 0.338 mmol) before being stirred at room temperature for 10 minutes. 3-Thien-2-one (0.6773 g, 6.764 mmol) in dichloromethane (6 mL) was added dropwise over a five-minute period. By the end of the addition the reaction mixture had turned dark yellow in color with a visible white suspension. The reaction mixture was allowed to stir at room temperature overnight (TLC analysis revealed a complete reaction). The reaction mixture was then filtered, and the filtrate was concentrated *in vacuo* to afford a brown residue. The residue was dry loaded onto silica gel and purified via flash column chromatography (5% ethyl acetate in petroleum ether, silica gel) to afford **1** as a white solid. Yield = 1.3437 g (85%). Mp = 62.2-63.2 °C. ^{1}H NMR (500 MHz, $CDCl_3$) δ 8.14 (d, *J* = 8.9 Hz, 2H), 6.99 (d, *J* = 8.9 Hz, 2H), 6.93 (dd, *J* = 5.8, 1.6 Hz, 1H), 6.88 (dd, *J* = 5.8, 3.8 Hz, 1H), 6.82 (dd, *J* = 3.8, 1.6 Hz, 1H), 3.90 (s, 3H). ^{13}C {^{1}H} NMR (126 MHz, $CDCl_3$) δ 164.2, 163.1, 152.3, 132.4, 123.3, 120.6, 118.1, 114.0, 113.3, 55.6. HRMS (ESI) m/z: $[M+Na]^+$ calculated for $[C_{12}H_{10}NaO_3S]^+$ 257.0243, found 257.0241.

(1a) *Synthesis of O-Thien-2-yl 4-methoxybenzoate **1** using N,N'*-diisopropylcarbodiimide *(DIC) as the carbodiimide:*

Dichloromethane (35 mL, anhydrous) was added to an argon purged flask containing *p*-anisic acid (0.3345 g, 2.198 mmol), DIC (0.35 mL, *d* = 0.81 g/mL at 20 °C, 2.2 mmol), DMAP (39.5 mg, 0.323 mmol) and *p*-TSA.H_2O (19.0 mg, 0.0999 mmol) before being stirred at room temperature for 10 minutes. 3-Thien-2-one (0.2090 g, 2.087 mmol) in dichloromethane (6 mL) was added dropwise over a five-minute period. By the end of the addition the homogeneous reaction mixture turned light pink in color. The reaction mixture was allowed to stir at room temperature overnight (TLC analysis revealed a complete reaction). The reaction mixture was then concentrated *in vacuo* to afford a brown residue. The residue was dry loaded onto silica gel and purified via flash column chromatography (5% ethyl acetate in petroleum ether, silica gel) to afford **1** as a white solid. Yield = 0.3067 g (63%).

(1b) *Synthesis of O-Thien-2-yl 4-methoxybenzoate **1** using N*-(3-dimethylaminopropyl)-*N*-ethylcarbodiimide hydrochloride *(EDC) as the carbodiimide:*

Dichloromethane (35 mL, anhydrous) was added to an argon purged flask containing *p*-anisic acid (0.3395 g, 2.231 mmol), EDC (0.4352 g, 2.270 mmol), DMAP (38.4 mg, 0.314 mmol) and *p*-TSA.H_2O (21.6 mg, 0.114 mmol) before being stirred at room temperature for 10 minutes. 3-Thien-2-one (0.2004 g, 2.001 mmol) in dichloromethane (6 mL) was added dropwise over a five-minute period. By the end of the addition the homogeneous reaction mixture turned pale-yellow in color. The reaction mixture was allowed to stir at room temperature overnight (TLC analysis revealed a complete reaction). The reaction mixture was then concentrated *in vacuo* to afford a

brown residue. The residue was dry loaded onto silica gel and purified via flash column chromatography (5% ethyl acetate in petroleum ether, silica gel) to afford **1** as a white solid. Yield = 0.3527 g (75%).

(1c) *Synthesis of O-Thien-2-yl 4-methoxybenzoate **1** using DCC as the carbodiimide and acetonitrile as the solvent:*

Acetonitrile (25 mL, anhydrous) was added to an argon purged flask containing *p*-anisic acid (0.1679 g, 1.104 mmol), DCC (0.2294 g, 1.112 mmol), DMAP (18.2 mg, 0.149 mmol) and *p*-TSA.H_2O (10.7 mg, 0.0563 mmol) before being stirred at room temperature for 10 minutes. 3-Thien-2-one (99.6 mg, 0.995 mmol) in acetonitrile (3 mL) was added dropwise over a five-minute period. By the end of the addition the reaction had turned light pink with a visible white precipitate. The reaction was allowed to stir at room temperature overnight (TLC analysis revealed a complete reaction). The reaction mixture was then filtered, and the filtrate was concentrated *in vacuo* to afford a dark brown residue. The residue was dry loaded onto silica gel and purified via flash column chromatography (5% ethyl acetate in petroleum ether, silica gel) to afford **1** as a white solid. Yield = 0.1758 g (75%).

(1d) *Synthesis of O-Thien-2-yl 4-methoxybenzoate using DCC as the carbodiimide and toluene as the solvent:*

Toluene (25 mL, anhydrous) was added to an argon purged flask containing *p*-anisic acid (0.1727 g, 1.135 mmol), DCC (0.2376 g, 1.152 mmol), DMAP (19.2 mg, 0.157 mmol) and *p*-TSA.H_2O (11.0 mg, 0.0578 mmol) before being stirred at room temperature for 10 minutes. 3-Thien-2-one

(0.1032 g, 1.031 mmol) in toluene (3 mL) was added dropwise over a five-minute period. By the end of the addition the reaction had turned pale yellow with a visible white precipitate. The reaction mixture was allowed to stir at room temperature overnight (TLC analysis revealed a complete reaction). The reaction mixture was then filtered, and the filtrate was concentrated *in vacuo* to afford a dark brown residue. The residue was dry loaded onto silica gel and purified via flash column chromatography (5% ethyl acetate in petroleum ether, silica gel) to afford **1** as a white solid. Yield = 0.1811 g (75%).

(2) *O*-Thien-2-yl 2,4-dimethoxybenzoate

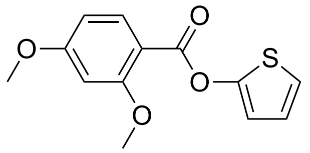

Compound **2** was prepared using a similar procedure to that described for the preparation of **1** using the quantities stated: 3-thien-2-one (0.3117 g, 3.113 mmol, in 6 mL of CH_2Cl_2), 2,4-dimethoxybenzoic acid (0.6245 g, 3.428 mmol), DCC (0.7046 g, 3.415 mmol), DMAP (57.3 mg, 0.469 mmol), *p*-TSA.H_2O (30.3 mg, 0.159 mmol), and dichloromethane (35 mL, anhydrous). The crude product was purified via flash column chromatography (15% ethyl acetate in petroleum ether, silica gel) to afford **2** as a tan solid. Yield = 0.6162 g (75%). Mp = 65.7-68.1 °C. ^{1}H NMR (500 MHz, $CDCl_3$) δ 8.06 (d, *J* = 8.7 Hz, 1H), 6.90 (dd, *J* = 5.8, 1.6 Hz, 1H), 6.86 (dd, *J* = 5.8, 3.8 Hz, 1H), 6.79 (dd, *J* = 3.9, 1.6 Hz, 1H), 6.56 (dd, *J* = 8.8, 2.3 Hz, 1H), 6.53 (d, *J* = 2.3 Hz, 1H), 3.94 (s, 3H), 3.89 (s, 3H). ^{13}C {^{1}H} NMR (126 MHz, $CDCl_3$) δ 165.3, 162.5, 161.4, 152.3, 134.7, 123.0, 117.9, 112.8, 109.9, 105.0, 99.0, 56.1, 55.6. HRMS (ESI) m/z: $[M+Na]^+$ calculated for $[C_{13}H_{12}NaO_4S]^+$ 287.0349, found 287.0347.

(3) *O*-Thien-2-yl 2-methoxybenzoate

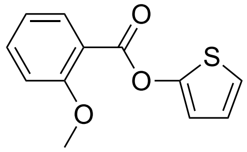

Compound **3** was prepared using a similar procedure to that described for the preparation of **1** using the quantities stated: 3-thien-2-one (0.4903 g, 4.896 mmol, in 6 mL of CH_2Cl_2), *o*-anisic acid (0.8196 g, 5.387 mmol), DCC (1.1135 g, 5.3967 mmol), DMAP (86.4 mg, 0.707 mmol), *p*-TSA.H_2O (47.2 mg, 0.248 mmol), and dichloromethane (35 mL, anhydrous). The crude product was purified via flash column chromatography (10% ethyl acetate in petroleum ether, silica gel) to afford **3** as an off-white solid. Yield = 0.8312 g (72%). Mp = 33.8-35.5 °C. ^{1}H NMR (500 MHz, $CDCl_3$) δ 8.02 (dd, *J* = 7.9, 1.6 Hz, 1H), 7.56 (dt, *J* = 8.2, 1.8 Hz, 1H), 7.05 (t, *J* = 7.8 Hz, 2H), 6.92 (dd, *J* = 5.7, 1.6 Hz, 1H), 6.87 (dd, *J* = 5.8, 3.8 Hz, 1H), 6.83 (dd, *J* = 3.8, 1.6 Hz, 1H), 3.96 (s, 3H). ^{13}C {^{1}H} NMR (126 MHz, $CDCl_3$) δ 162.0, 160.2, 152.2, 134.9, 132.4, 123.1, 120.3, 118.0, 117.7, 113.3, 112.3, 56.1. HRMS (ESI) m/z: $[M+Na]^+$ calculated for $[C_{12}H_{10}NaO_3S]^+$ 257.0243, found 257.0241.

(4) *O*-Thien-2-yl 4-bromobenzoate

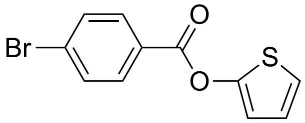

Compound **4** was prepared using a similar procedure to that described for the preparation of **1** using the quantities stated: 3-thien-2-one (0.3041 g, 3.037 mmol, in 6 mL of CH_2Cl_2), 4-bromobenzoic acid (0.6726 g, 3.346 mmol), DCC (0.6902 g, 3.345 mmol), DMAP (56.1 mg,

0.459 mmol), *p*-TSA.H_2O (29.2 mg, 0.154 mmol), and dichloromethane (35 mL, anhydrous). The crude product was purified via flash column chromatography (20% dichloromethane in petroleum ether, silica gel) to afford **4** as a white solid. Yield = 0.7011 g (82%). Mp = 90.6-92.9 °C. ^{1}H NMR (500 MHz, $CDCl_3$) δ 8.05 (d, *J* = 8.6 Hz, 2H), 7.67 (d, *J* = 8.6 Hz, 2H), 6.95 (dd, *J* = 5.8, 1.6 Hz, 1H), 6.89 (dd, *J* = 5.8, 3.8 Hz, 1H), 6.85 (dd, *J* = 3.9, 1.6 Hz, 1H). ^{13}C {^{1}H} NMR (126 MHz, $CDCl_3$) δ 162.7, 151.9, 132.1, 131.7, 129.3, 127.3, 123.4, 118.4, 113.7. HRMS (ESI) m/z: $[M+Na]^+$ calculated for $[C_{11}H_7BrNaO_2S]^+$ 304.9242, found 304.9240.

(5) *O*-Thien-2-yl 4-chlorobenzoate

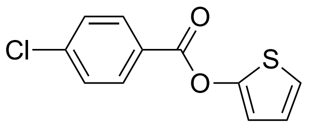

Compound **5** was prepared using a similar procedure to that described for the preparation of **1** using the quantities stated: 3-thien-2-one (0.3013 g, 3.009 mmol, in 6 mL of CH_2Cl_2), 4-chlorobenzoic acid (0.5190 g, 3.315 mmol), DCC (0.6827 g, 3.309 mmol), DMAP (55.3 mg, 0.453 mmol), *p*-TSA.H_2O (29.4 mg, 0.155 mmol), and dichloromethane (35 mL, anhydrous). The crude product was purified via flash column chromatography (15% dichloromethane in petroleum ether, silica gel) to afford **5** as a white solid. Yield = 0.5022 g (70%). Mp = 89.3-91.7 °C. ^{1}H NMR (500 MHz, $CDCl_3$) δ 8.13 (d, *J* = 8.5 Hz, 2H), 7.50 (d, *J* = 8.5 Hz, 2H), 6.95 (dd, *J* = 5.8, 1.6 Hz, 1H), 6.89 (dd, *J* = 5.7, 3.7 Hz, 1H), 6.85 (dd, *J* = 3.8, 1.6 Hz, 1H). ^{13}C {^{1}H} NMR (126 MHz, $CDCl_3$) δ 162.5, 151.9, 140.6, 131.6, 129.1, 126.9, 123.4, 118.4, 113.7. HRMS (ESI) m/z: $[M+Na]^+$ calculated for $[C_{11}H_7ClNaO_2S]^+$ 260.9747, found 260.9747.

(6) *O*-Thien-2-yl 4-cyanobenzoate

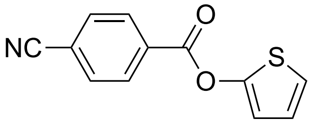

Compound **6** was prepared using a similar procedure to that described for the preparation of **1** using the quantities stated: 3-thien-2-one (0.1797 g, 1.794 mmol, in 6 mL of CH_2Cl_2), 4-cyanobenzoic acid (0.2918 g, 1.983 mmol), DCC (0.4072 g, 1.974 mmol), DMAP (33.8 mg, 0.277 mmol), *p*-TSA.H_2O (17.5 mg, 0.0920 mmol), and dichloromethane (35 mL, anhydrous). The crude product was purified via flash column chromatography (5% ethyl acetate in petroleum ether, silica gel) to afford **6** as a white solid. Yield = 0.2910 g (71%). Mp = 131.8-134.0 °C. ^{1}H NMR (500 MHz, $CDCl_3$) δ 8.30 (d, J = 8.4 Hz, 2H), 7.83 (d, J = 8.4 Hz, 2H), 6.98 (dd, J = 5.7, 1.7 Hz, 1H), 6.91 (dd, J = 5.7, 3.8 Hz, 1H), 6.89 (dd, J = 3.9, 1.7 Hz, 1H). ^{13}C {^{1}H} NMR (126 MHz, $CDCl_3$) δ 161.8, 151.6, 132.5, 132.3, 130.7, 123.5, 118.7, 117.7, 117.4, 114.0. HRMS (ESI) m/z: $[M+Na]^+$ calculated for $[C_{12}H_7NNaO_2S]^+$ 252.0090, found 252.0088.

(7) *O*-Thien-2-yl 4-nitrobenzoate

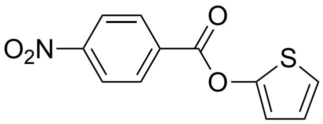

Compound **7** was prepared using a similar procedure to that described for the preparation of **1** using the quantities stated: 3-thien-2-one (0.1330 g, 1.328 mmol), 4-nitrobenzoic acid (0.2492 g, 1.491 mmol), DCC (0.3035 g, 1.471 mmol), DMAP (22.9 mg, 0.187 mmol), *p*-TSA.H_2O (14.2 mg, 0.0747 mmol), and dichloromethane (25 mL, anhydrous). The crude product was purified via

flash column chromatography (5% ethyl acetate in petroleum ether, silica gel) to afford **7** as a yellow solid. Yield = 0.2081 g (63%). Mp = 127.5-130.9 °C. ^{1}H NMR (500 MHz, DMSO-d_6) δ 8.41 (d, *J* = 9.0 Hz, 2H), 8.37 (d, *J* = 9.0 Hz, 2H), 7.25 (dd, *J* = 5.8, 1.7 Hz, 1H), 7.03 (dd, *J* = 3.8, 1.7 Hz, 1H), 6.97 (dd, *J* = 5.8, 3.9 Hz, 1H). ^{13}C {^{1}H} NMR (126 MHz, DMSO-d_6) δ 162.0, 151.4, 151.2, 133.8, 131.9, 124.6, 124.2, 120.0, 115.1. HRMS m/z (ESI) calculated for $[M+]^{\bullet-}$ 249.0101, found 249.0100. HRMS (ESI) m/z: $[M+]^{\bullet-}$ calculated for $[C_{11}H_7NO_4S]^{\bullet-}$ 249.0101, found 249.0100.

(8) *O*-Thien-2-yl 2,6-dimethylbenzoate

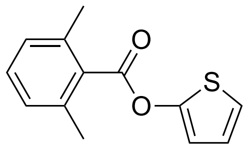

Compound **8** was prepared using a similar procedure to that described for the preparation of **1** using the quantities stated: 3-thien-2-one (0.1522 g, 1.520 mmol, in 6 mL of CH_2Cl_2), 2,6-dimethylbenzoic acid (0.2526 g, 1.682 mmol), DCC (0.3450 g, 1.672 mmol), DMAP (28.0 mg, 0.229 mmol), *p*-TSA.H_2O (14.9 mg, 0.0783 mmol), and dichloromethane (25 mL, anhydrous). The crude product was purified via flash column chromatography (30% dichloromethane in petroleum ether, silica gel) to afford **8** as a white solid. Yield = 0.1943 g (55%). Mp = 67.0-71.1 °C. ^{1}H NMR (500 MHz, $CDCl_3$) δ 7.26 (d, *J* = 7.8 Hz, 1H), 7.10 (d, *J* = 7.6 Hz, 2H), 6.96 (dd, *J* = 5.8, 1.6 Hz, 1H), 6.89 (dd, *J* = 5.8, 3.8 Hz, 1H), 6.81 (dd, *J* = 3.8, 1.6 Hz, 1H), 2.42 (s, 6H). ^{13}C {^{1}H} NMR (126 MHz, $CDCl_3$) δ 166.1, 151.8, 136.0, 131.7, 130.3, 127.9, 123.4, 118.2, 113.4, 20.1. HRMS (ESI) m/z: $[M+Na]^{+}$ calculated for $[C_{13}H_{12}NaO_2S]^{+}$ 255.0450, found 255.0448.

(9) *O*-Thien-2-yl thiophene-2-carboxylate

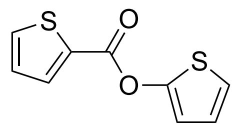

Compound **9** was prepared using a similar procedure to that described for the preparation of **1** using the quantities stated: 3-thien-2-one (93.6 mg, 0.935 mmol, in 3 mL of CH_2Cl_2), thiophene-2-carboxylic acid (0.1331 g, 1.039 mmol), DCC (0.2125 g, 1.030 mmol), DMAP (17.3 mg, 0.142 mmol), *p*-TSA.H_2O (9.1 mg, 0.0048 mmol), and dichloromethane (20 mL, anhydrous). The crude product was purified via flash column chromatography (2% ethyl acetate in petroleum ether, silica gel) to afford **9** as a white solid. Yield = 0.1473 g (75%). Mp = 53.1-55.4 °C (Lit = 54 °C [3]). ^{1}H NMR (500 MHz, $CDCl_3$) δ 7.99 (dd, *J* = 3.8, 1.3 Hz, 1H), 7.69 (dd, *J* = 5.0, 1.3 Hz, 1H), 7.19 (dd, *J* = 5.0, 3.8 Hz, 1H), 6.94 (dd, *J* = 5.8, 1.6 Hz, 1H), 6.88 (dd, *J* = 5.8, 3.8 Hz, 1H), 6.83 (dd, *J* = 3.8, 1.6 Hz, 1H). ^{13}C {^{1}H} NMR (126 MHz, $CDCl_3$) δ 158.9, 151.8, 135.2, 134.2, 131.5, 128.2, 123.4, 118.3, 113.7. HRMS (ESI) m/z: $[M+Na]^+$ calculated for $[C_9H_6NaO_2S_2]^+$ 232.9701, found 232.9701.

(10) *O*-Thien-2-yl 3-(4-methoxybenzoyl)propanoate

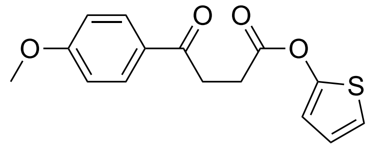

Compound **10** was prepared using a similar procedure to that described for the preparation of **1** using the quantities stated: 3-thien-2-one (61.5 mg, 0.614 mmol, in 3 mL of CH_2Cl_2), 3-(4-methoxybenzoyl)propanoic acid (0.1419 g, 0.6815 mmol), DCC (0.1433 g, 0.6945 mmol),

DMAP (11.0 mg, 0.0900 mmol), *p*-TSA.H_2O (6.3 mg, 0.033 mmol), and dichloromethane (25 mL, anhydrous). The crude product was purified via flash column chromatography (100% dichloromethane, silica gel) to afford **10** as a white solid. Yield = 0.1095 g (61%). Mp = 88.9-91.0 °C. ^{1}H NMR (500 MHz, $CDCl_3$) δ 7.97 (d, J = 8.9 Hz, 2H), 6.94 (d, J = 8.9 Hz, 2H), 6.86 (dd, J = 5.8, 1.6 Hz, 1H), 6.81 (dd, J = 5.8, 3.8 Hz, 1H), 6.71 (dd, J = 3.8, 1.6 Hz, 1H), 3.86 (s, 3H), 3.37 (t, J = 6.6 Hz, 2H), 2.99 (t, J = 6.6 Hz, 2H). ^{13}C {^{1}H} NMR (126 MHz, $CDCl_3$) δ 195.9, 169.9, 163.7, 151.9, 130.4, 129.4, 123.3, 117.9, 113.8, 113.3, 55.5, 32.8, 28.2. HRMS (ESI) m/z: $[M+Na]^+$ calculated for $[C_{15}H_{14}NaO_4S]^+$ 313.0505, found 313.0503.

(11) *O*-Thien-2-yl butanoate

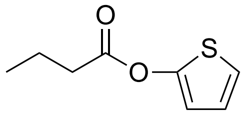

Compound **11** was prepared using a similar procedure to that described for the preparation of **1** using the quantities stated: 3-thien-2-one (0.4919 g, 4.912 mmol, in 6 mL of CH_2Cl_2), *n*-butyric acid (0.5 mL, d = 0.9574 g/mL at 20 °C, 5 mmol), DCC (1.1167 g, 5.4122 mmol), DMAP (89.6 mg, 0.733 mmol), *p*-TSA.H_2O (49.0 mg, 0.258 mmol), and dichloromethane (35 mL, anhydrous). The crude product was purified via flash column chromatography (25% dichloromethane in petroleum ether, silica gel) to afford **11** as a colorless oil. Yield = 0.6893 g (82%). ^{1}H NMR (500 MHz, $CDCl_3$) δ 6.88 (dd, J = 5.8, 1.5 Hz, 1H), 6.82 (dd, J = 5.8, 3.8 Hz, 1H), 6.69 (dd, J = 3.8, 1.5 Hz, 1H), 2.54 (t, J = 7.4 Hz, 2H), 1.78 (sext, J = 7.4 Hz, 2H), 1.03 (t, J = 7.4 Hz, 3H). ^{13}C {^{1}H} NMR (126 MHz, $CDCl_3$) δ 170.2, 152.0, 123.2, 117.9, 113.1, 35.8, 18.3, 13.6. HRMS (ESI) m/z: $[M+Na]^+$ calculated for $[C_8H_{10}NaO_2S]^+$ 193.0294, found 193.0295.

(12) *O*-Thien-2-yl furan-2-carboxylate

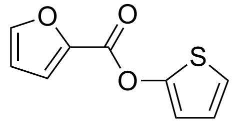

Compound **12** was prepared using a similar procedure to that described for the preparation of **1** using the quantities stated: 3-thien-2-one (106.5 mg, 1.064 mmol, in 3 mL of CH_2Cl_2), furan-2-carboxylic acid (0.1330 g, 1.187 mmol), DCC (0.2425 g, 1.175 mmol), DMAP (19.6 mg, 0.160 mmol), *p*-TSA.H_2O (11.0 mg, 0.0578 mmol), and dichloromethane (25 mL, anhydrous). The crude product was purified via flash column chromatography (10% ethyl acetate in petroleum ether, silica gel) to afford **12** as a white solid. Yield = 0.1605 g (78%). Mp = 61.4-64.6 °C (Lit = 56-59 °C [4]). ^{1}H NMR (500 MHz, $CDCl_3$) δ 7.69 (dd, J = 1.7, 0.9 Hz, 1H), 7.41 (dd, J = 3.5, 0.9 Hz, 1H), 6.94 (dd, J = 5.8, 1.6 Hz, 1H), 6.87 (dd, J = 5.8, 3.9 Hz, 1H), 6.84 (dd, J = 3.9, 1.6 Hz, 1H), 6.60 (dd, J = 3.6, 1.7 Hz, 1H). ^{13}C {^{1}H} NMR (126 MHz, $CDCl_3$) δ 155.1, 151.4, 147.7, 143.0, 123.5, 120.2, 118.4, 113.9, 112.4. HRMS (ESI) m/z: $[M+Na]^+$ calculated for $[C_9H_6NaO_3S]^+$ 216.9930, found 216.9931.

(13) *O*-Thien-2-yl pyridine-2-carboxylate

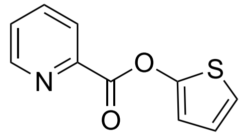

Compound **13** was prepared using a similar procedure to that described for the preparation of **1** using the quantities stated: 3-thien-2-one (137.3 mg, 1.371 mmol, in 3 mL of CH_2Cl_2), 2-picolinic acid (0.1851 g, 1.504 mmol), DCC (0.3123 g, 1.514 mmol), DMAP (24.8 mg, 0.203

mmol), *p*-TSA.H_2O (12.9 mg, 0.0678 mmol), and dichloromethane (25 mL, anhydrous). The crude product was purified via flash column chromatography (2% ethyl acetate in dichloromethane, silica gel) to afford **13** as a light pink solid. Yield = 0.1422 g (51%). Mp = 49.3-56.7 °C. ^{1}H NMR (500 MHz, $CDCl_3$) δ 8.86 (ddd, J = 4.7, 1.7, 0.9 Hz, 1H), 8.28 (dt, J = 7.9, 1.1 Hz, 1H), 7.93 (td, J = 7.7, 1.7 Hz, 1H), 7.57 (ddd, J = 7.7, 4.7, 1.2 Hz, 1H), 6.98 – 6.95 (m, 2H), 6.90 (dd, J = 5.7, 3.9 Hz, 1H). ^{13}C {^{1}H} NMR (126 MHz, $CDCl_3$) δ 162.0, 151.9, 150.3, 146.6, 137.3, 127.7, 126.1, 123.4, 118.5, 114.2. HRMS (ESI) m/z: $[M+H]^+$ calculated for $[C_{10}H_8NO_2S]^+$ 206.0270, found 206.0271.

References

(1) Tietz, J. I.; Seed, A. J.; Sampson, P. Preparation of Brominated 2-Alkoxythiophenes via Oxidation and Etherification of 2-Thienyltrifluoroborate Salts. *Org. Lett.* **2012**, *14* (19), 5058–5061. https://doi.org/10.1021/ol3022897.

(2) Sonpatki, V. M.; Herbert, M. R.; Sandvoss, L. M.; Seed, A. J. Troublesome Alkoxythiophenes. A Highly Efficient Synthesis via Cyclization of γ-Keto Esters. *J. Org. Chem.* **2001**, *66* (22), 7283–7286. https://doi.org/10.1021/jo015644j.

(3) Ford, M. C.; MacKay, D. 928. Decomposition Reactions of Heterocyclic Diacyl Peroxides. Part II. 2-Thenoyl Peroxide. *J. Chem. Soc.* **1957**, 4620. https://doi.org/10.1039/jr9570004620.

(4) Lee, C. K.; Yu, J. S.; Kim, S. H. Synthesis of Five-membered 2-heteroaryl 2-heteroaromatic Carboxylates and Attempted Cyclization to Bisheteroaryl[2,3- *b* :3′,2′- *d*]Pyran-2-one. *Journal of Heterocyclic Chem* **1998**, *35* (4), 835–841. https://doi.org/10.1002/jhet.5570350409.

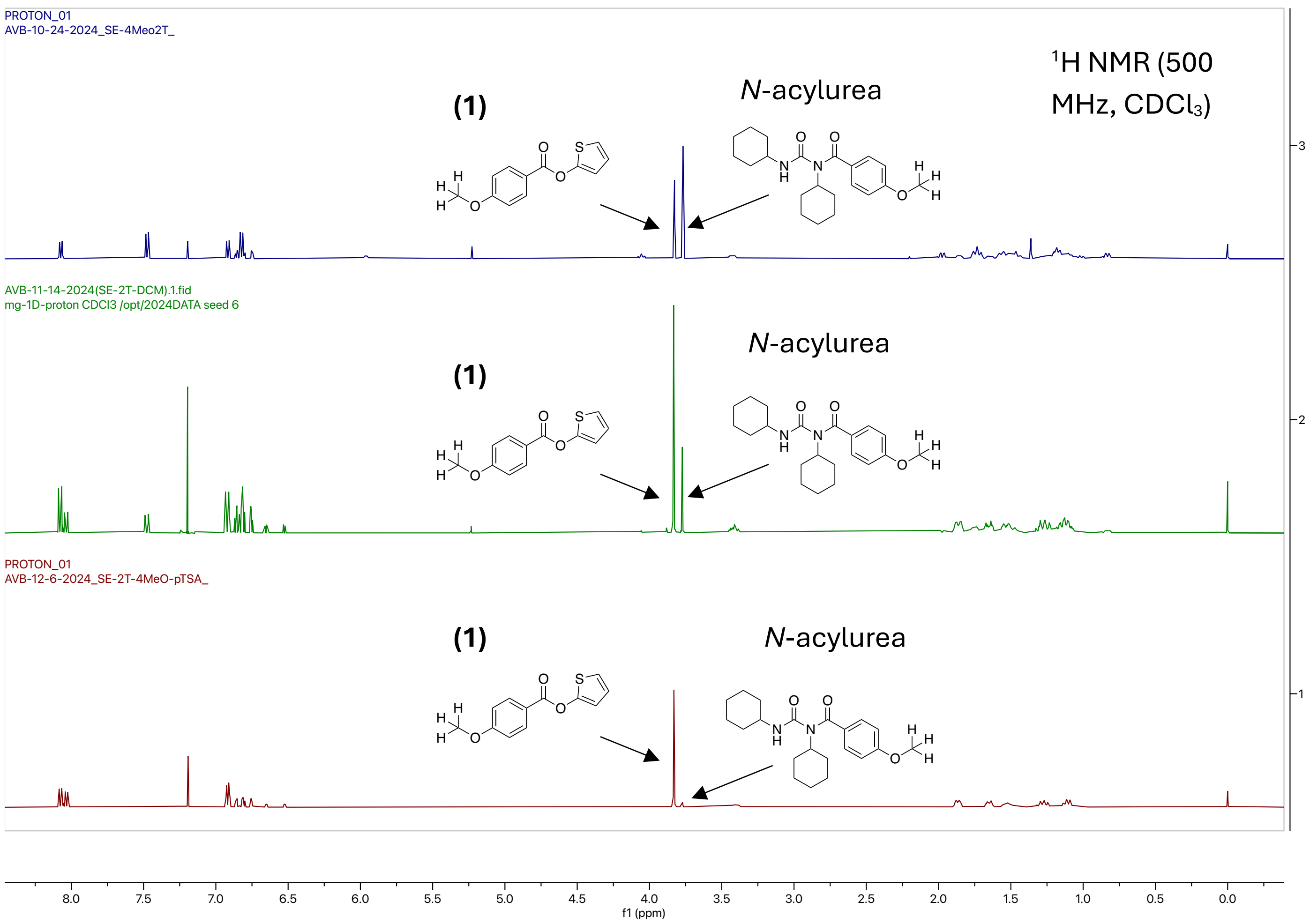

Crude ^{1}HNMR spectra of the Steglich esterification reaction using 3-thien-2-one and *p*-anisic acid. Reaction conducted in THF (top/blue). Reaction conducted in CH_2Cl_2 (middle/green). Reaction conducted in CH_2Cl_2 with 5 mol % of *p*-TSA.H_2O (bottom/red).

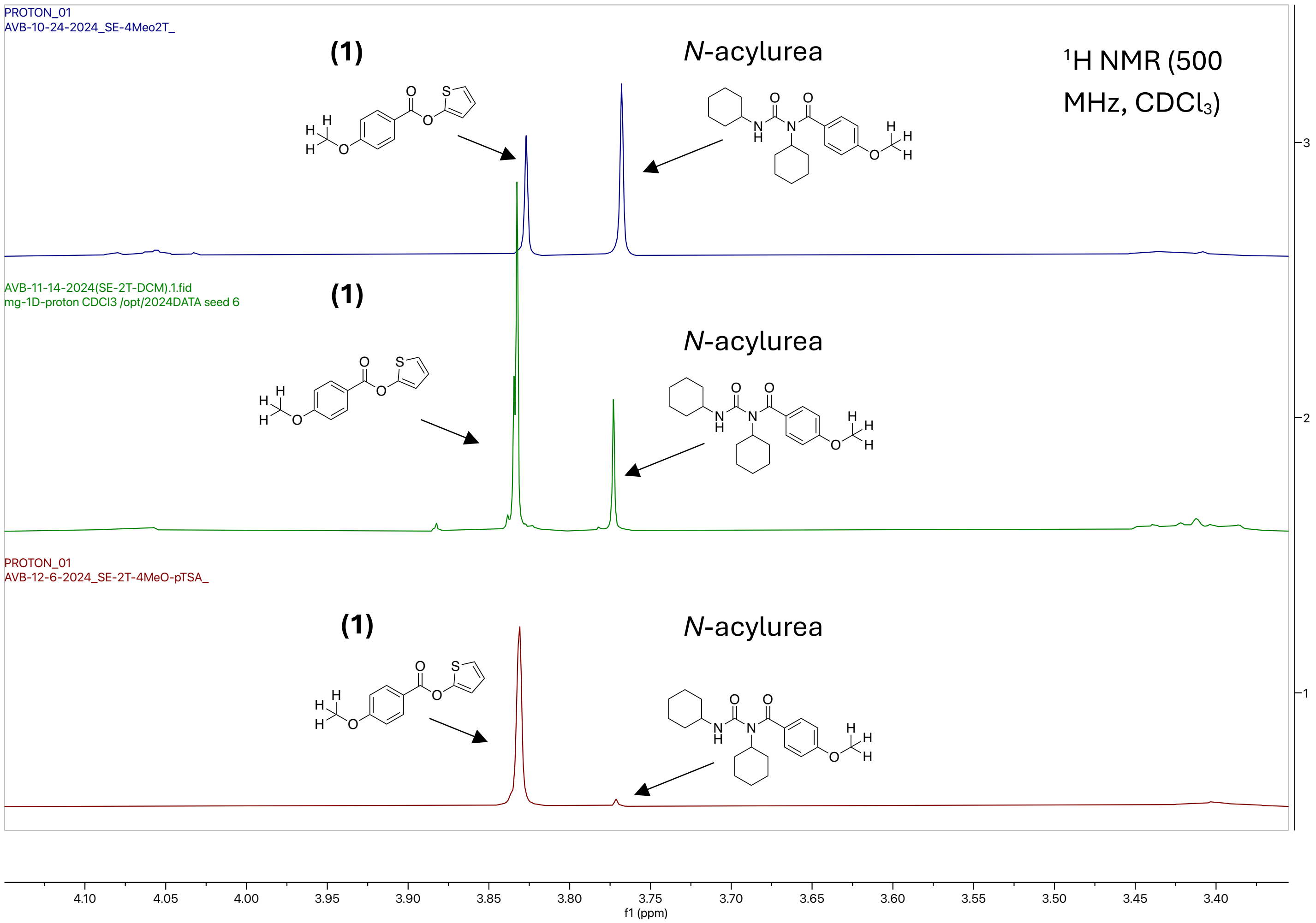

Expansion of the methoxy region in the crude ^{1}HNMR spectra.

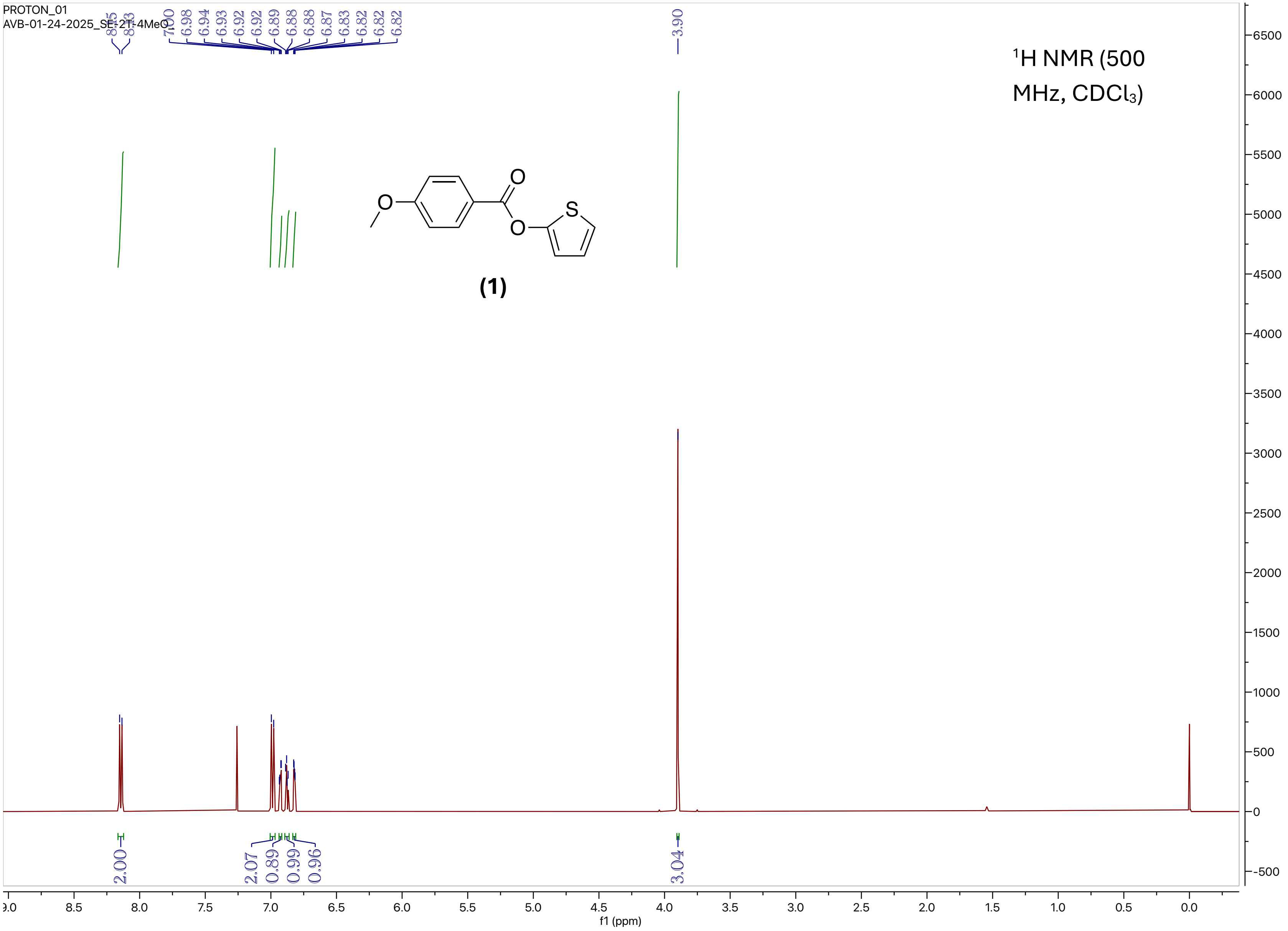
PROTON_01
AVB-01-24-2025_SE-2T-4MeO
8.15
8.13
7.00
6.98
6.94
6.93
6.92
6.92
6.89
6.88
6.88
6.87
6.83
6.82
6.82
6.82
3.90
¹H NMR (500 MHz, CDCl₃)
O
O
O
S
(1)
2.00
2.07
0.89
0.99
0.96
3.04
f1 (ppm)

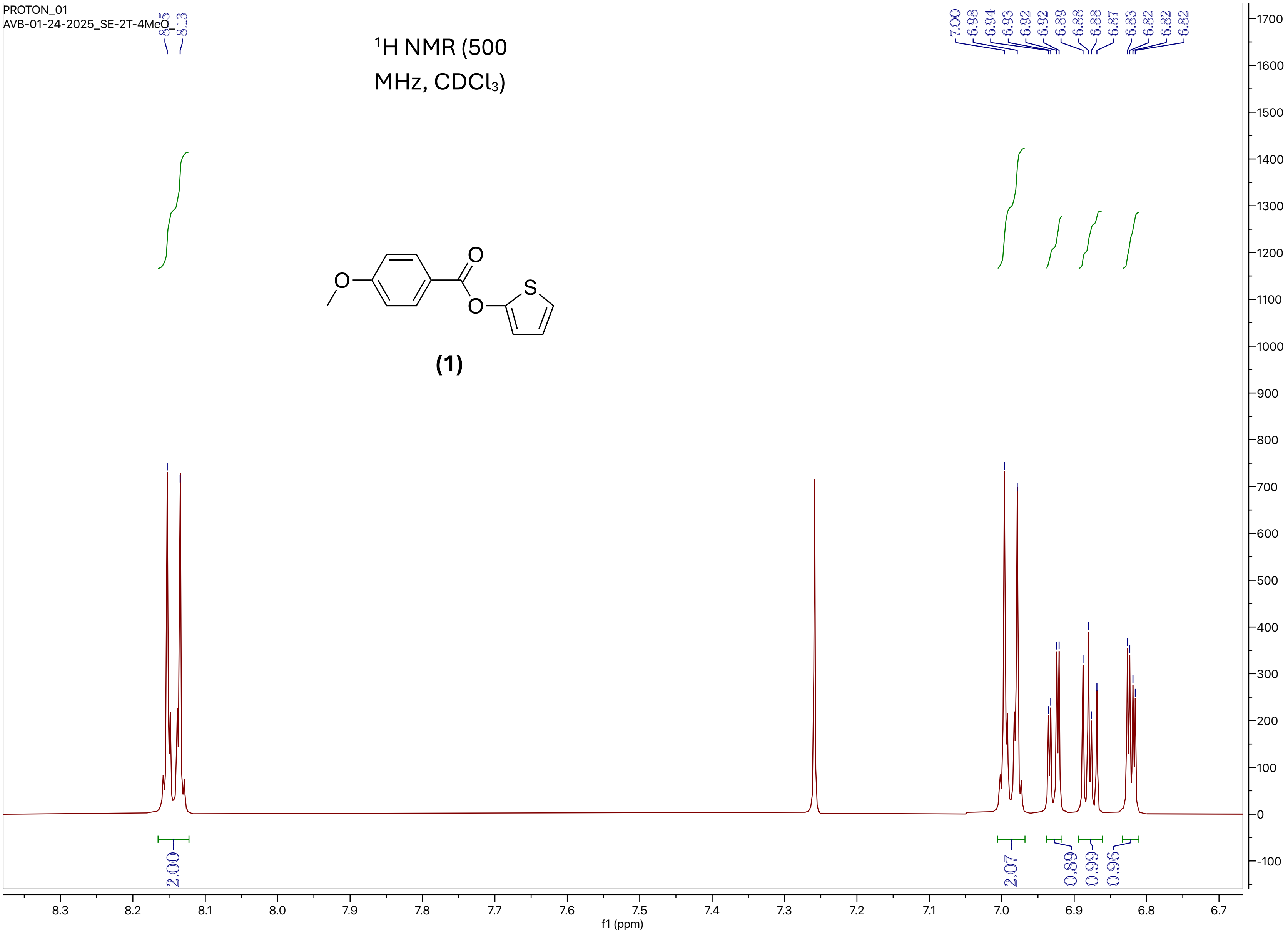
PROTON_01
AVB-01-24-2025_SE-2T-4MeO
8.15
8.13
7.00
6.98
6.94
6.93
6.92
6.92
6.89
6.88
6.88
6.87
6.83
6.82
6.82
6.82
^{1}H NMR (500 MHz, $CDCl_3$)
(1)
2.00
2.07
0.89
0.99
0.96
f1 (ppm)

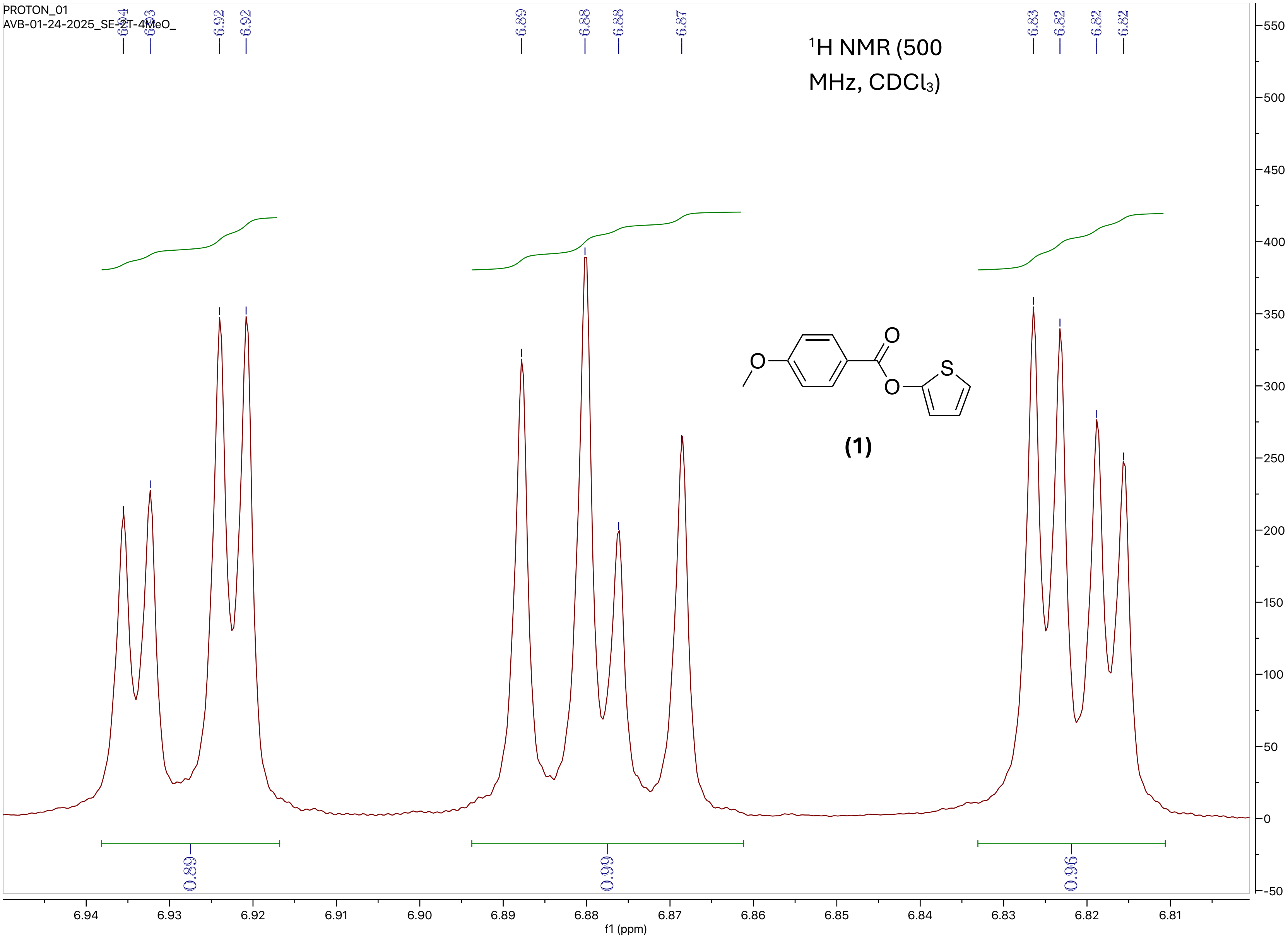
PROTON_01
AVB-01-24-2025_SE-2T-4MeO_
6.94
6.93
6.92
6.92
6.89
6.88
6.88
6.87
6.83
6.82
6.82
6.82
¹H NMR (500 MHz, CDCl₃)
(1)
0.89
0.99
0.96
f1 (ppm)

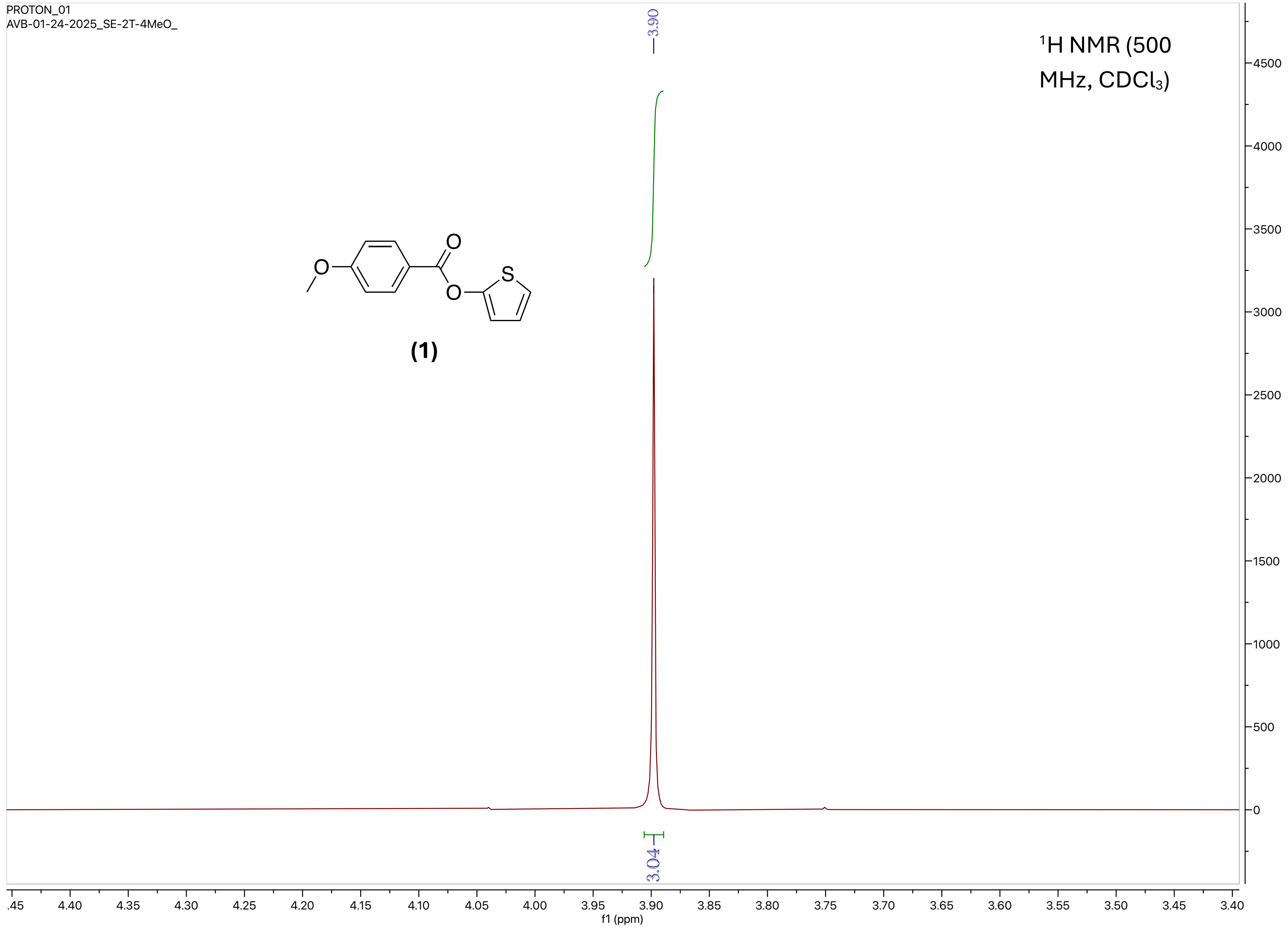
PROTON_01
AVB-01-24-2025_SE-2T-4MeO_
3.90
1H NMR (500 MHz, CDCl3)
O
O
O
S
(1)
3.04
f1 (ppm)

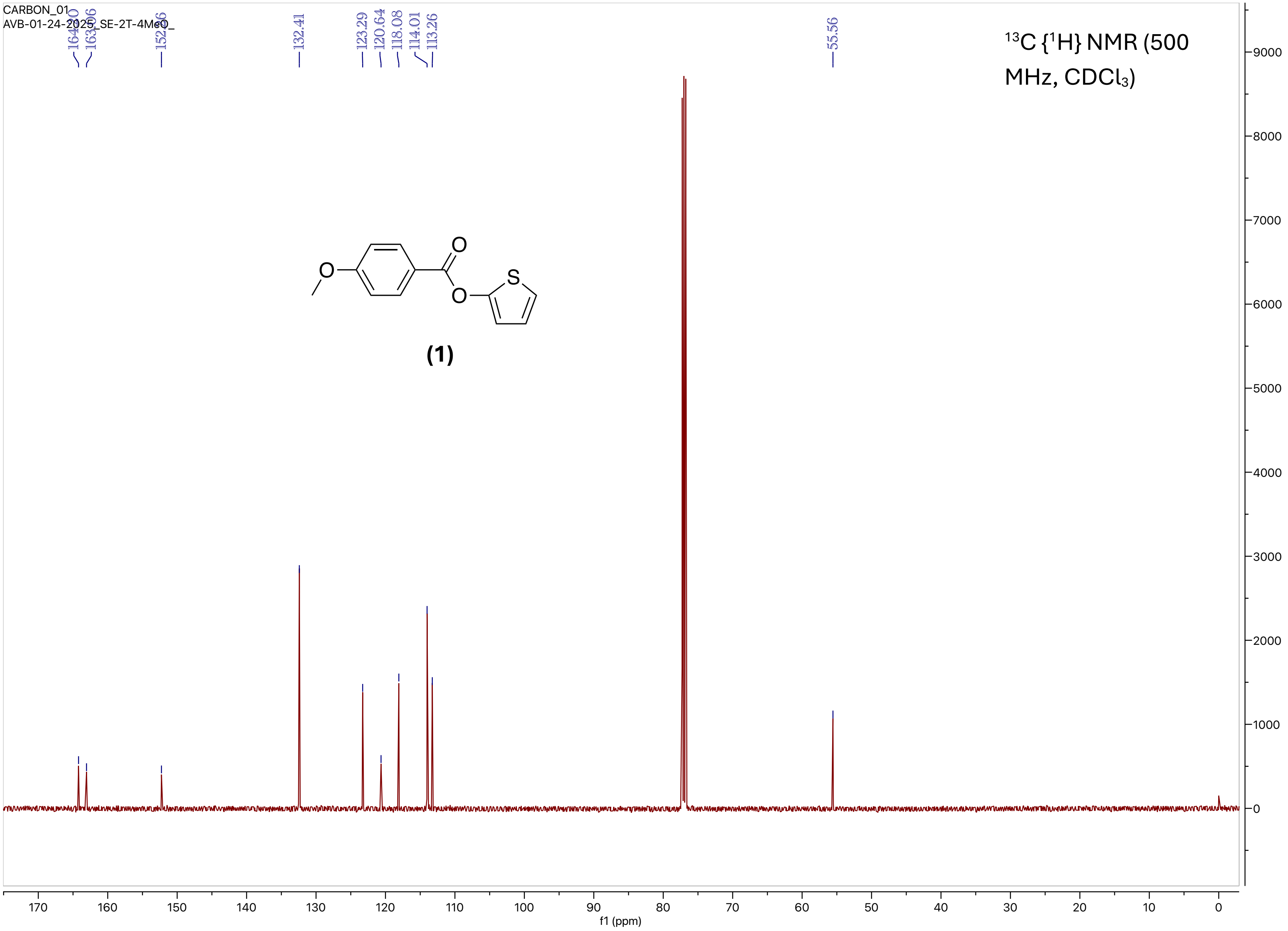

CARBON_01
AVB-01-24-2025_SE-2T-4MeO_
163.06
132.41
123.29
120.64
118.08
114.01
113.26
55.56
$^{13}C\{^1H\}$ NMR (500 MHz, $CDCl_3$)
O
O
O
S
(1)
f1 (ppm)

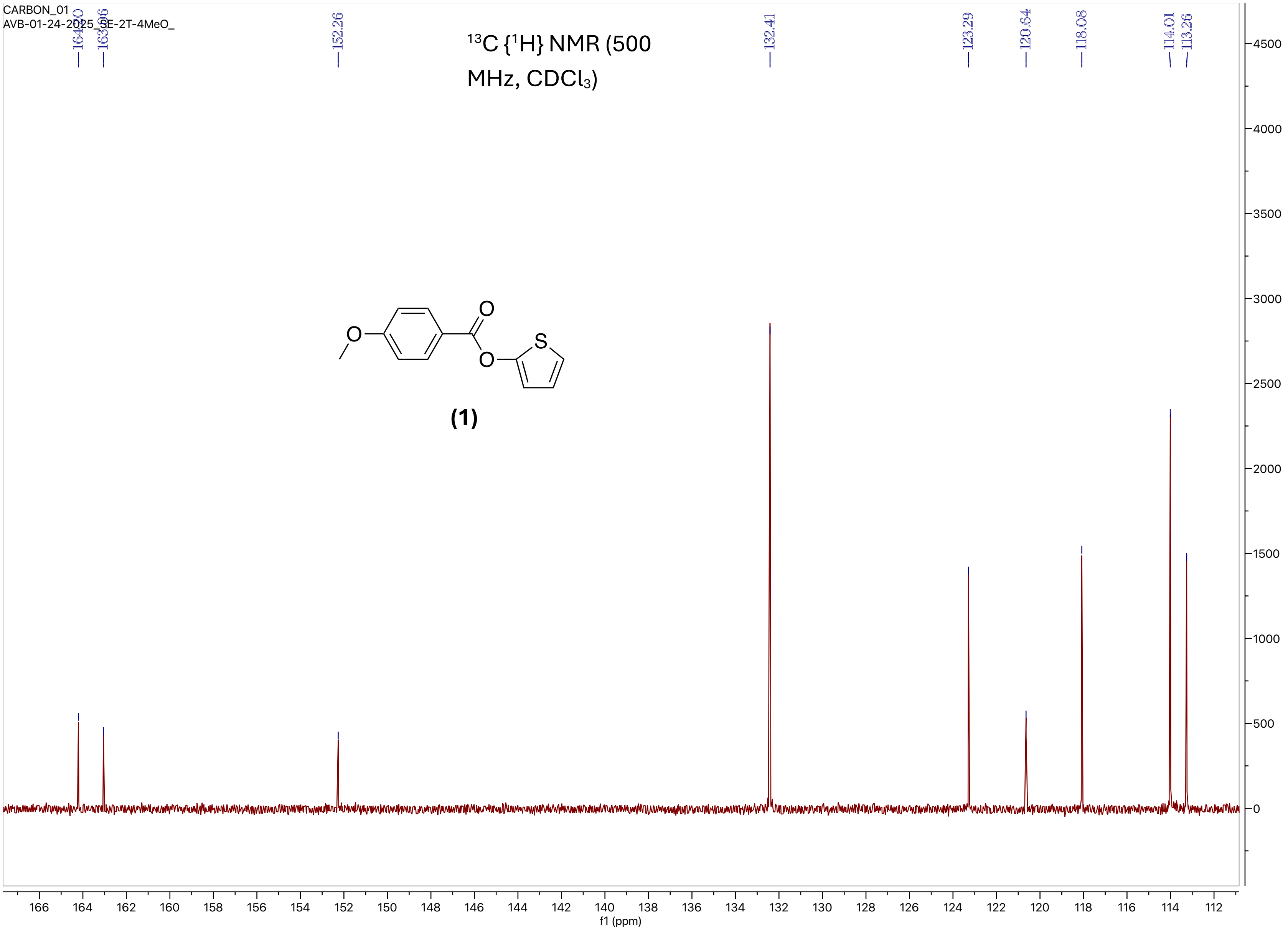
CARBON_01
AVB-01-24-2025_SE-2T-4MeO_
164.20
163.06
152.26
132.41
123.29
120.64
118.08
114.01
113.26
$^{13}C\{^{1}H\}$ NMR (500 MHz, $CDCl_3$)
O
O
O
S
(1)
4500
4000
3500
3000
2500
2000
1500
1000
500
0
166
164
162
160
158
156
154
152
150
148
146
144
142
140
138
136
134
132
130
128
126
124
122
120
118
116
114
112
f1 (ppm)

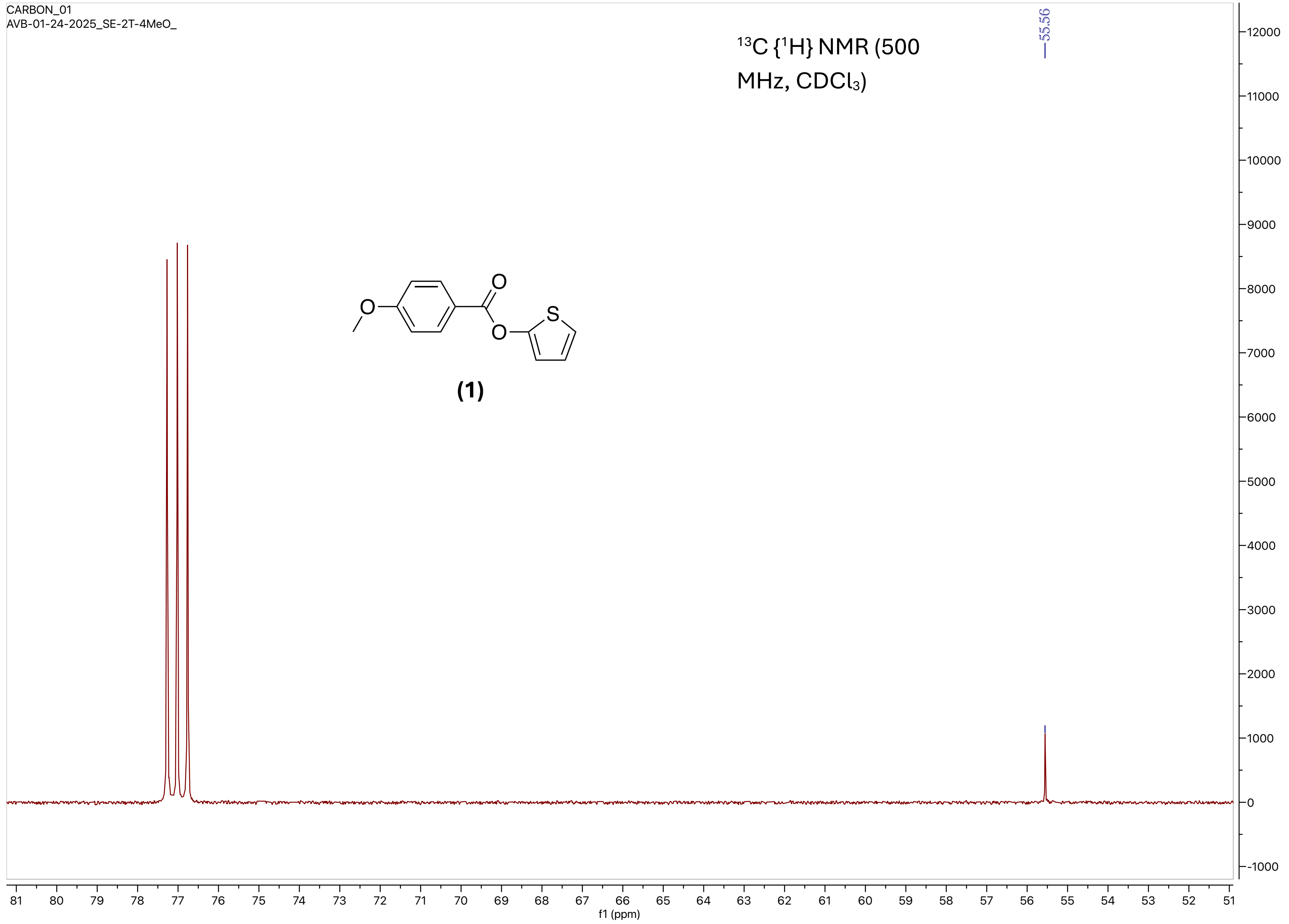
CARBON_01
AVB-01-24-2025_SE-2T-4MeO_
13C {1H} NMR (500 MHz, CDCl3)
55.56
(1)
f1 (ppm)

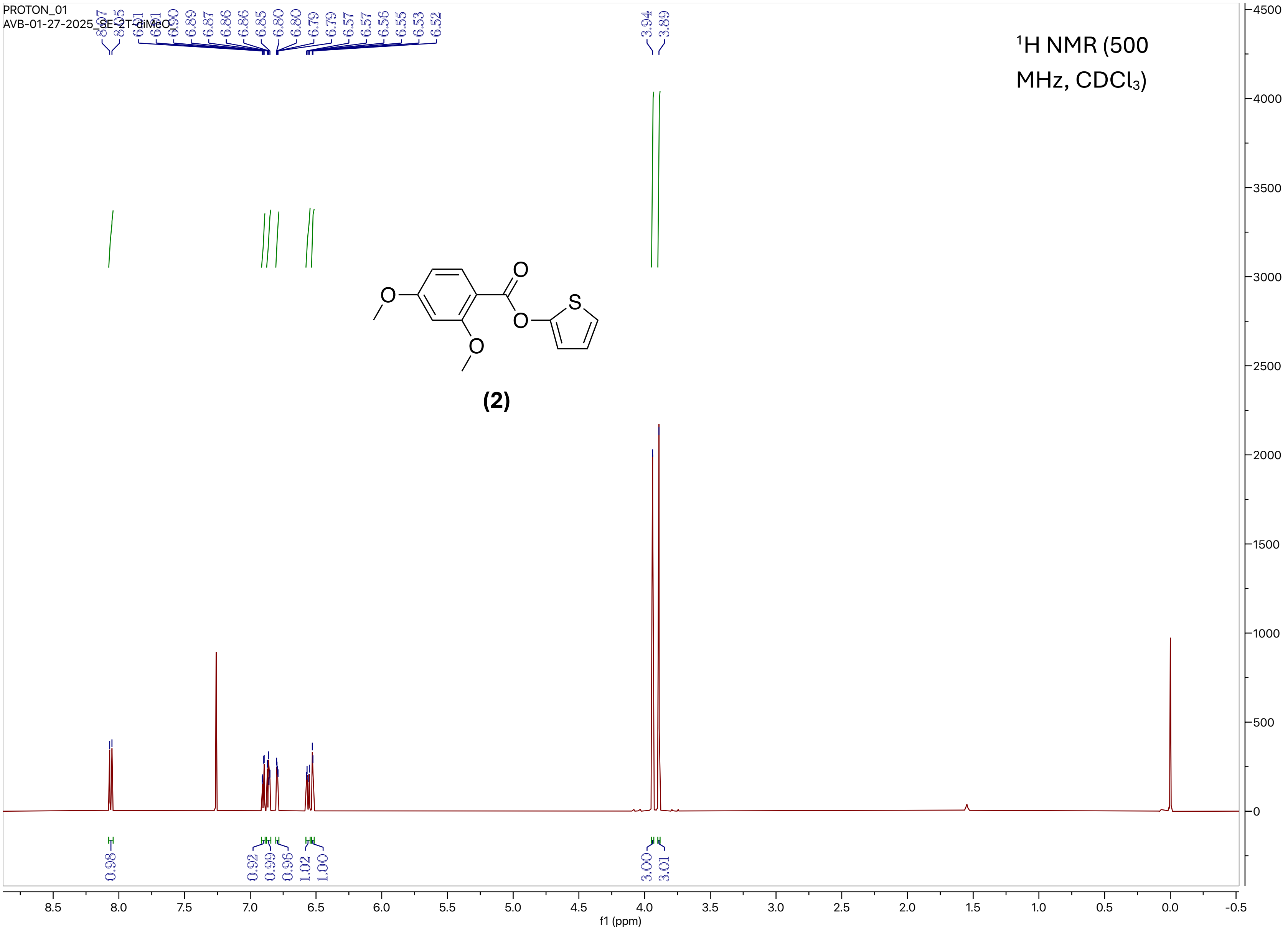
PROTON_01
AVB-01-27-2025_SE-2T-diMeO
8.07
8.05
6.91
6.91
6.90
6.89
6.87
6.86
6.86
6.85
6.80
6.80
6.79
6.79
6.57
6.56
6.55
6.53
6.52
3.94
3.89
1H NMR (500 MHz, CDCl3)
O
O
O
O
S
(2)
0.98
0.92
0.99
0.96
1.02
1.00
3.00
3.01
f1 (ppm)

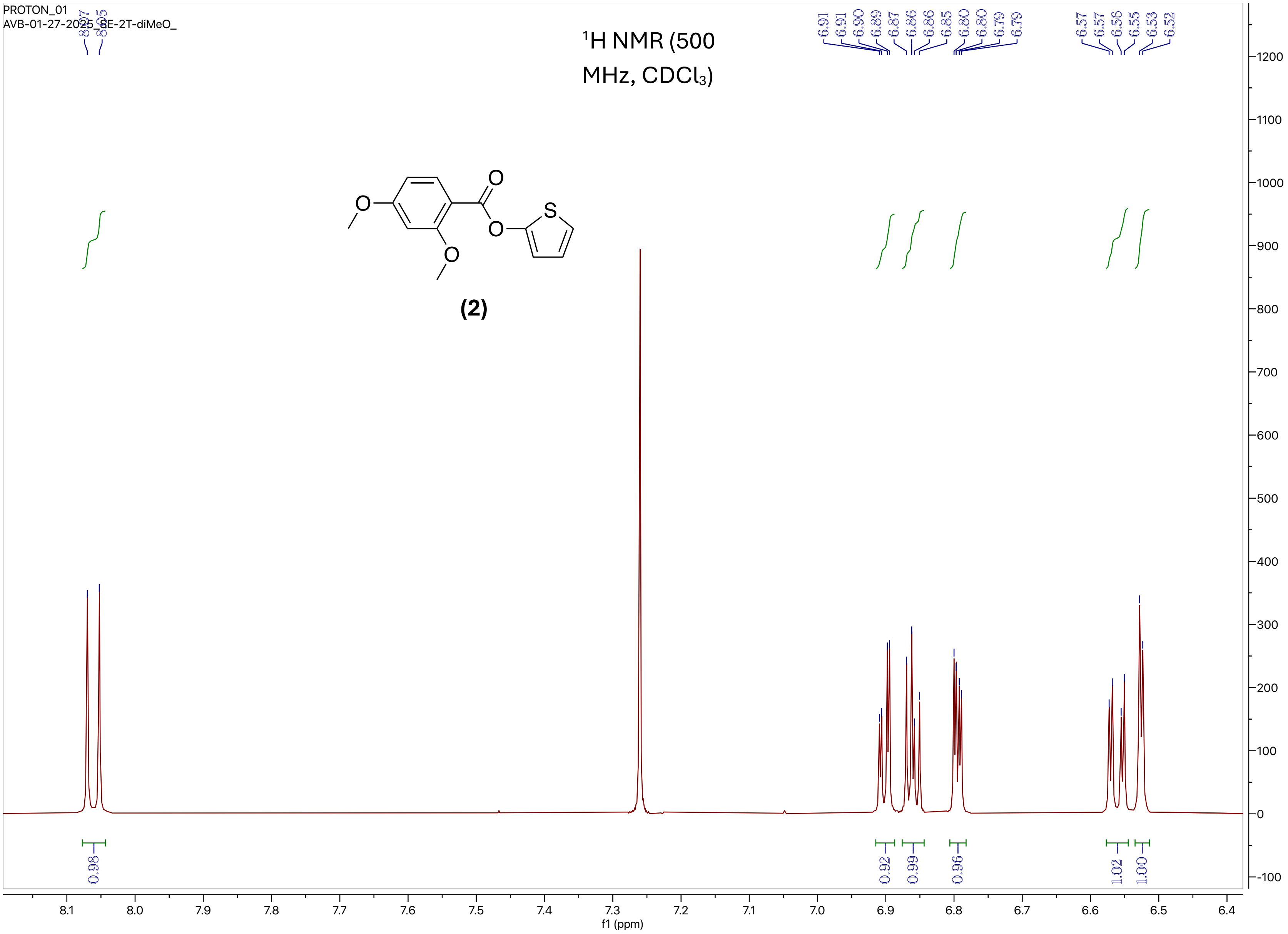
PROTON_01
AVB-01-27-2025_SE-2T-diMeO_
8.07
8.05
6.91
6.91
6.90
6.89
6.87
6.86
6.86
6.85
6.80
6.80
6.79
6.79
6.57
6.57
6.56
6.55
6.53
6.52
¹H NMR (500 MHz, CDCl₃)
O
O
O
O
S
(2)
0.98
0.92
0.99
0.96
1.02
1.00
f1 (ppm)

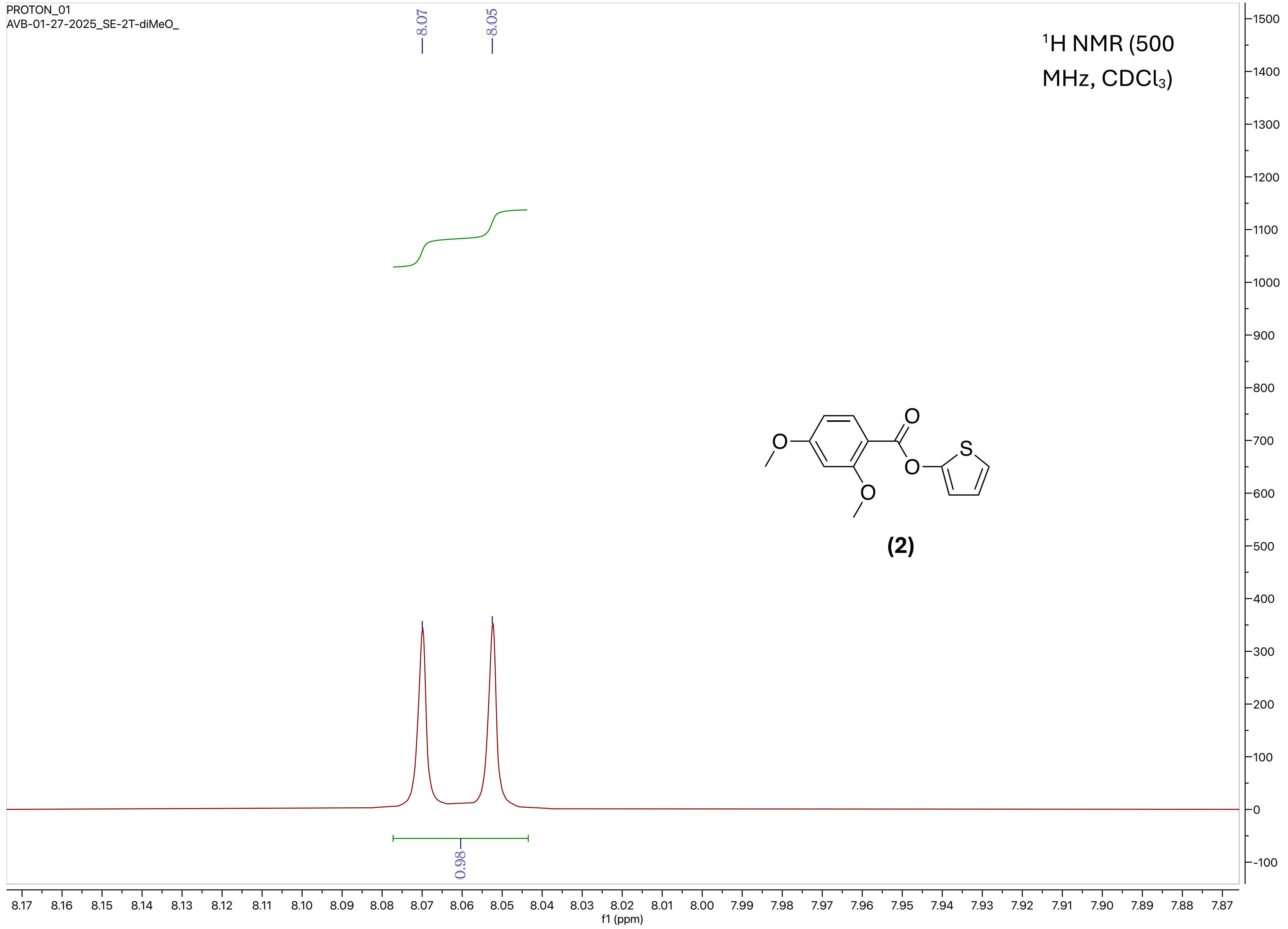

(2)

^{1}H NMR (500 MHz, $CDCl_3$)

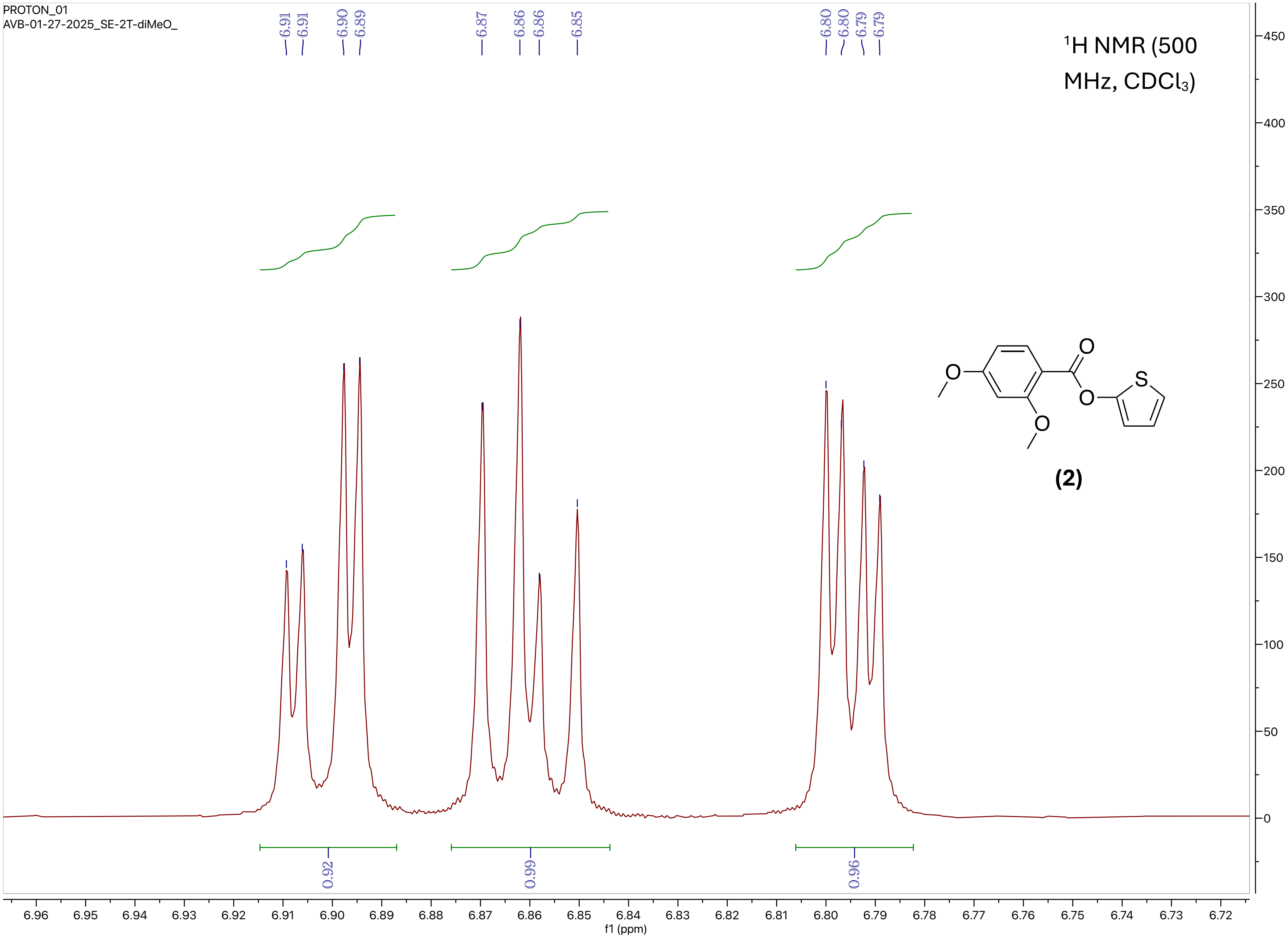

PROTON_01
AVB-01-27-2025_SE-2T-diMeO_
6.91
6.91
6.90
6.89
6.87
6.86
6.86
6.85
6.80
6.80
6.79
6.79
¹H NMR (500 MHz, CDCl₃)
O
O
S
O
O
(2)
0.92
0.99
0.96
f1 (ppm)

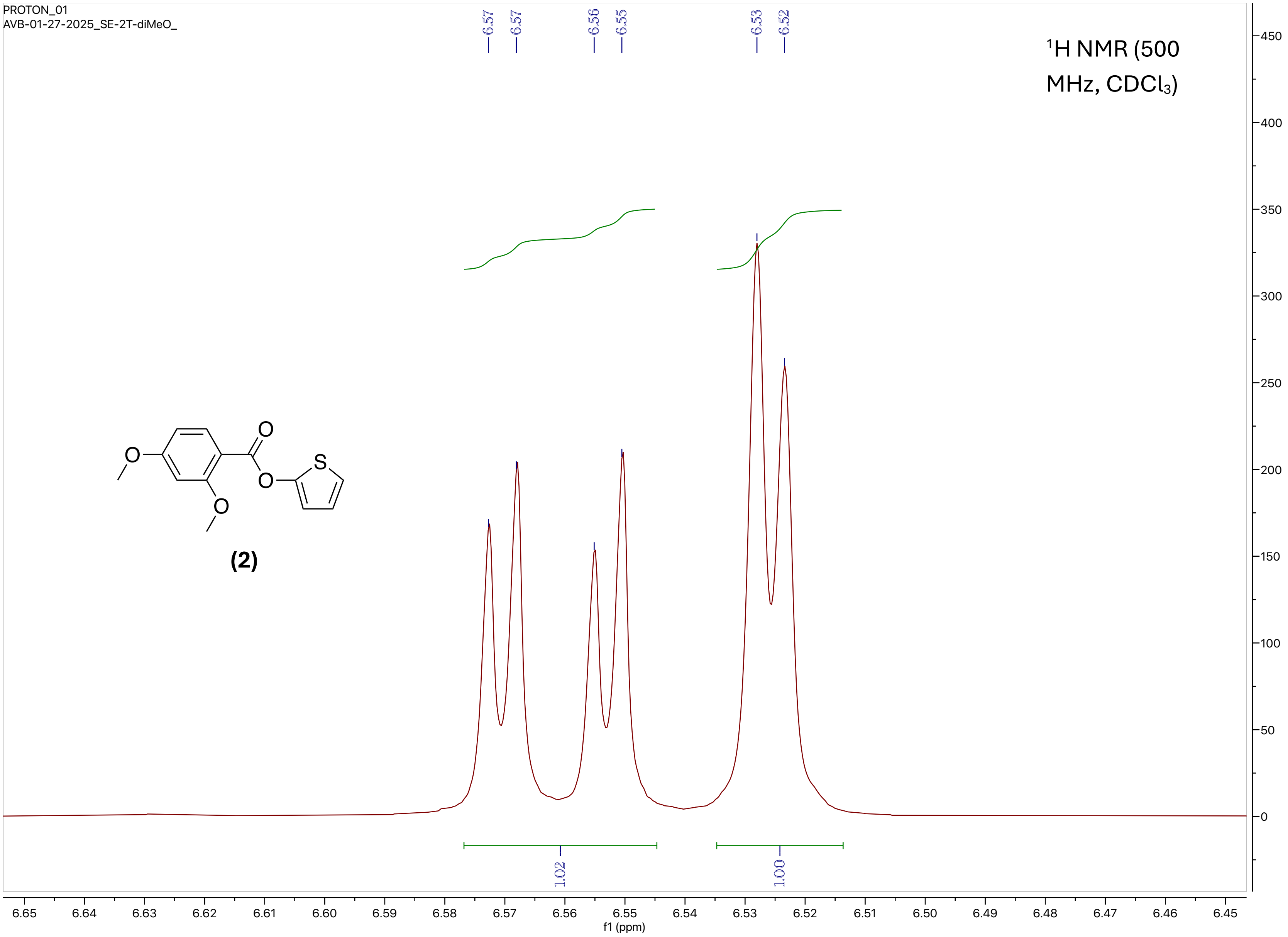
PROTON_01
AVB-01-27-2025_SE-2T-diMeO_
6.57
6.57
6.56
6.55
6.53
6.52
¹H NMR (500 MHz, CDCl₃)
(2)
1.02
1.00
f1 (ppm)

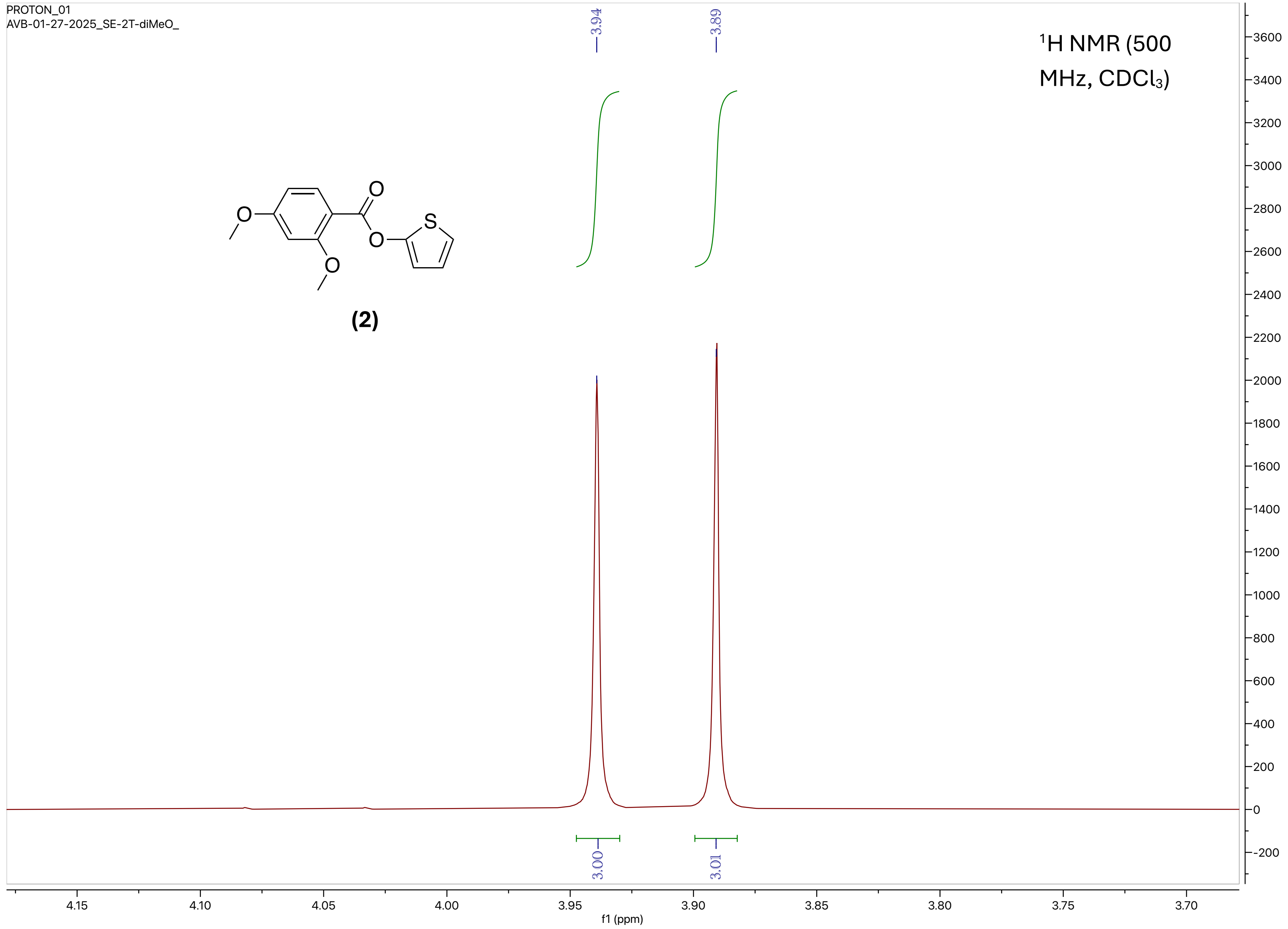
PROTON_01
AVB-01-27-2025_SE-2T-diMeO_
3.94
3.89
^{1}H NMR (500 MHz, $CDCl_3$)
(2)
3.00
3.01
f1 (ppm)

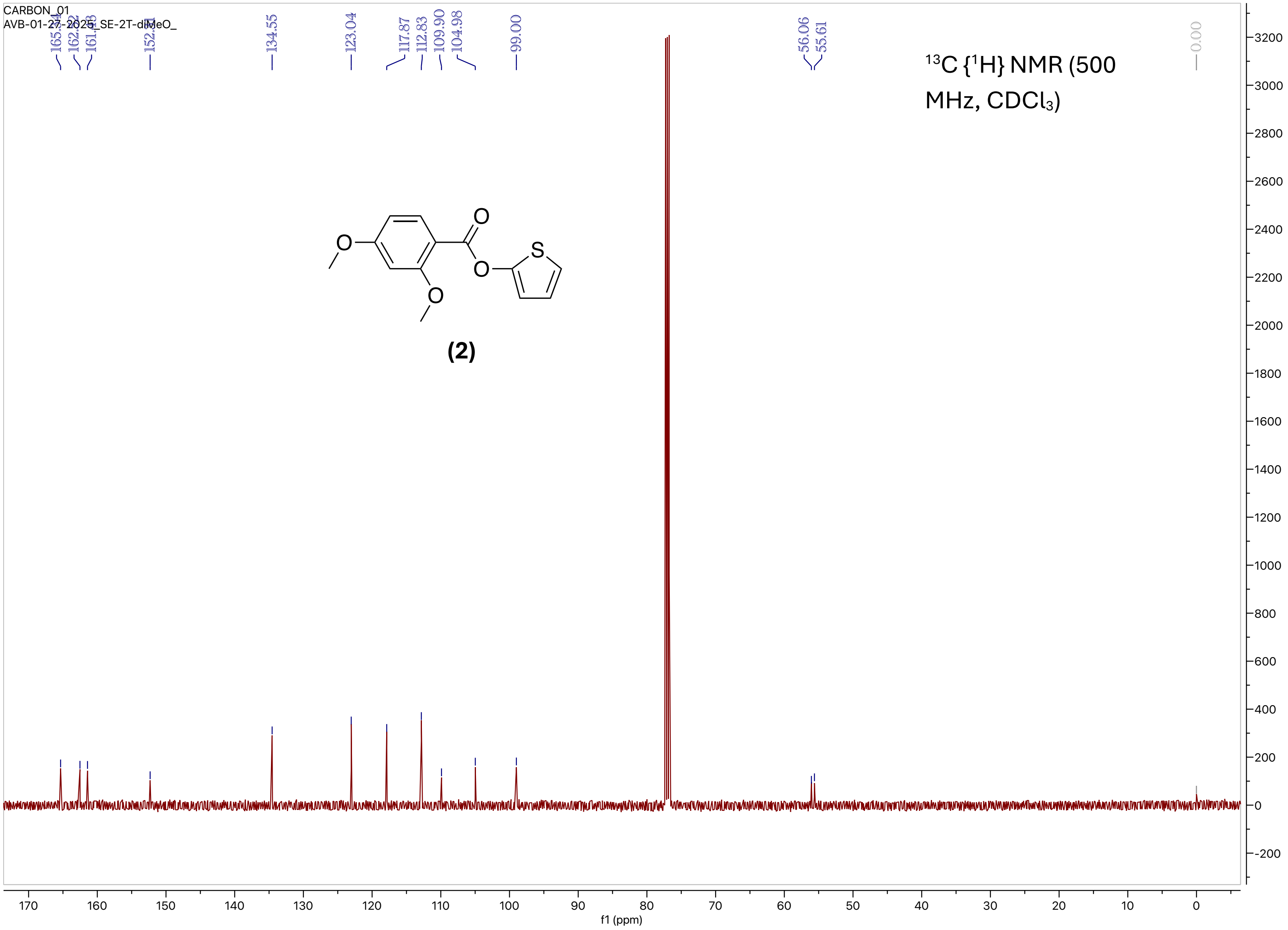
CARBON_01
AVB-01-27-2025_SE-2T-diMeO_
165.44
162.72
161.53
152.71
134.55
123.04
117.87
112.83
109.90
104.98
99.00
56.06
55.61
0.00
$^{13}C\{^1H\}$ NMR (500 MHz, $CDCl_3$)
(2)
f1 (ppm)

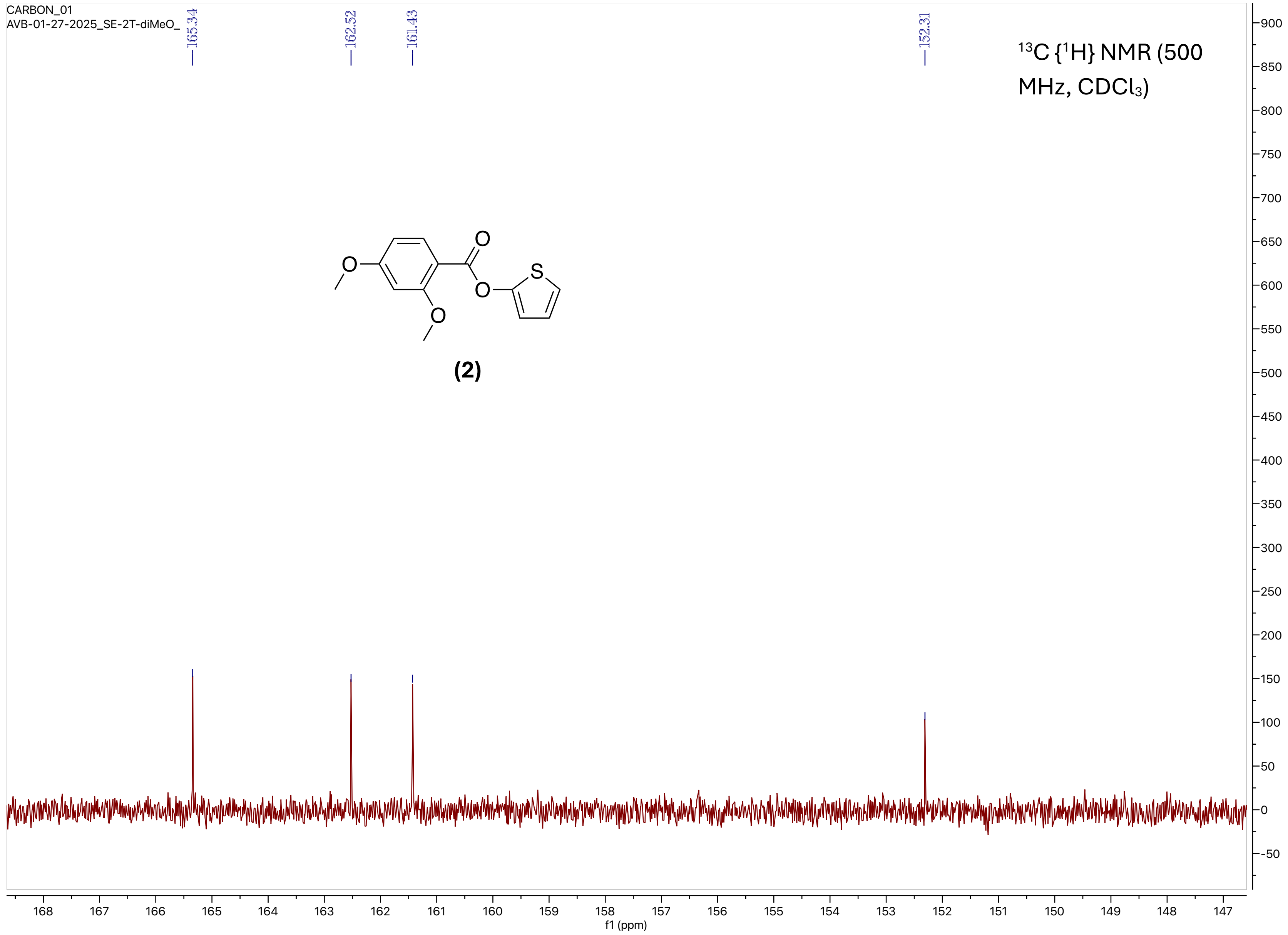
CARBON_01
AVB-01-27-2025_SE-2T-diMeO_
165.34
162.52
161.43
152.31
13C {1H} NMR (500 MHz, CDCl3)
(2)
f1 (ppm)

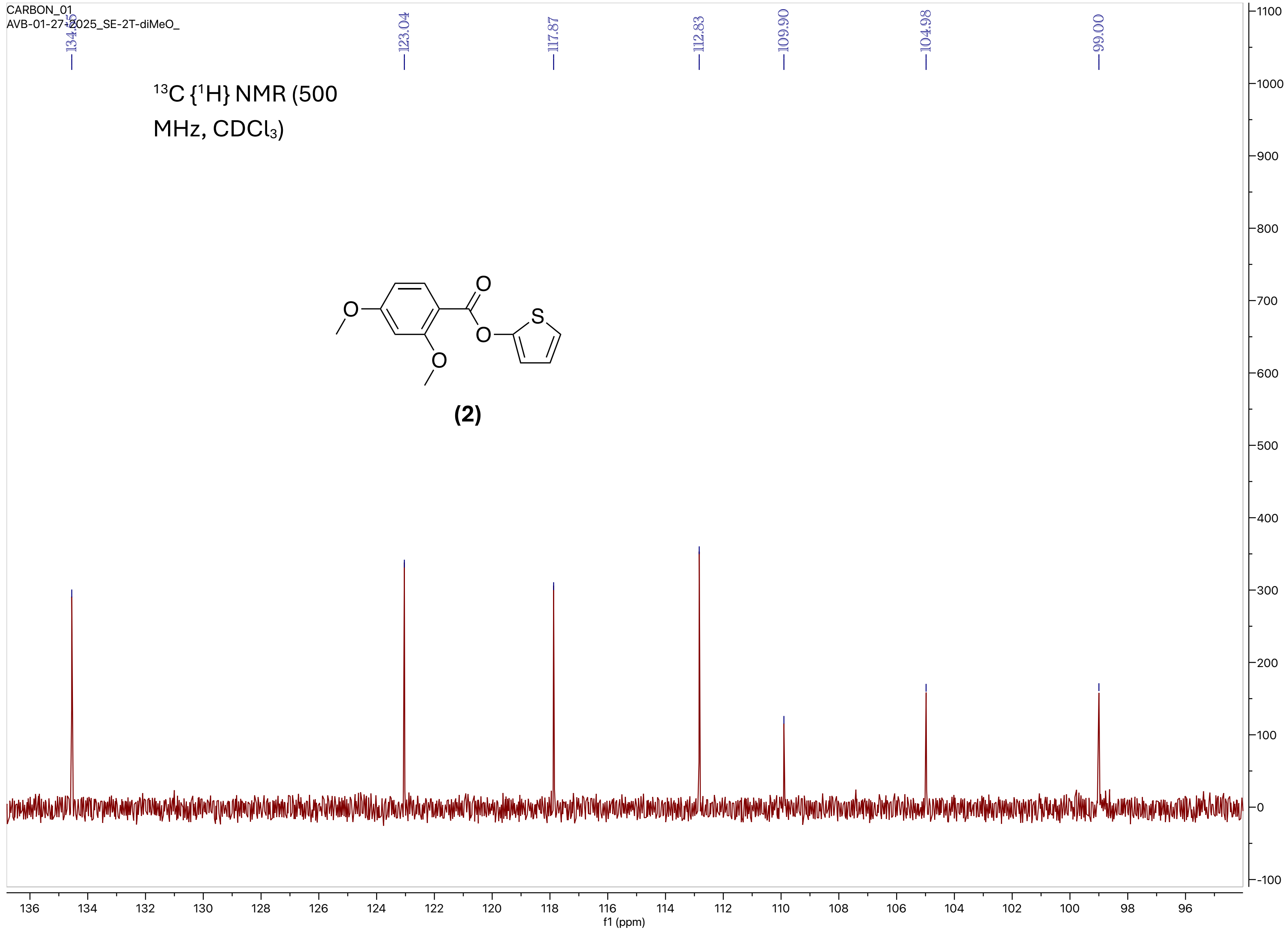

CARBON_01
AVB-01-27-2025_SE-2T-diMeO_
134.45
123.04
117.87
112.83
109.90
104.98
99.00
$^{13}C\{^1H\}$ NMR (500 MHz, $CDCl_3$)
(2)
f1 (ppm)

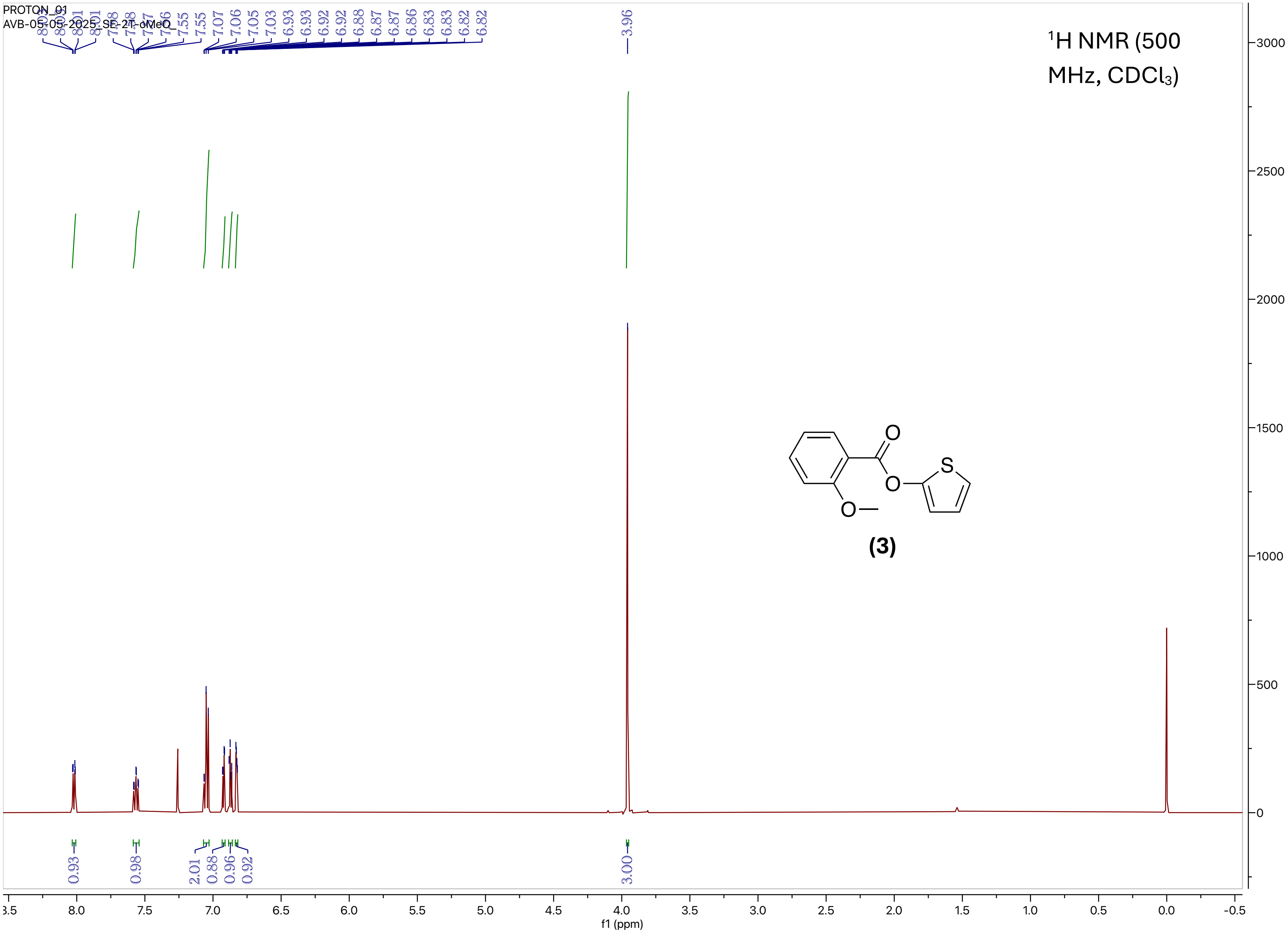
PROTON_01
AVB-05-05-2025_SE-2T-oMeO_
^{1}H NMR (500 MHz, $CDCl_3$)
O
S
O
O
(3)
f1 (ppm)

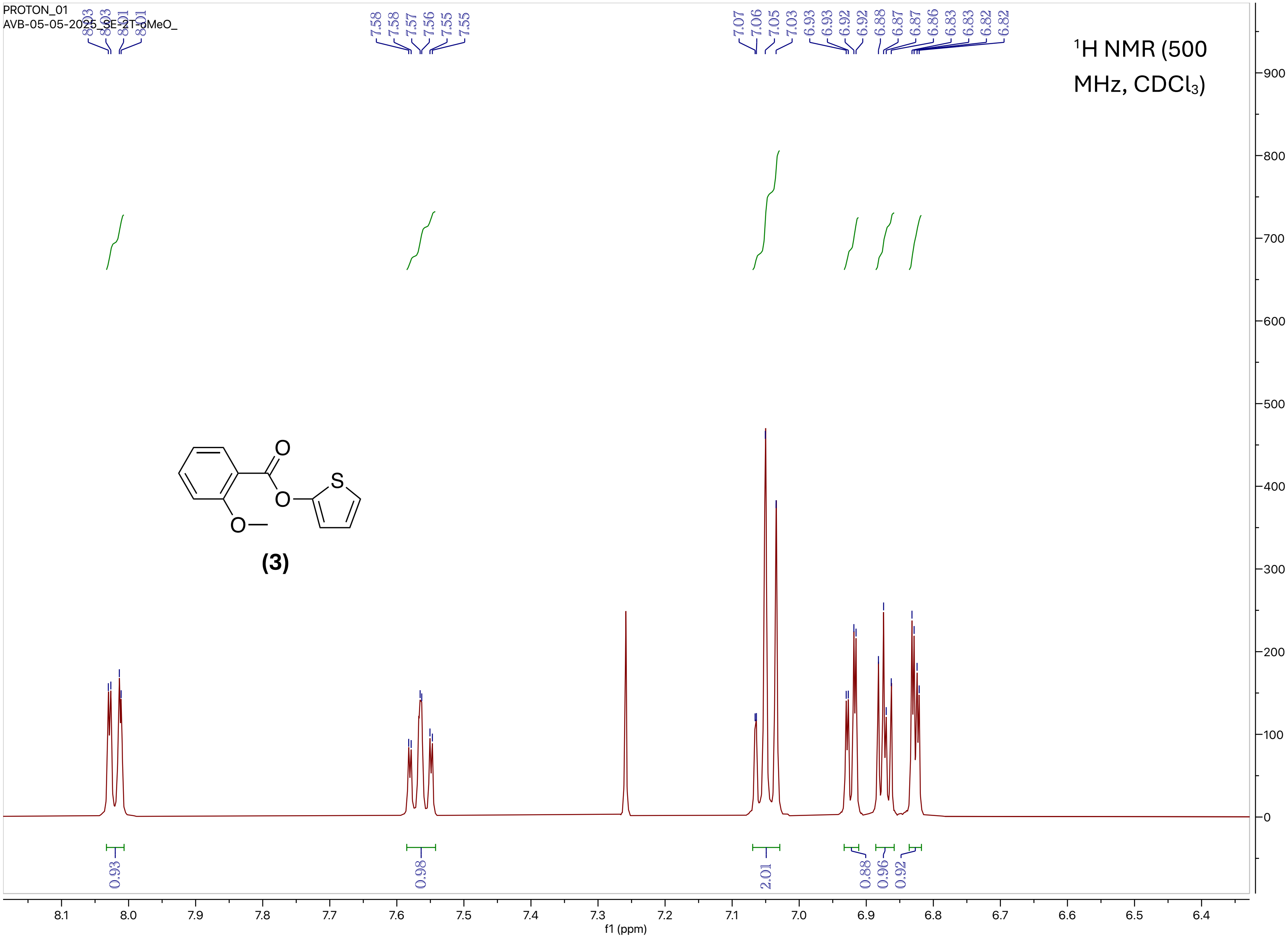
PROTON_01
AVB-05-05-2025_SE-2T-oMeO_
8.03
8.03
8.01
8.01
7.58
7.58
7.57
7.56
7.55
7.55
7.07
7.06
7.05
7.03
6.93
6.93
6.92
6.92
6.88
6.87
6.87
6.86
6.83
6.83
6.82
6.82
^{1}H NMR (500 MHz, $CDCl_3$)
O
O
S
O
(3)
0.93
0.98
2.01
0.88
0.96
0.92
f1 (ppm)

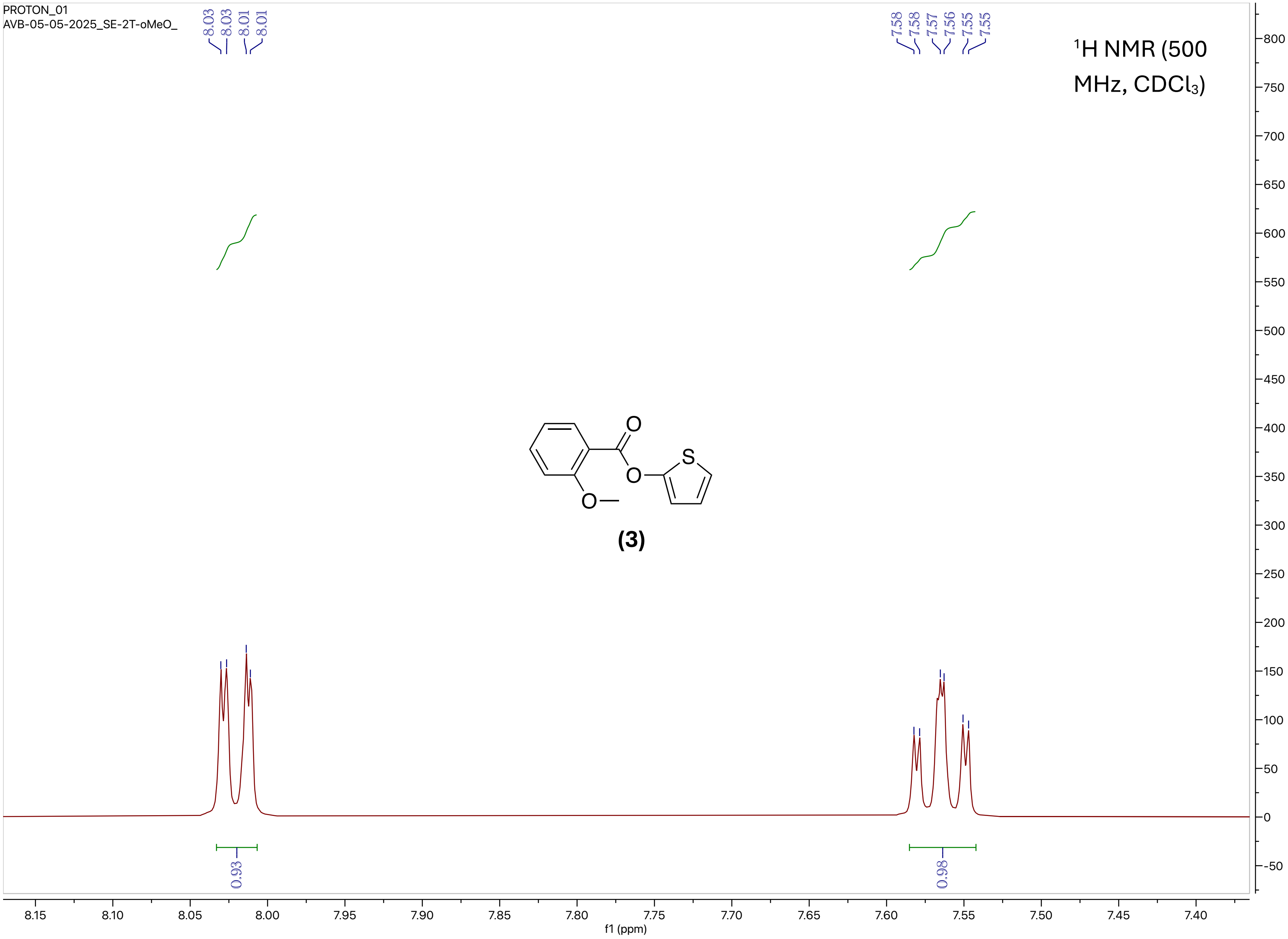
PROTON_01
AVB-05-05-2025_SE-2T-oMeO_
8.03
8.03
8.01
8.01
7.58
7.58
7.57
7.56
7.55
7.55
^{1}H NMR (500 MHz, CDCl$_3$)
0.93
0.98
f1 (ppm)

(3)

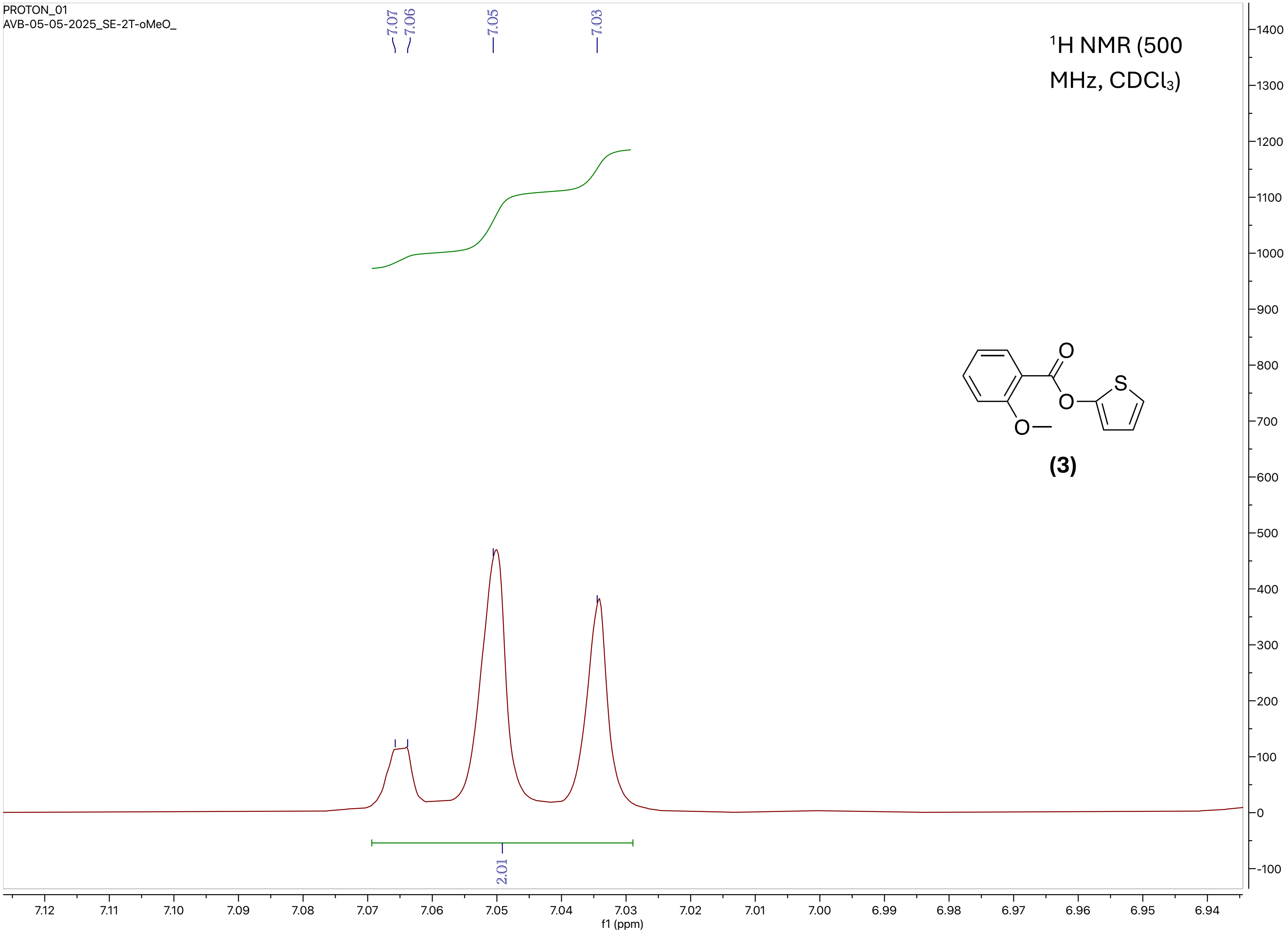

PROTON_01
AVB-05-05-2025_SE-2T-oMeO_
7.07
7.06
7.05
7.03
1H NMR (500 MHz, CDCl3)
O
S
O
O
(3)
2.01
f1 (ppm)

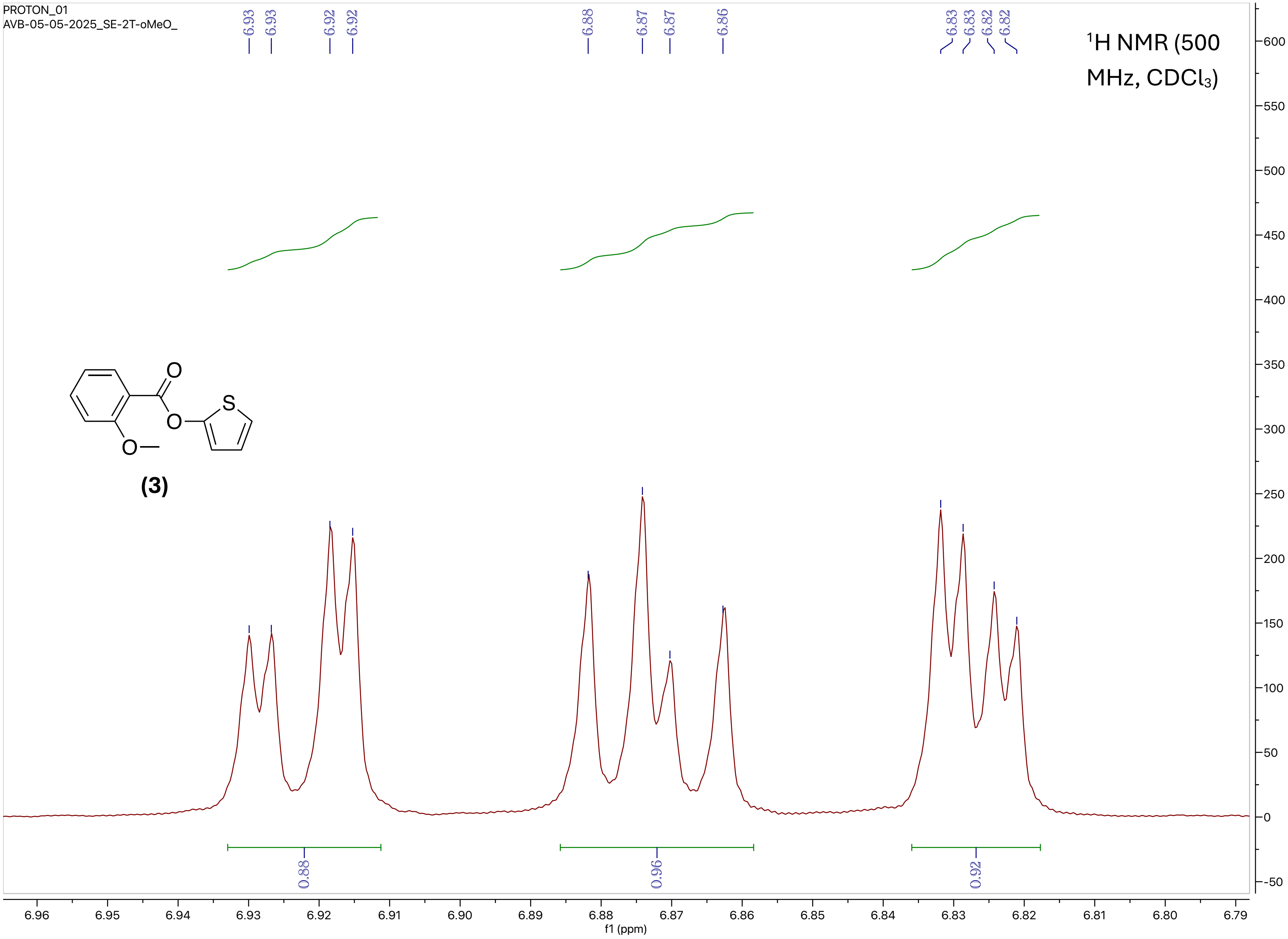
PROTON_01
AVB-05-05-2025_SE-2T-oMeO_
6.93
6.93
6.92
6.92
6.88
6.87
6.87
6.86
6.83
6.83
6.82
6.82
¹H NMR (500 MHz, CDCl₃)
0.88
0.96
0.92
f1 (ppm)

(3)

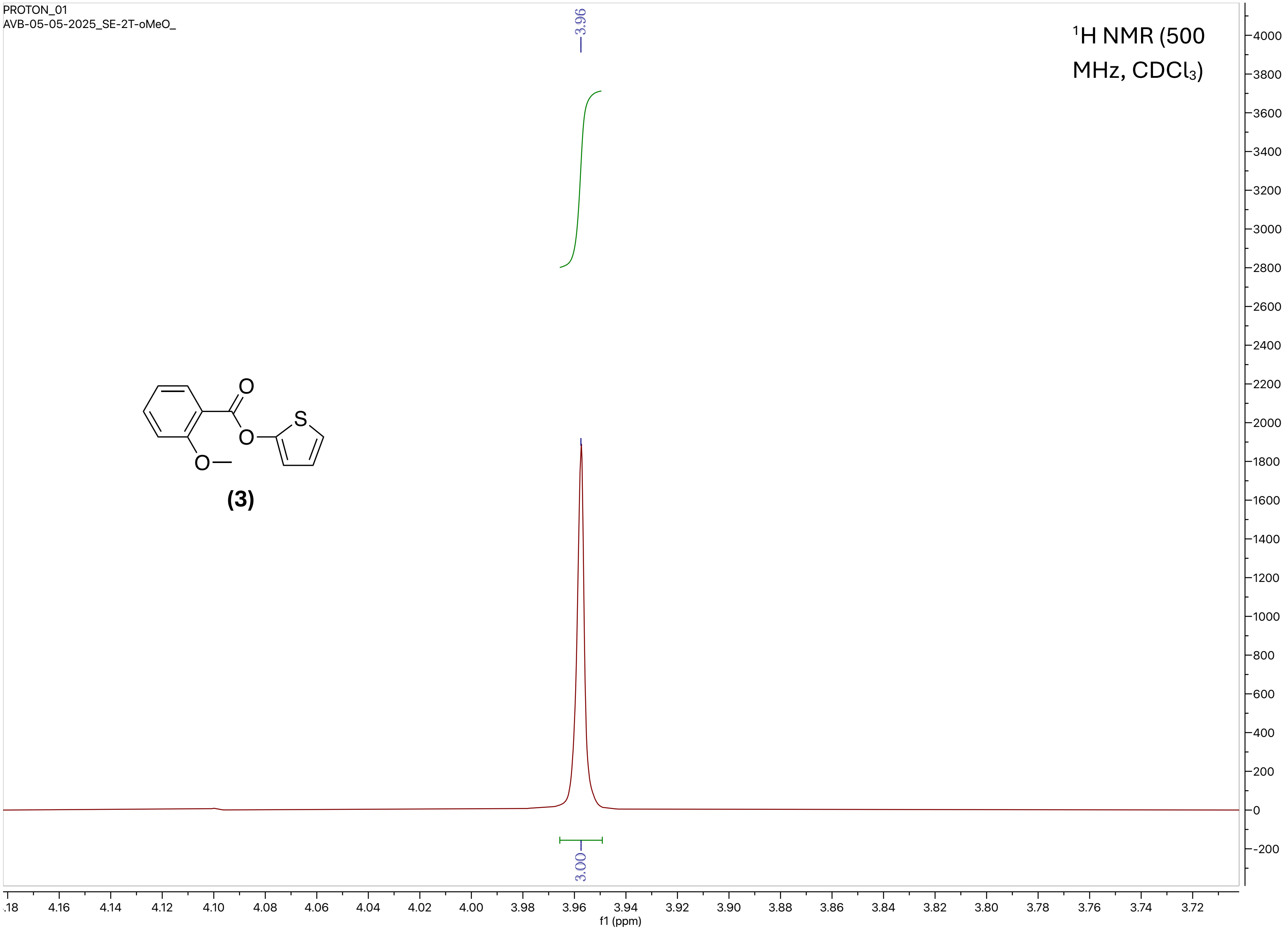
PROTON_01
AVB-05-05-2025_SE-2T-oMeO_
—3.96
¹H NMR (500
MHz, CDCl₃)
O
S
O
O
(3)
3.00
f1 (ppm)

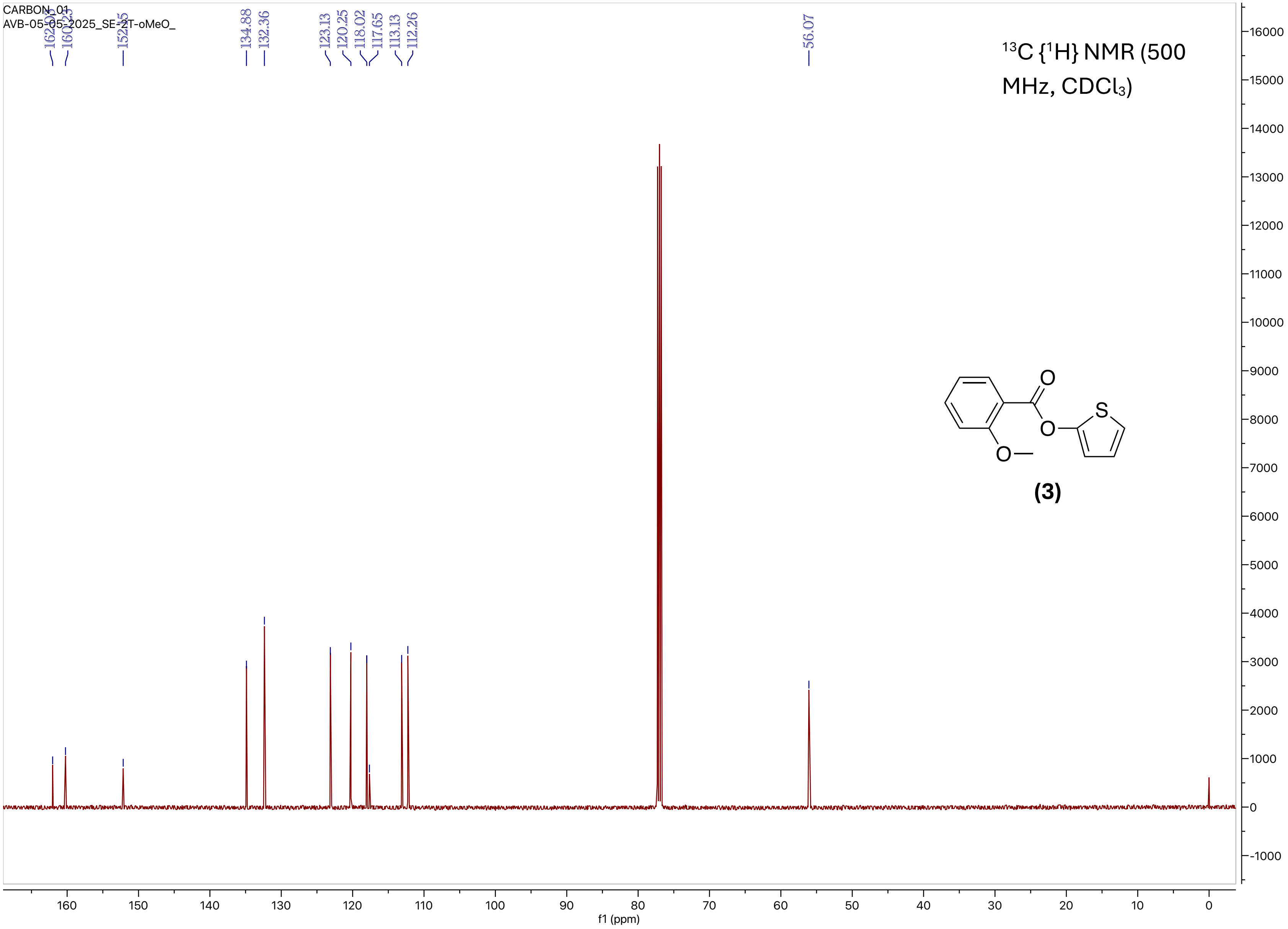
CARBON_01
AVB-05-05-2025_SE-2T-oMeO_
162.03
160.25
152.15
134.88
132.36
123.13
120.25
118.02
117.65
113.13
112.26
56.07
13C {1H} NMR (500 MHz, CDCl3)
O
O
S
O
(3)
f1 (ppm)

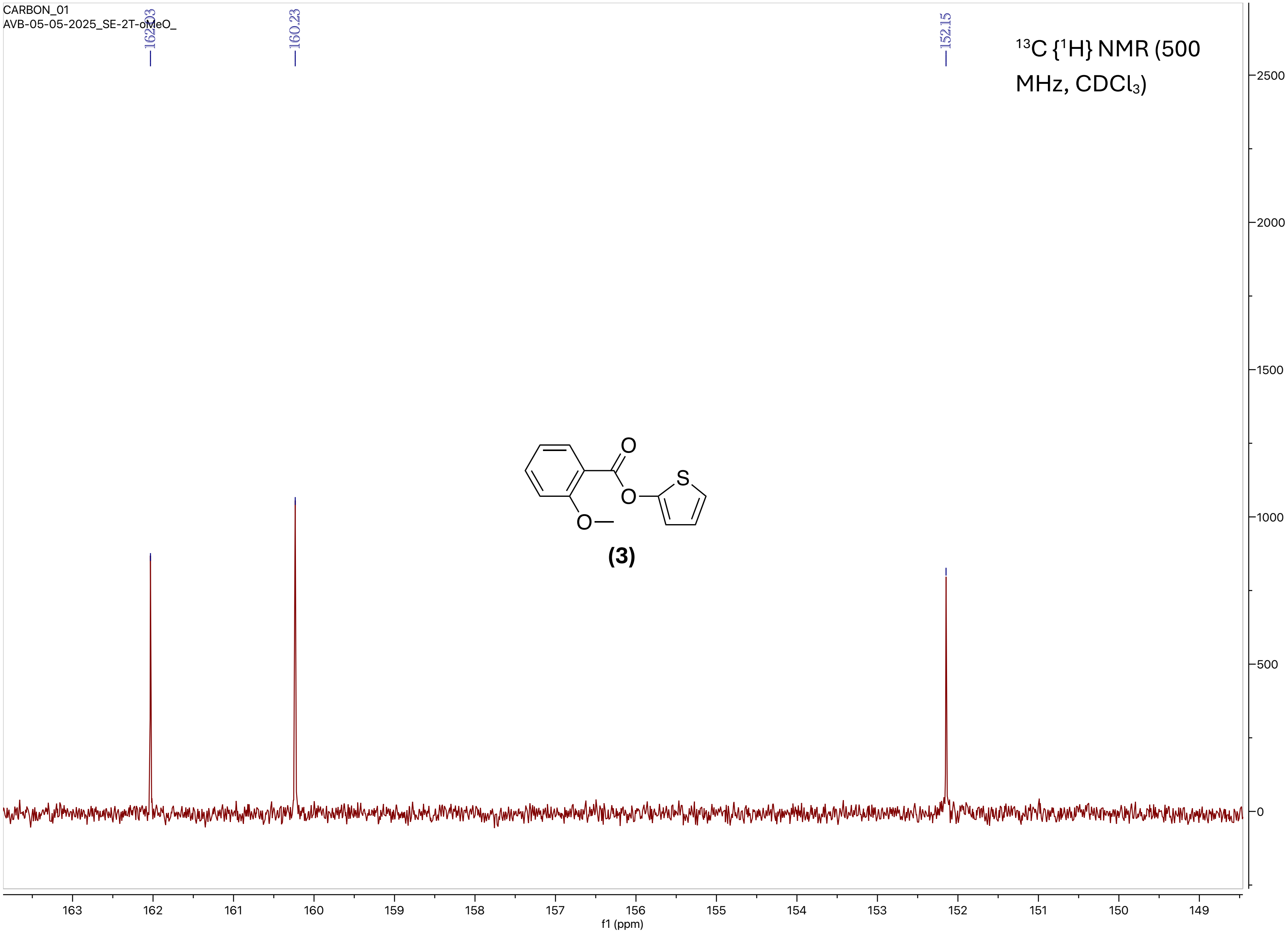
CARBON_01
AVB-05-05-2025_SE-2T-oMeO_
162.03
160.23
152.15
13C {1H} NMR (500 MHz, CDCl3)
O
O
S
O
(3)
2500
2000
1500
1000
500
0
163
162
161
160
159
158
157
156
155
154
153
152
151
150
149
f1 (ppm)

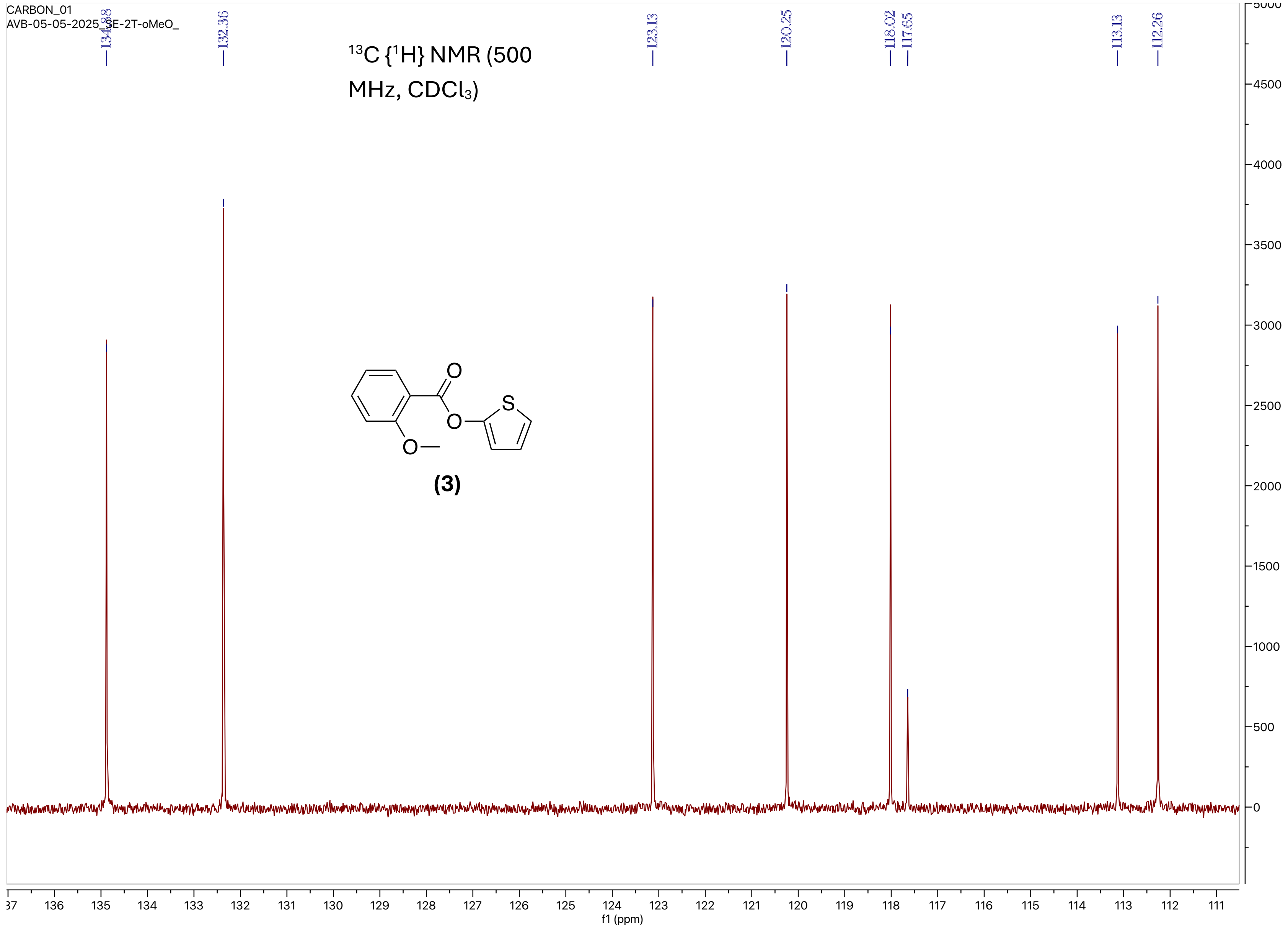
CARBON_01
AVB-05-05-2025_SE-2T-oMeO_
134.88
132.36
123.13
120.25
118.02
117.65
113.13
112.26
13C {1H} NMR (500
MHz, CDCl3)
O
O
S
O
(3)
f1 (ppm)

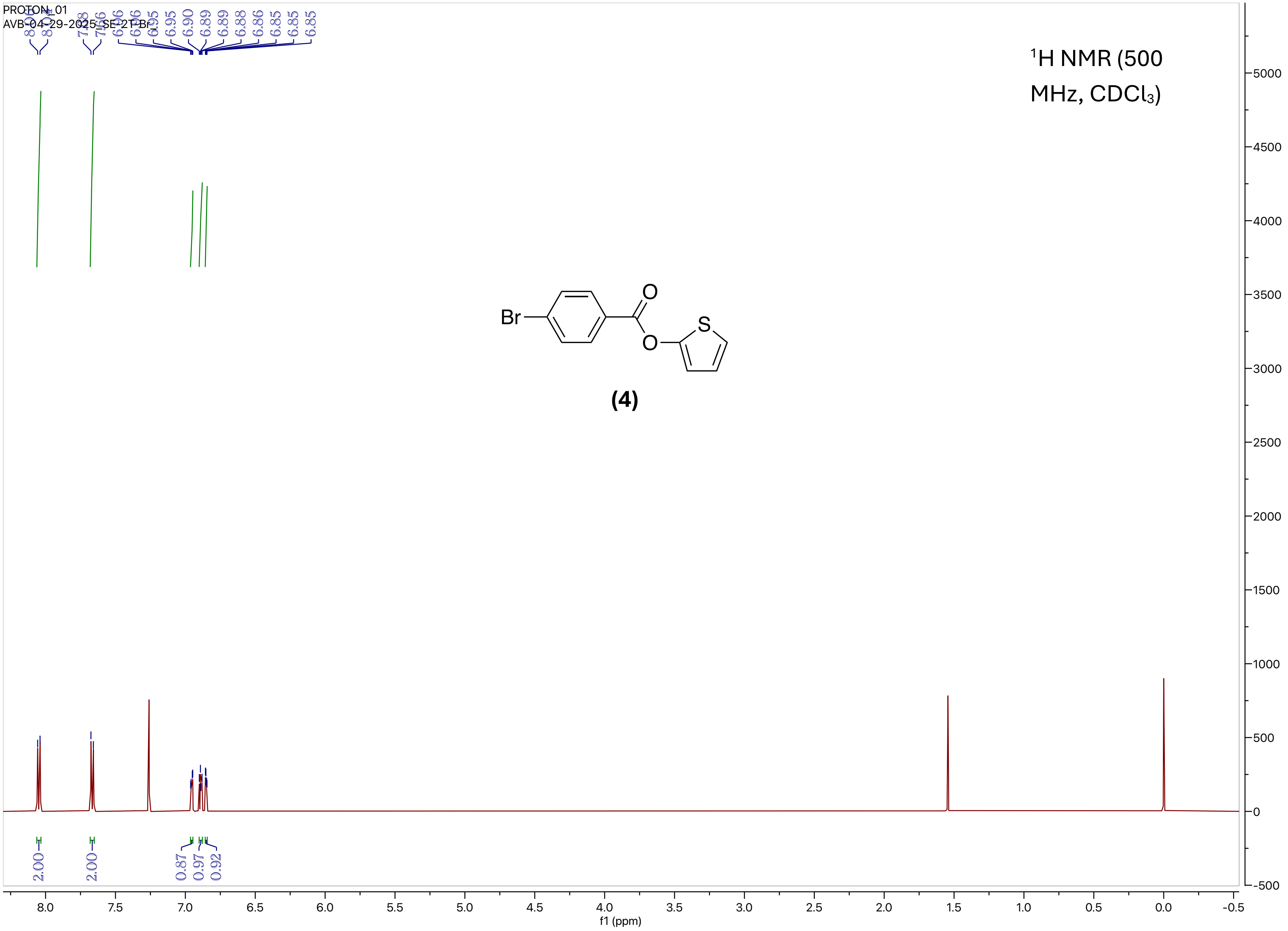
PROTON_01
AVB-04-29-2025_SE-2T-Br
^{1}H NMR (500 MHz, CDCl3)
Br
O
O
S
(4)
f1 (ppm)
2.00
2.00
0.87
0.97
0.92

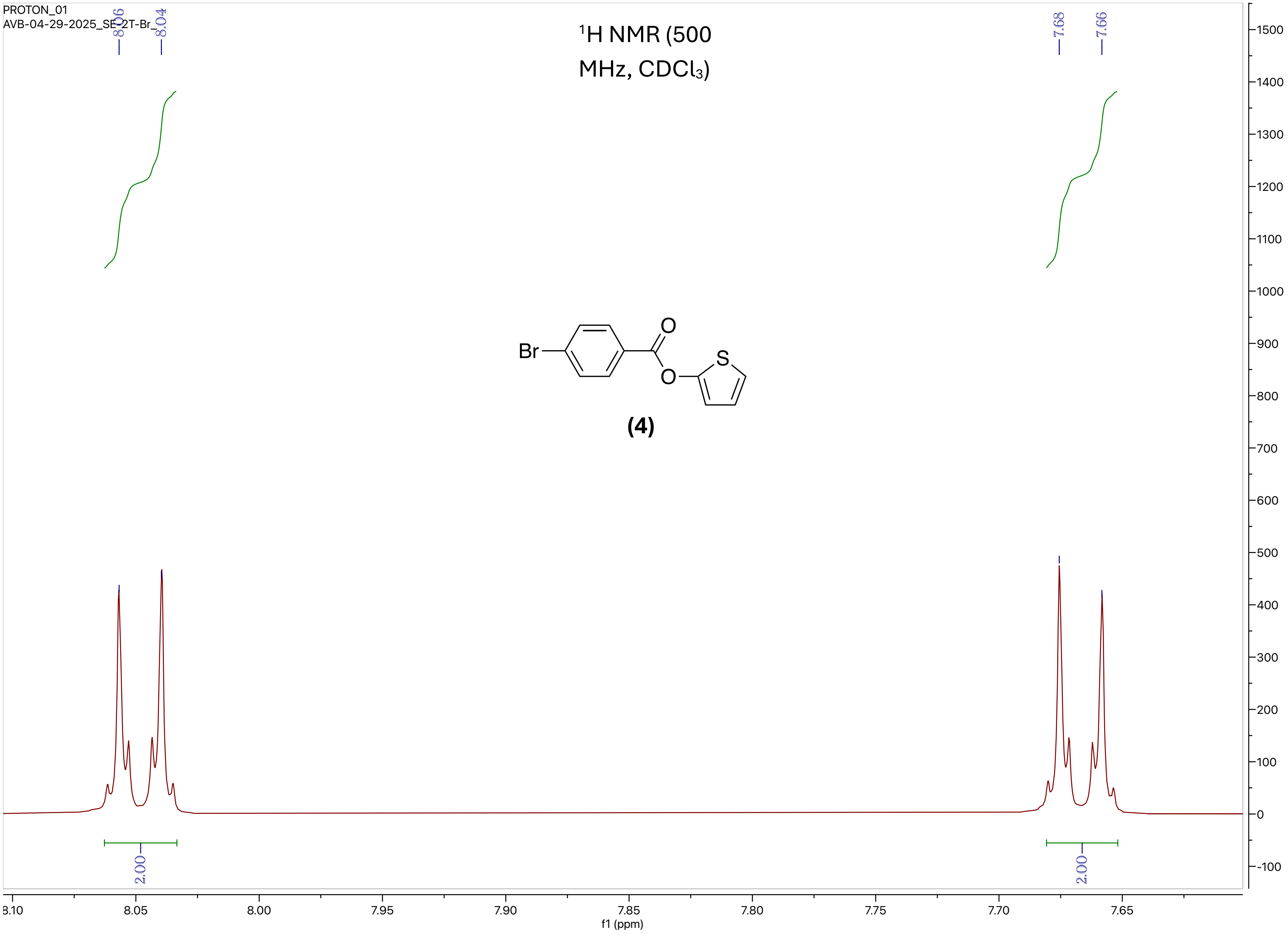
PROTON_01
AVB-04-29-2025_SE-2T-Br_
8.06
8.04
7.68
7.66
1H NMR (500 MHz, CDCl3)
Br
O
O
S
(4)
2.00
2.00
f1 (ppm)

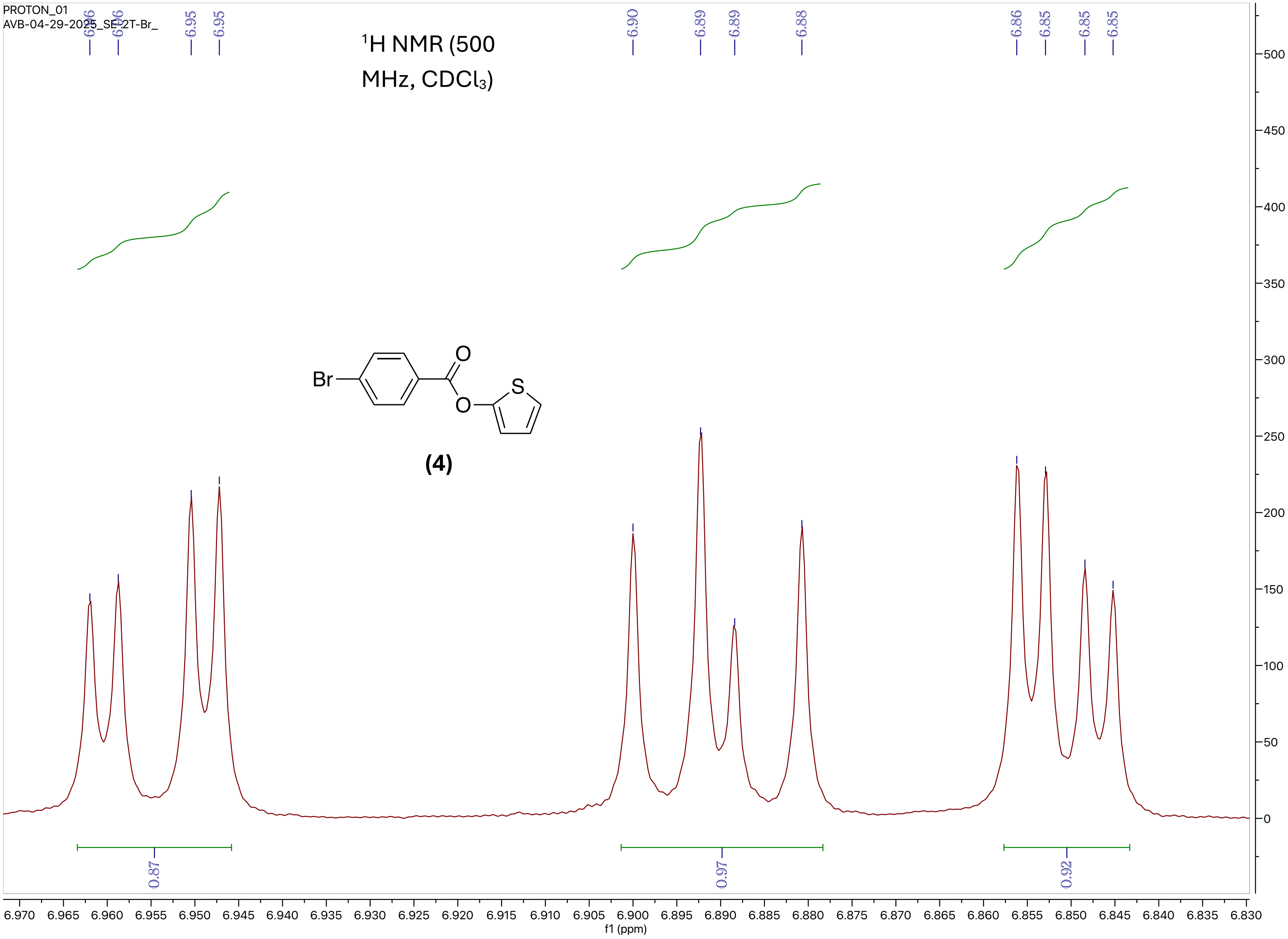
PROTON_01
AVB-04-29-2025_SE-2T-Br_
6.96
6.96
6.95
6.95
6.90
6.89
6.89
6.88
6.86
6.85
6.85
6.85
^{1}H NMR (500 MHz, CDCl$_3$)
Br
O
O
S
(4)
0.87
0.97
0.92
f1 (ppm)

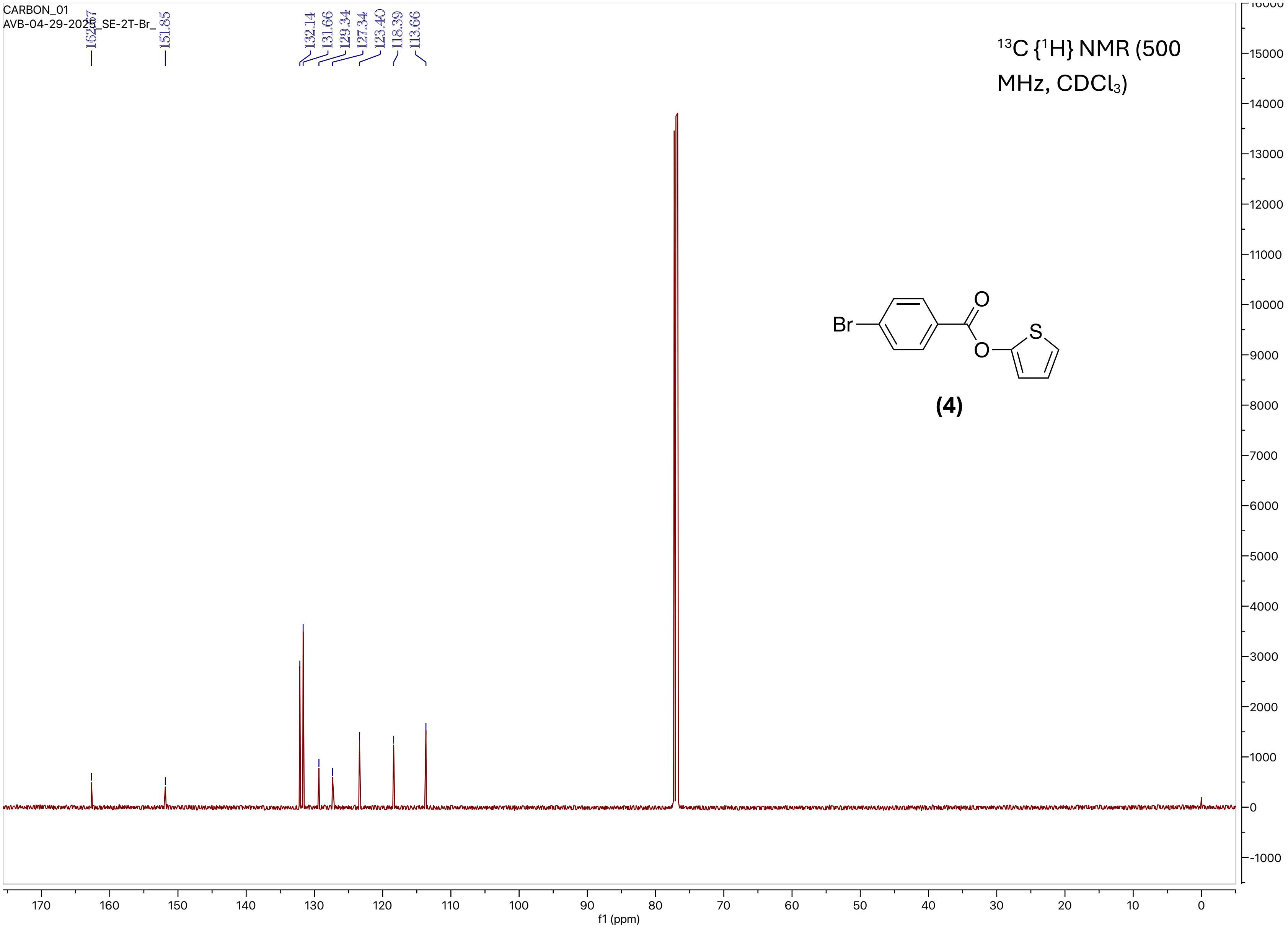
CARBON_01
AVB-04-29-2025_SE-2T-Br_
162.67
151.85
132.14
131.66
129.34
127.34
123.40
118.39
113.66
13C {1H} NMR (500 MHz, CDCl3)
Br
O
O
S
(4)
f1 (ppm)

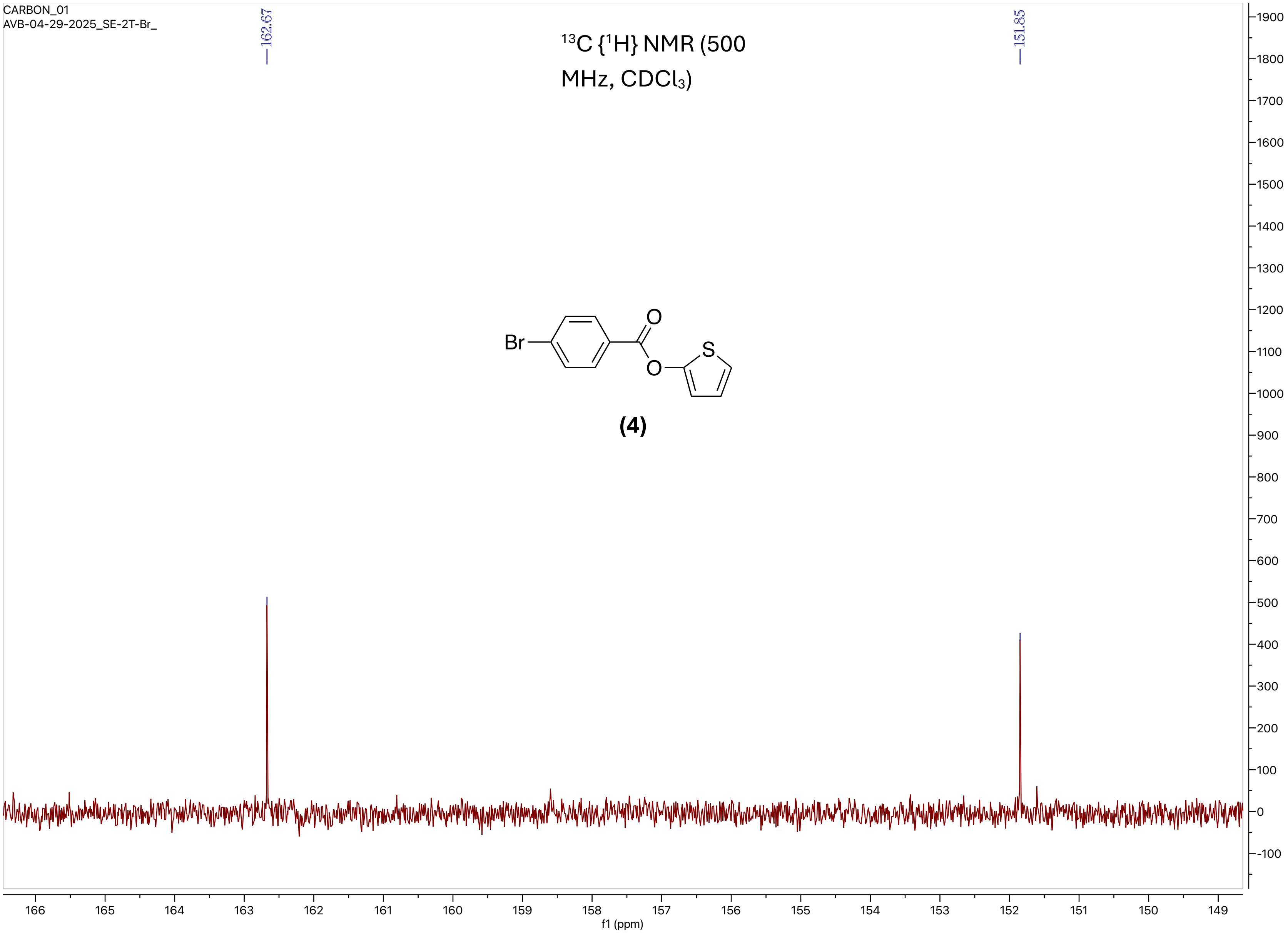
CARBON_01
AVB-04-29-2025_SE-2T-Br_
162.67
151.85
$^{13}C\{^1H\}$ NMR (500 MHz, $CDCl_3$)
Br
O
O
S
f1 (ppm)

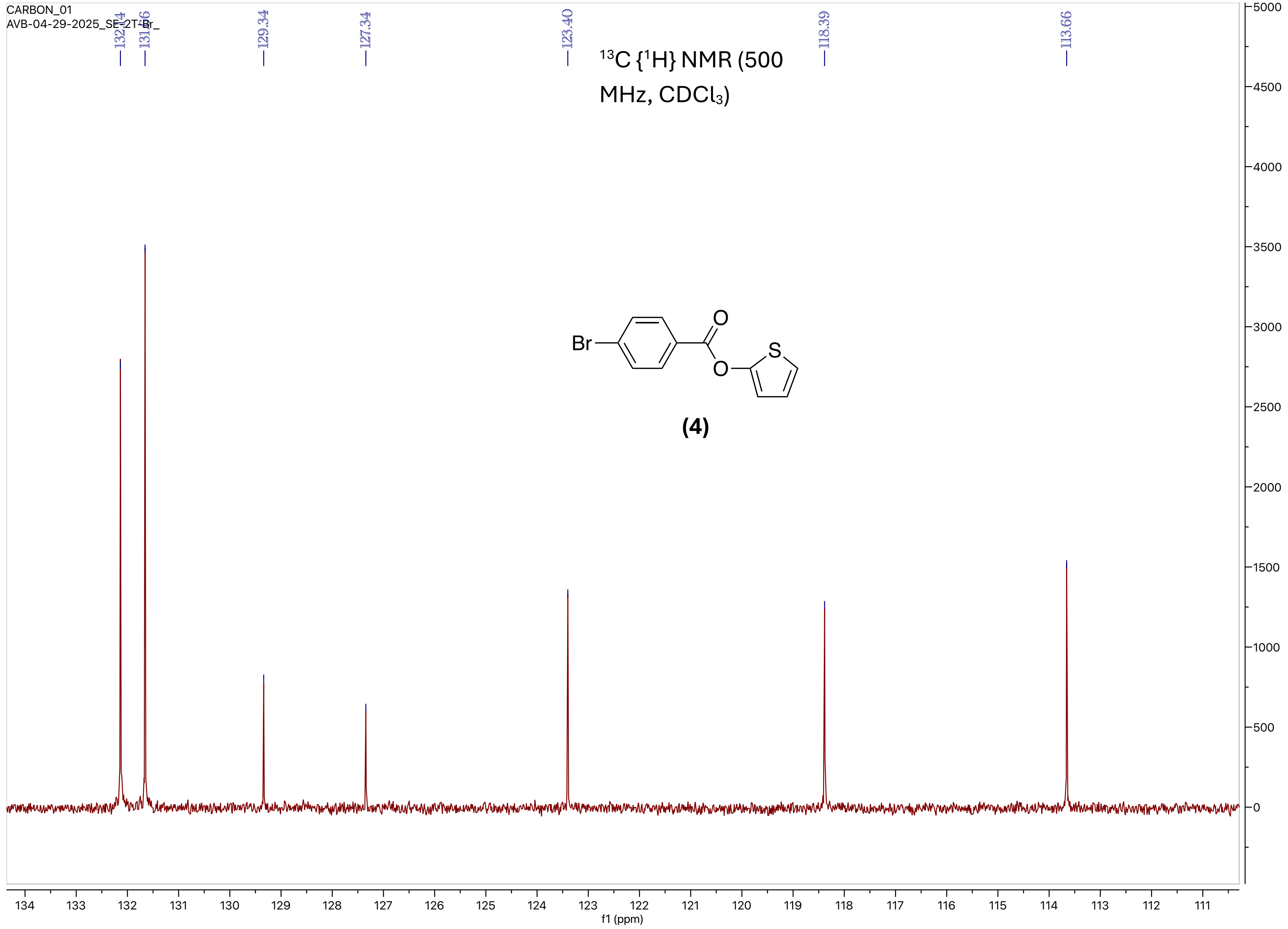
CARBON_01
AVB-04-29-2025_SE-2T-Br_
132.14
131.66
129.34
127.34
123.40
118.39
113.66
^{13}C {^{1}H} NMR (500 MHz, CDCl$_3$)
Br
O
O
S
(4)
f1 (ppm)

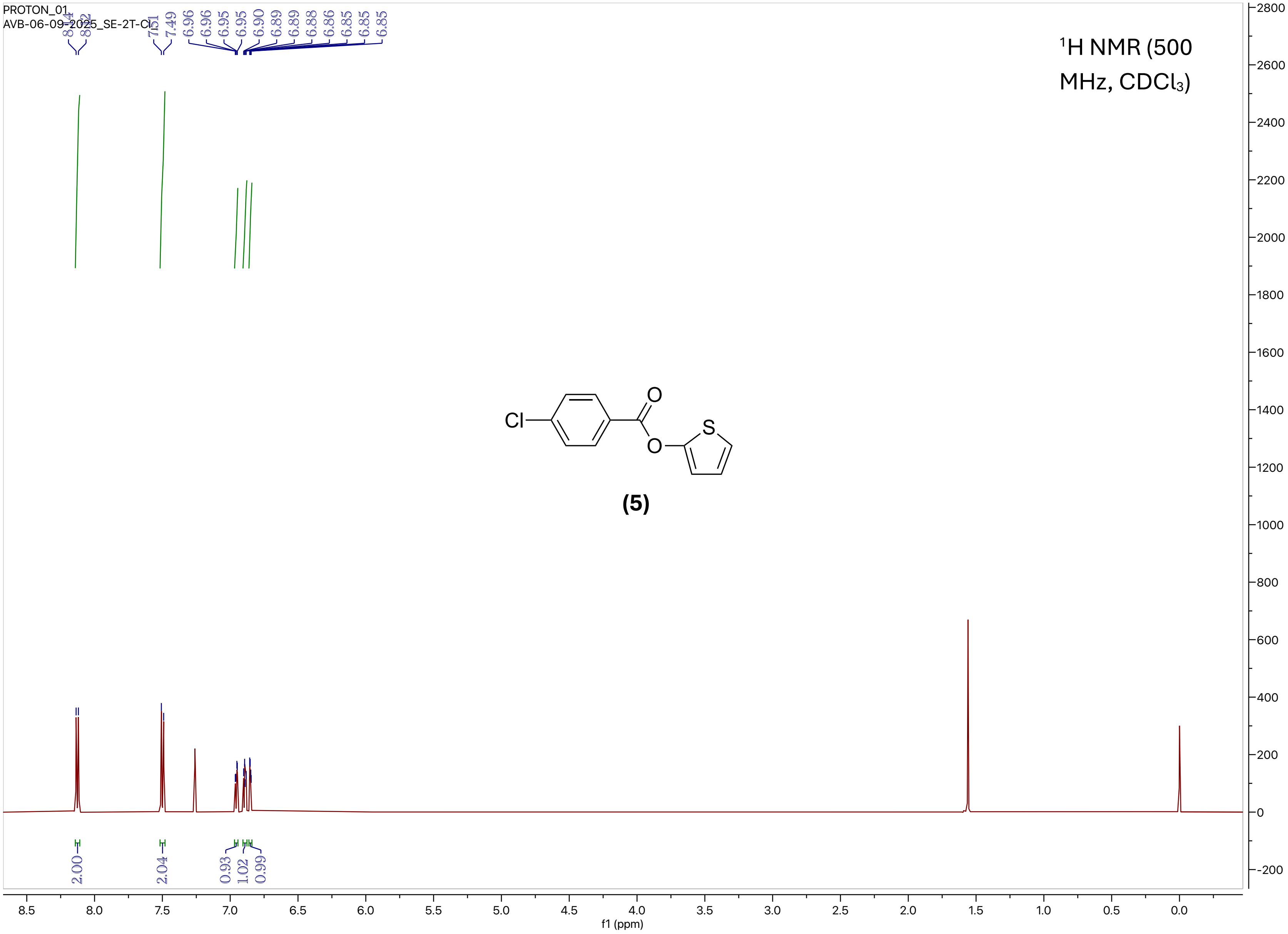
PROTON_01
AVB-06-09-2025_SE-2T-Cl
8.14
8.12
7.51
7.49
6.96
6.96
6.95
6.95
6.90
6.89
6.89
6.88
6.86
6.85
6.85
6.85
¹H NMR (500 MHz, CDCl₃)
Cl
O
O
S
(5)
2.00
2.04
0.93
1.02
0.99
f1 (ppm)

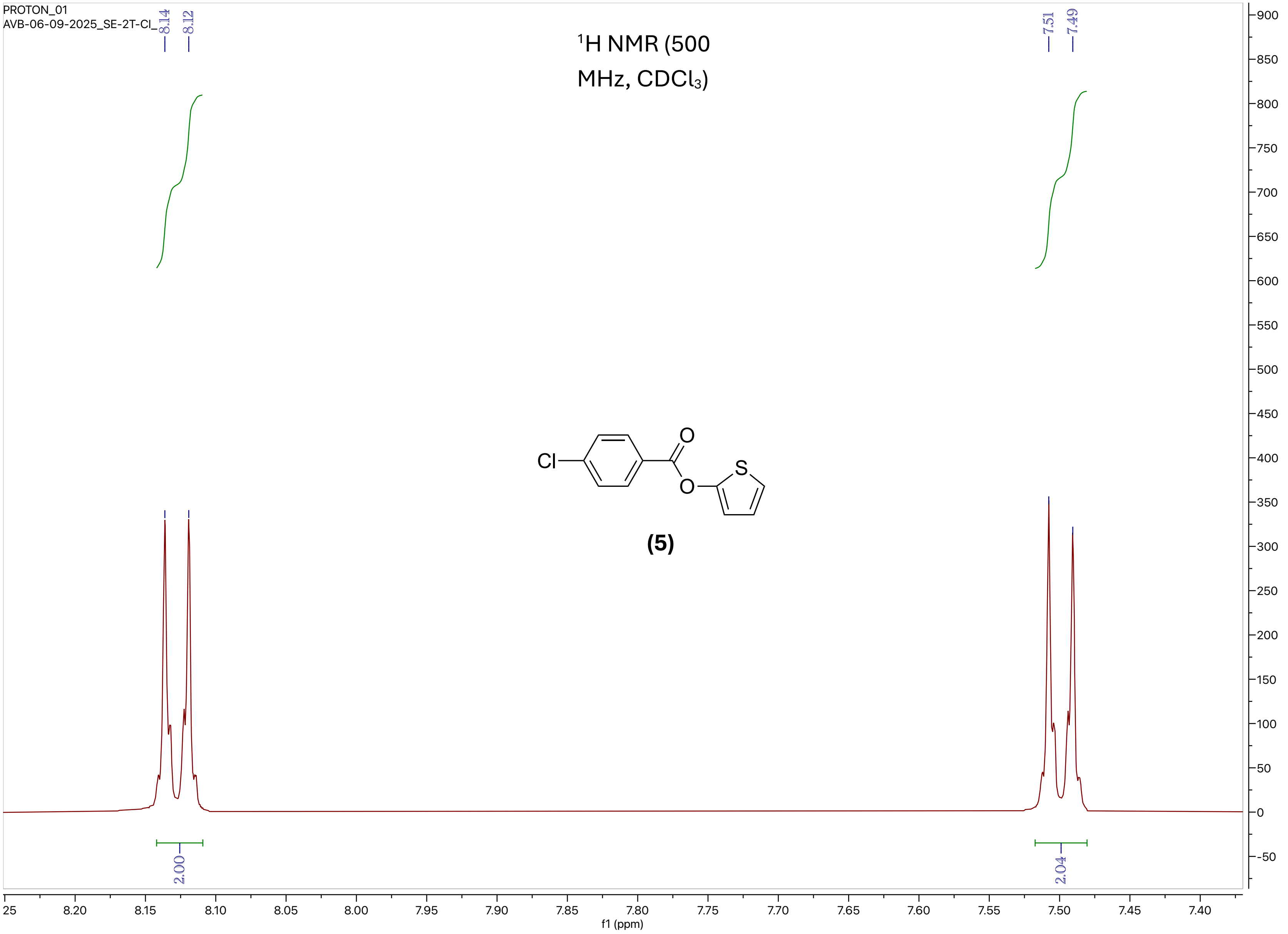

PROTON_01
AVB-06-09-2025_SE-2T-Cl_
8.14
8.12
7.51
7.49
¹H NMR (500 MHz, CDCl₃)
Cl
O
O
S
(5)
2.00
2.04
f1 (ppm)

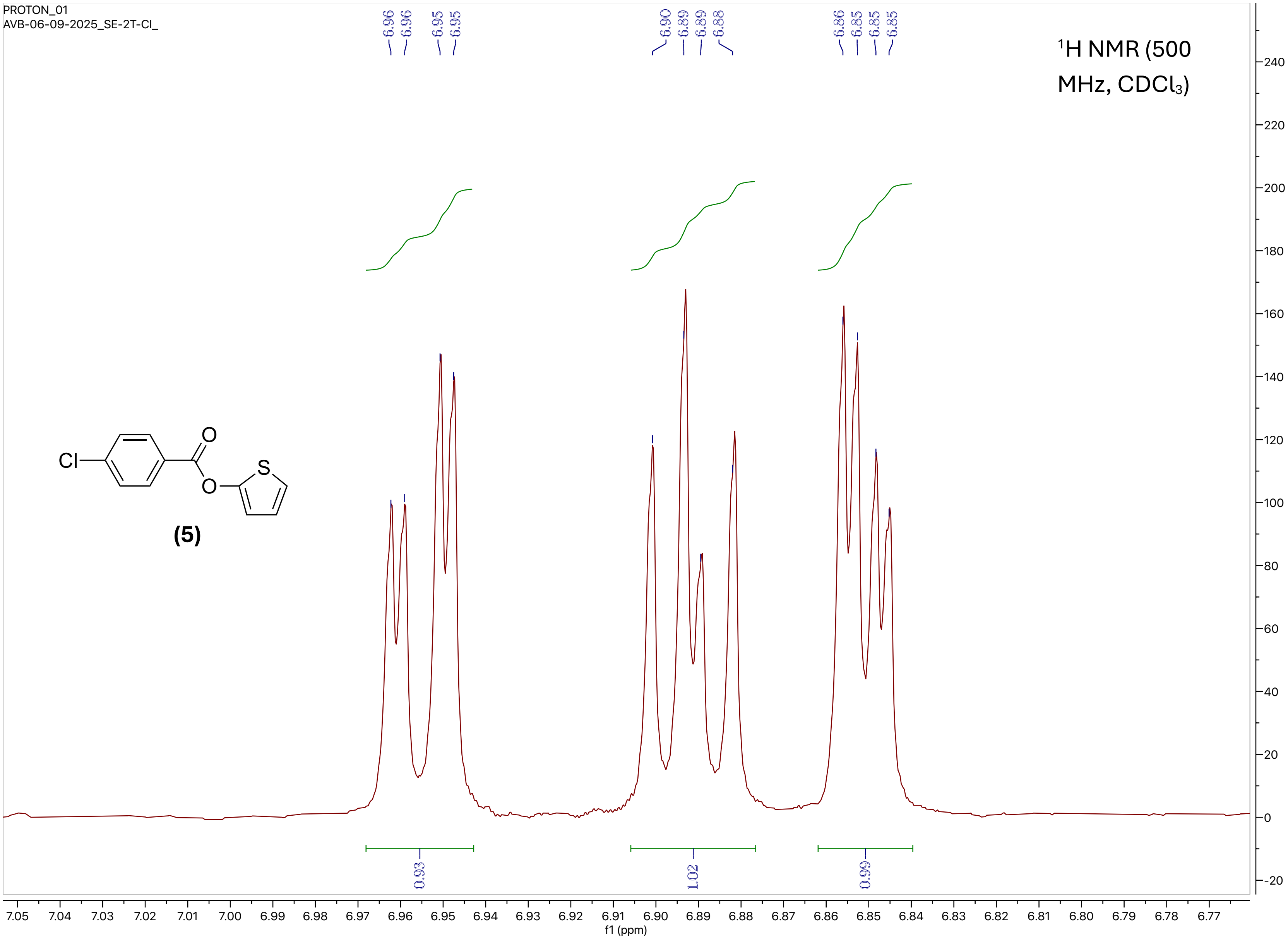
PROTON_01
AVB-06-09-2025_SE-2T-Cl_
6.96
6.96
6.95
6.95
6.90
6.89
6.89
6.88
6.86
6.85
6.85
6.85
1H NMR (500 MHz, CDCl3)
0.93
1.02
0.99
f1 (ppm)
Cl
O
O
S

(5)

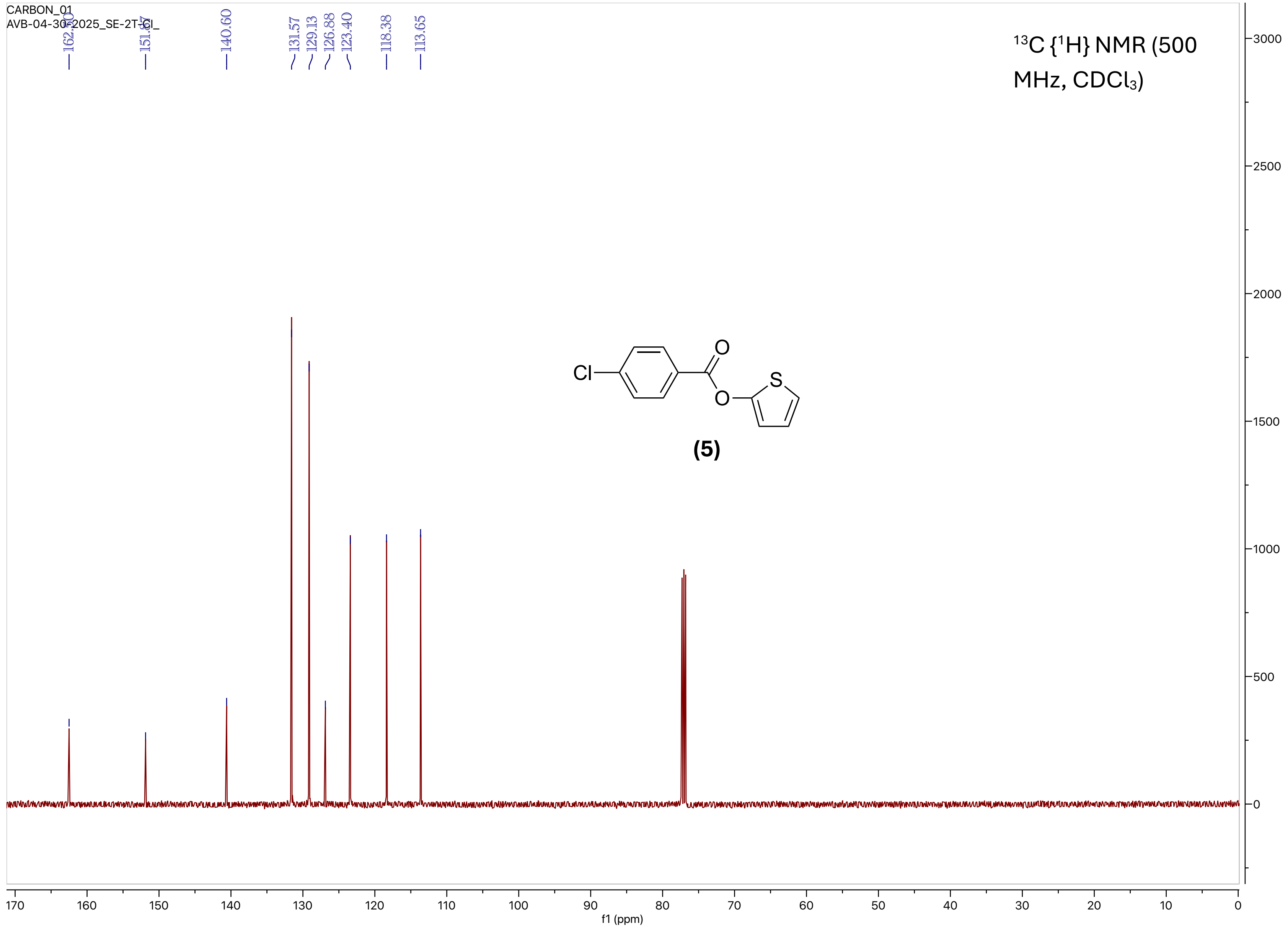

CARBON_01
AVB-04-30-2025_SE-2T-Cl_
162.40
151.67
140.60
131.57
129.13
126.88
123.40
118.38
113.65
$^{13}C\{^1H\}$ NMR (500 MHz, $CDCl_3$)
Cl
O
O
S
(5)
f1 (ppm)

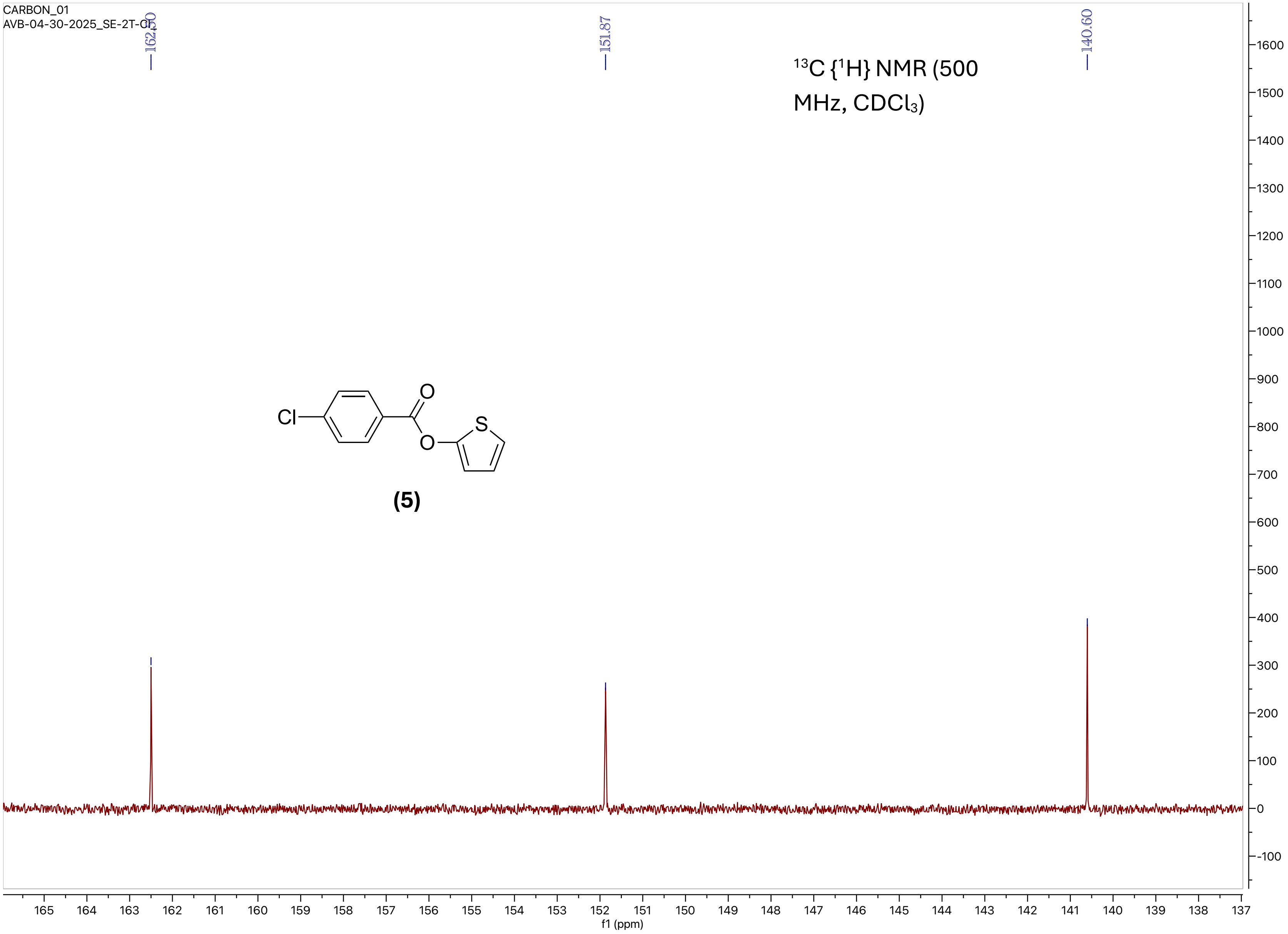
CARBON_01
AVB-04-30-2025_SE-2T-Cl
162.50
151.87
140.60
$^{13}C\{^{1}H\}$ NMR (500 MHz, $CDCl_3$)
Cl
O
O
S
(5)
1600
1500
1400
1300
1200
1100
1000
900
800
700
600
500
400
300
200
100
0
-100
165 164 163 162 161 160 159 158 157 156 155 154 153 152 151 150 149 148 147 146 145 144 143 142 141 140 139 138 137
f1 (ppm)

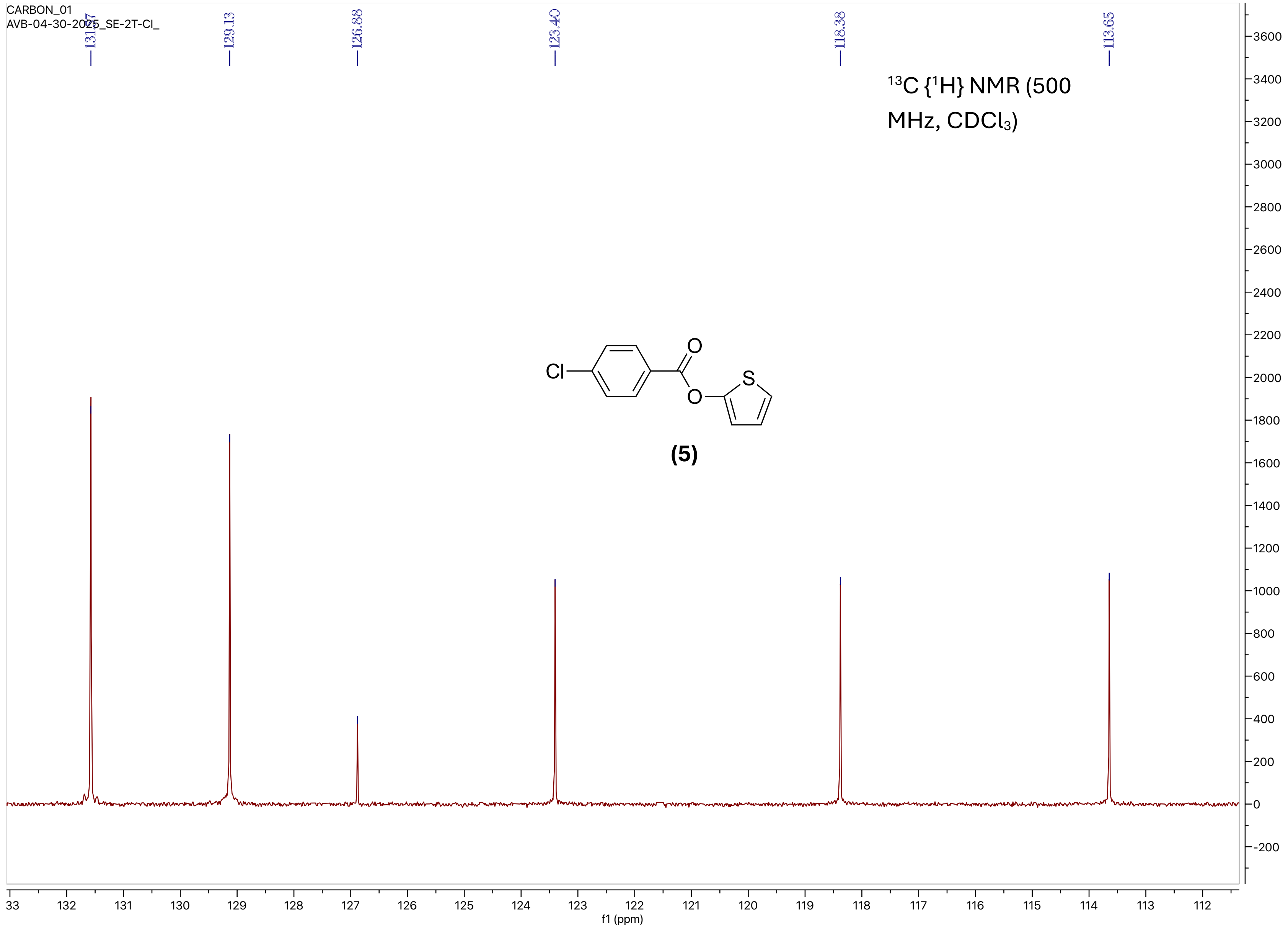
CARBON_01
AVB-04-30-2025_SE-2T-Cl_
129.13
126.88
123.40
118.38
113.65
$^{13}C\{^1H\}$ NMR (500 MHz, $CDCl_3$)
Cl
O
O
S
(5)
f1 (ppm)

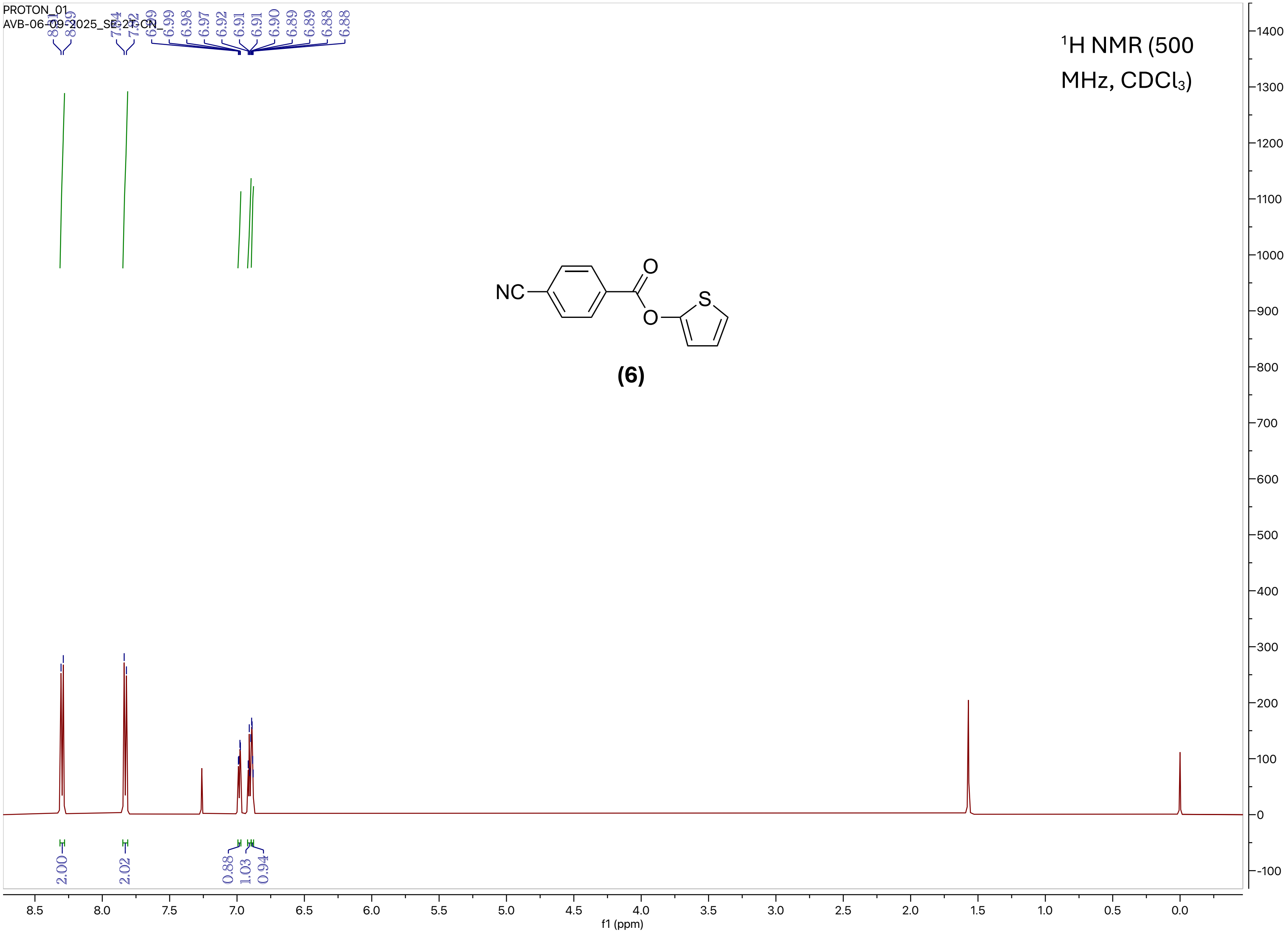
PROTON_01
AVB-06-09-2025_SE-2T-CN_
8.31
8.29
7.84
7.82
6.99
6.99
6.98
6.97
6.92
6.91
6.91
6.90
6.89
6.89
6.88
6.88
^{1}H NMR (500 MHz, $CDCl_3$)
NC
O
O
S
(6)
2.00
2.02
0.88
1.03
0.94
f1 (ppm)

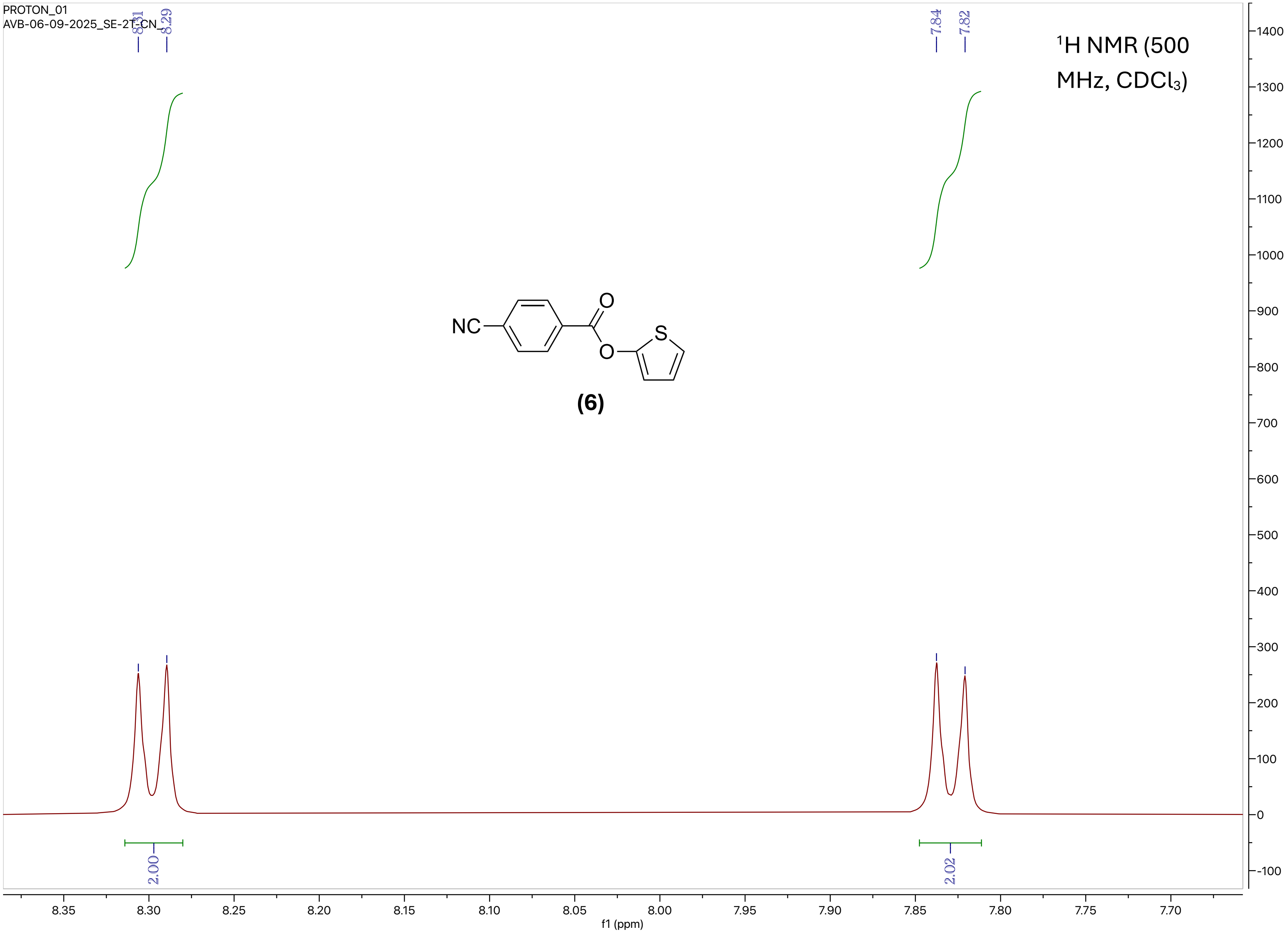
PROTON_01
AVB-06-09-2025_SE-2T-CN_1
8.31
8.29
7.84
7.82
1H NMR (500 MHz, CDCl3)
NC
O
O
S
(6)
2.00
2.02
f1 (ppm)

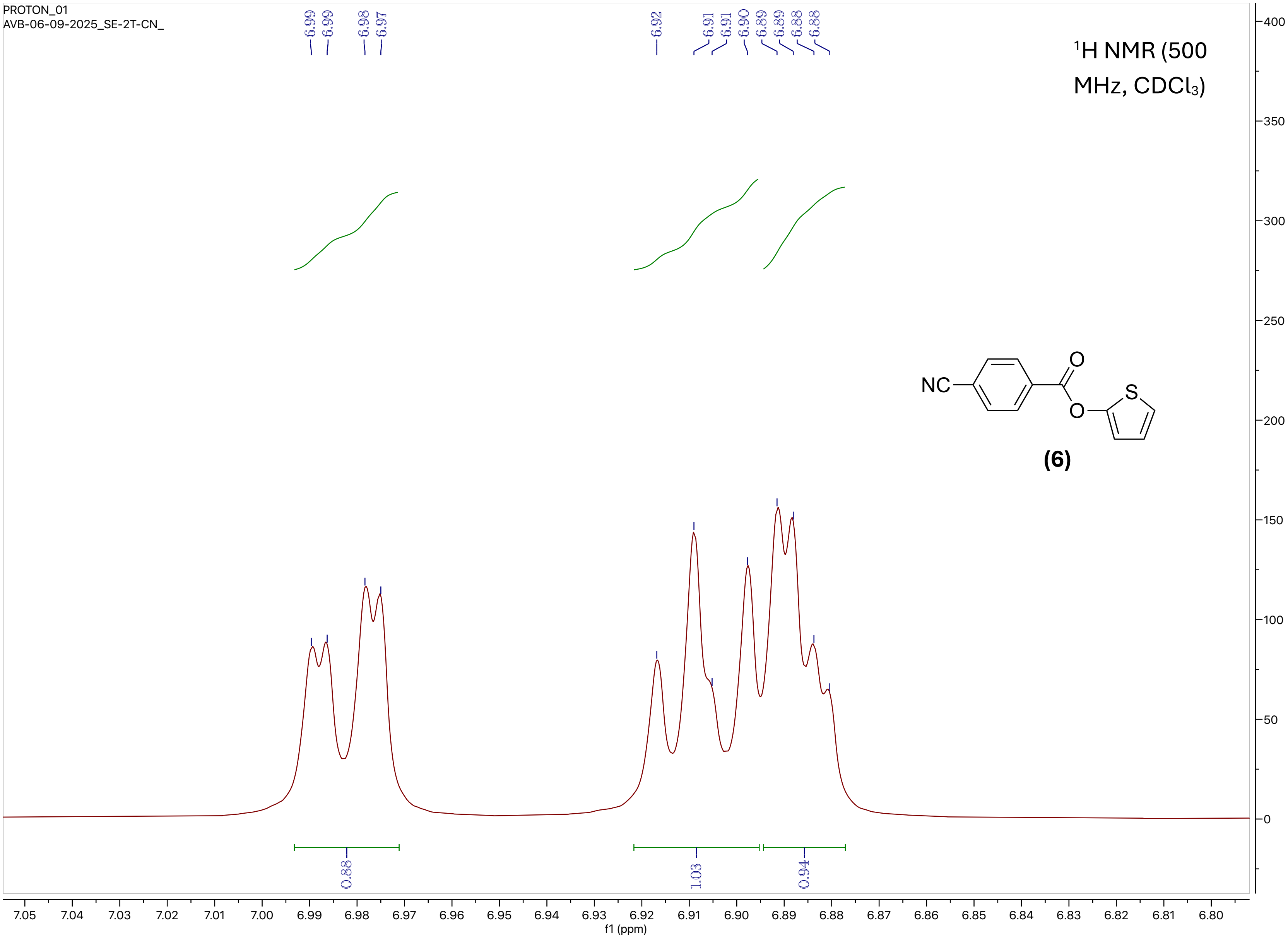
PROTON_01
AVB-06-09-2025_SE-2T-CN_
6.99
6.99
6.98
6.97
6.92
6.91
6.91
6.90
6.89
6.89
6.88
6.88
1H NMR (500 MHz, CDCl3)
NC
O
O
S
(6)
0.88
1.03
0.94
f1 (ppm)

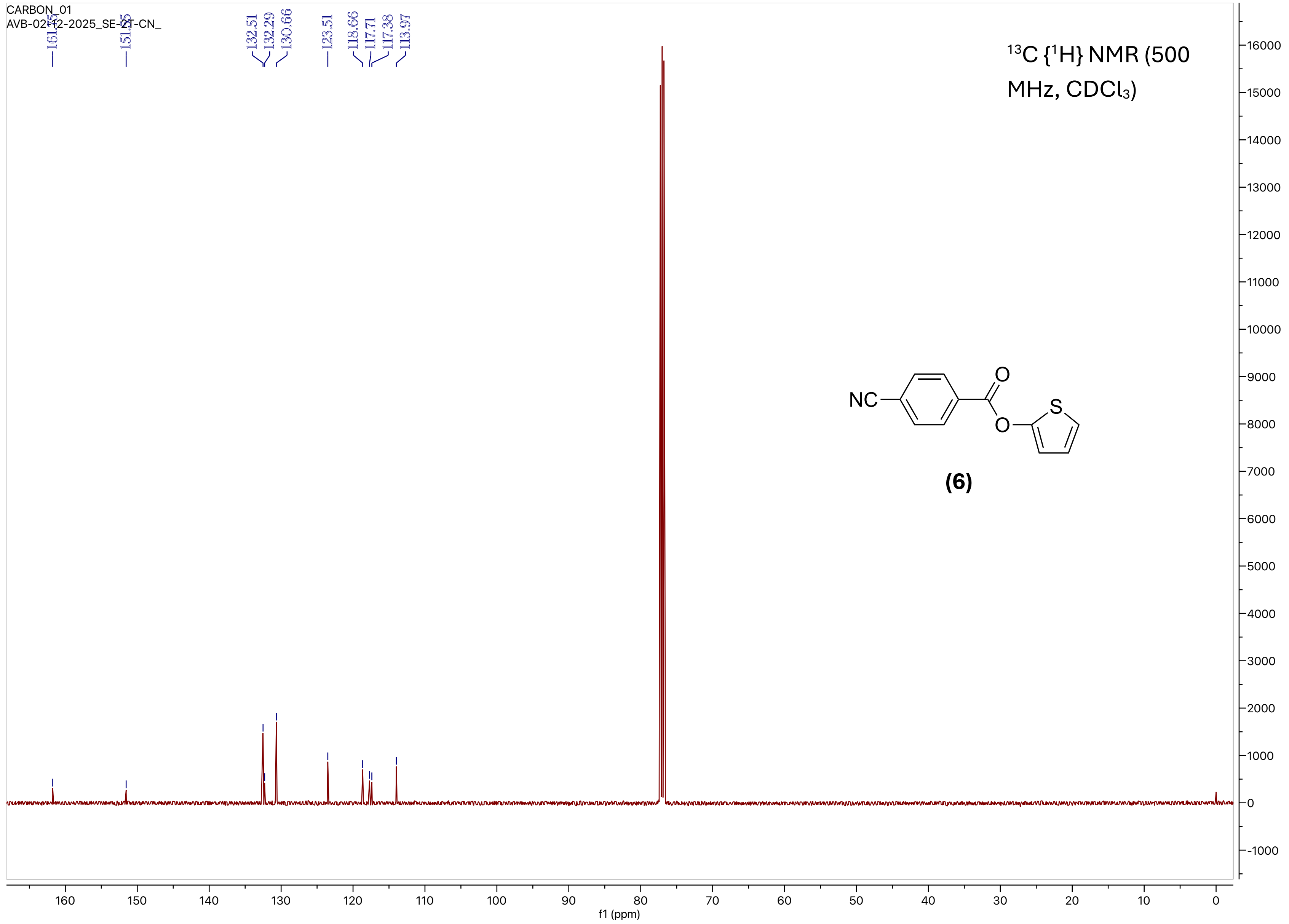
CARBON_01
AVB-02-12-2025_SE-2T-CN_
161.55
151.55
132.51
132.29
130.66
123.51
118.66
117.71
117.38
113.97
$^{13}C\{^1H\}$ NMR (500 MHz, $CDCl_3$)
NC
O
O
S
(6)
f1 (ppm)

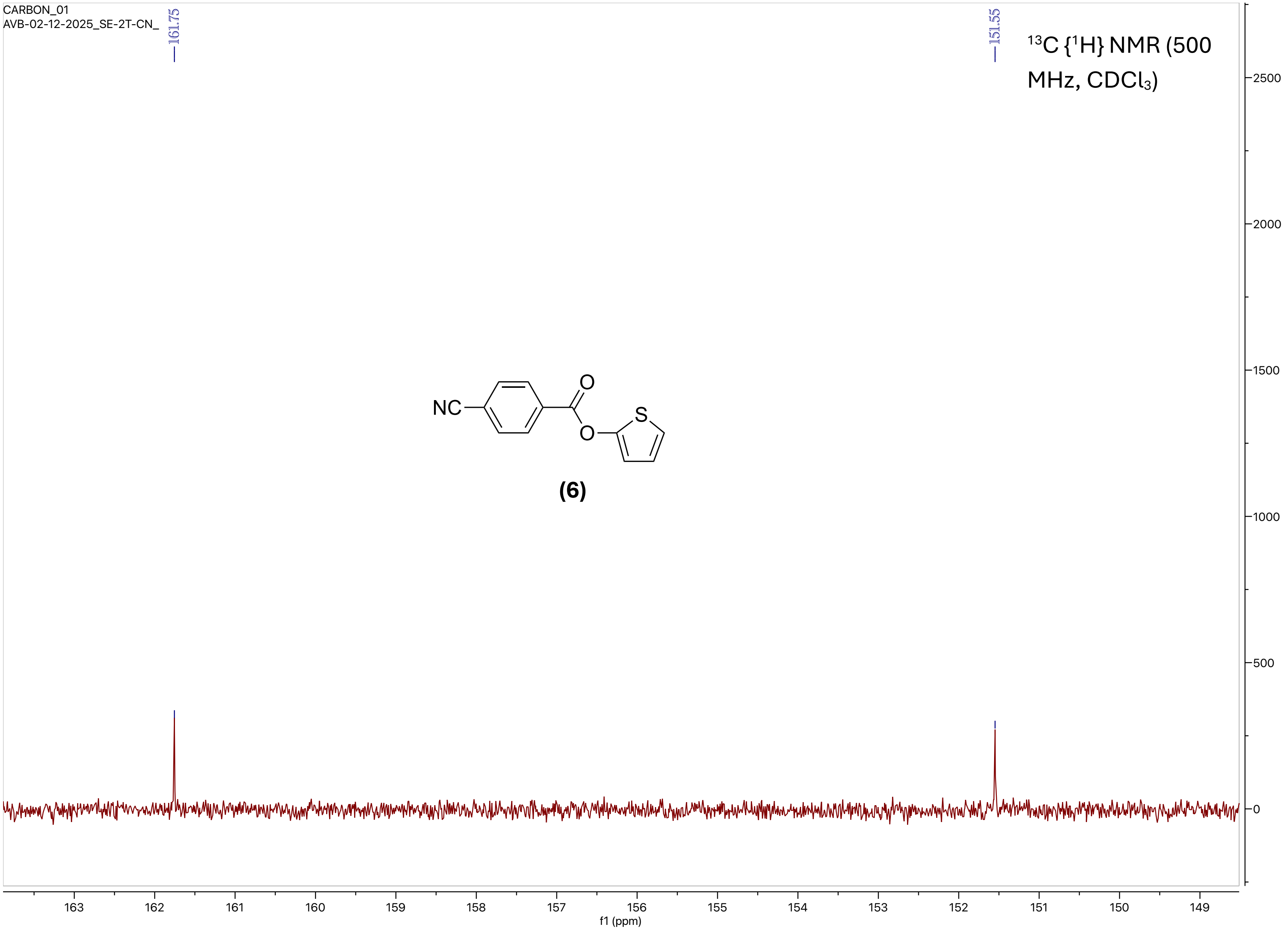
CARBON_01
AVB-02-12-2025_SE-2T-CN_
161.75
151.55
$^{13}C\{^1H\}$ NMR (500 MHz, $CDCl_3$)
NC
O
O
S
(6)
2500
2000
1500
1000
500
0
163
162
161
160
159
158
157
156
155
154
153
152
151
150
149
f1 (ppm)

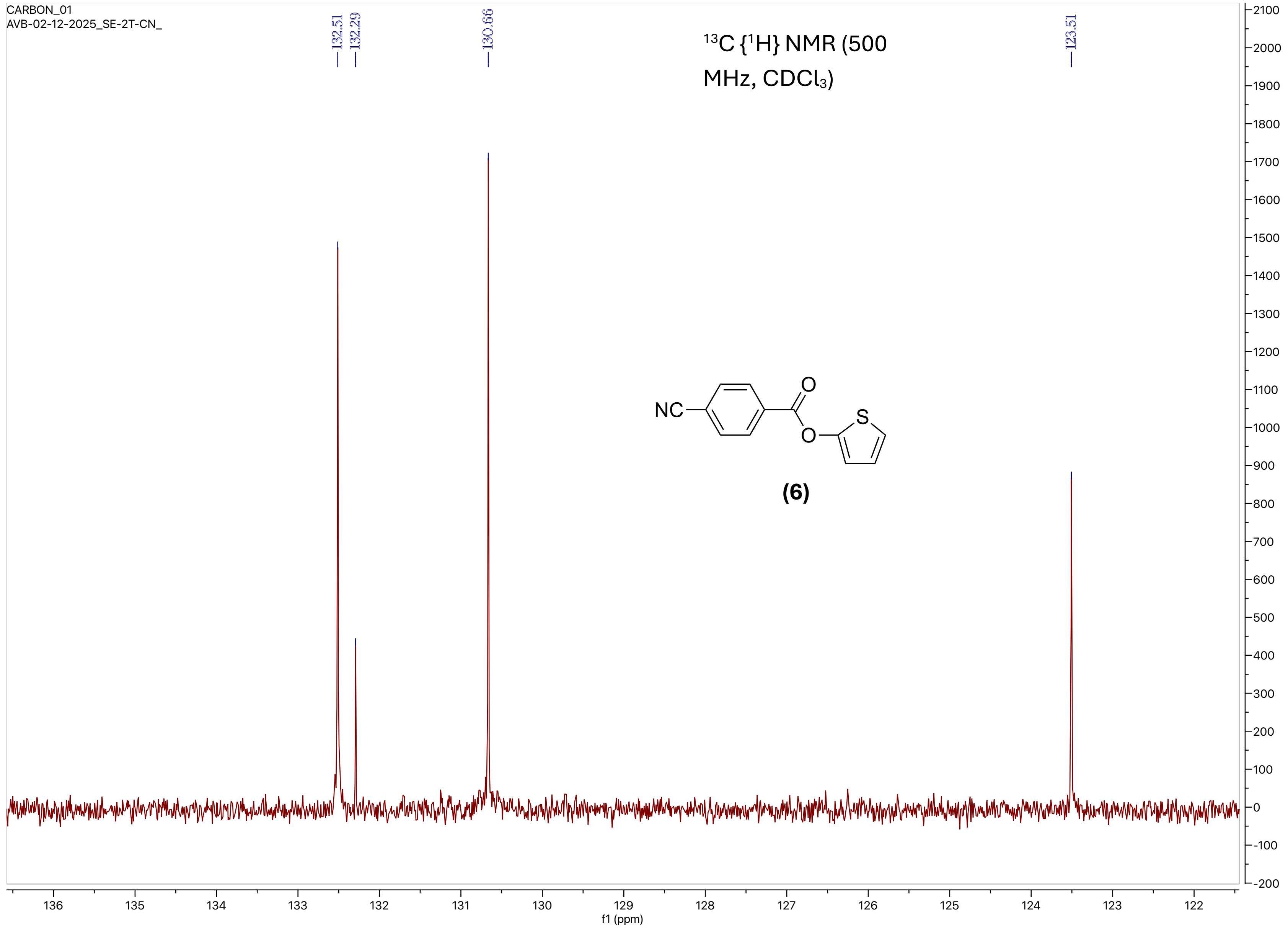
CARBON_01
AVB-02-12-2025_SE-2T-CN_
132.51
132.29
130.66
123.51
$^{13}C\{^1H\}$ NMR (500 MHz, $CDCl_3$)
NC
O
O
S
(6)
f1 (ppm)

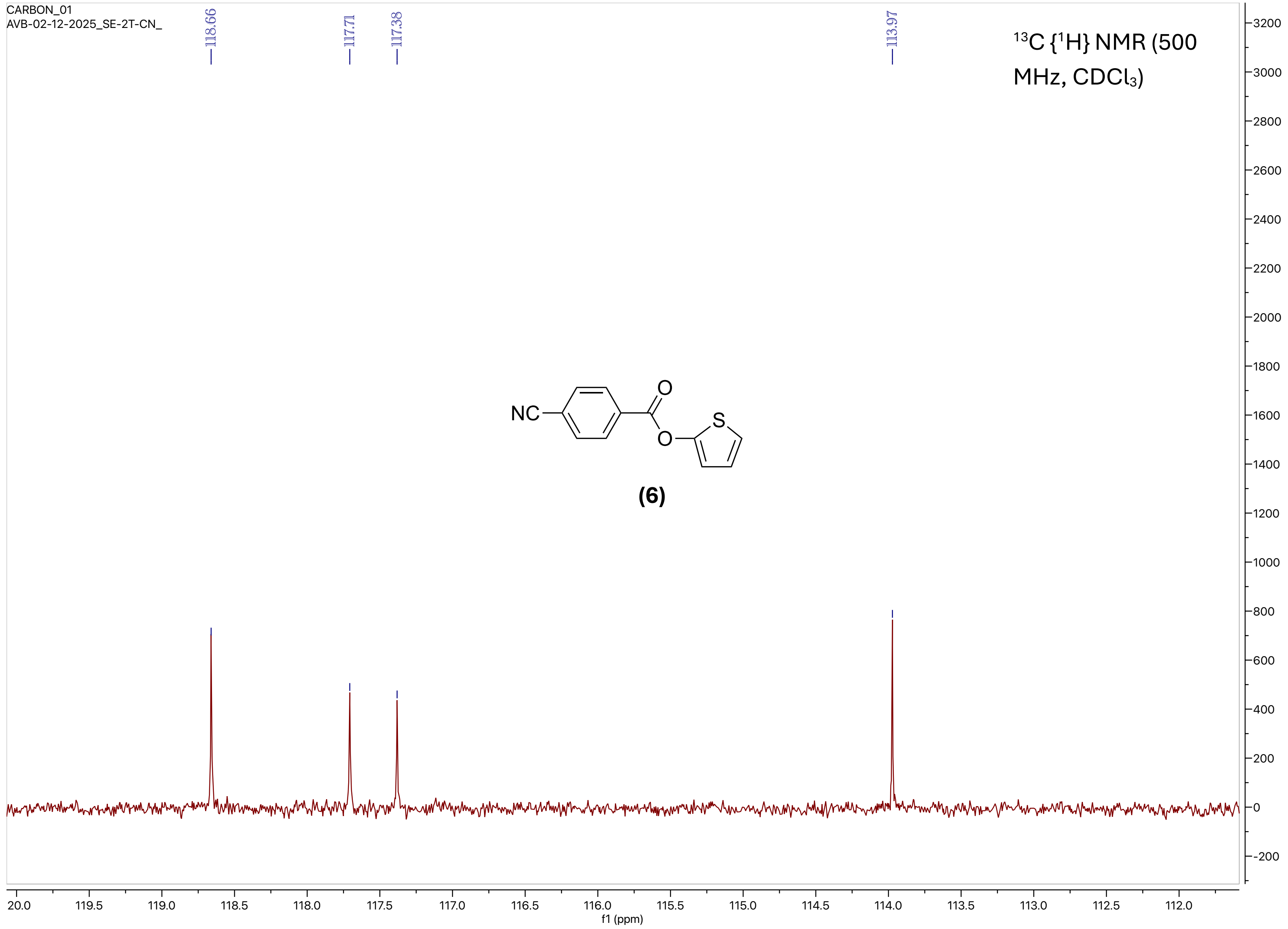

CARBON_01
AVB-02-12-2025_SE-2T-CN_
118.66
117.71
117.38
113.97
^{13}C {^{1}H} NMR (500 MHz, CDCl$_3$)
NC
O
O
S
(6)
f1 (ppm)

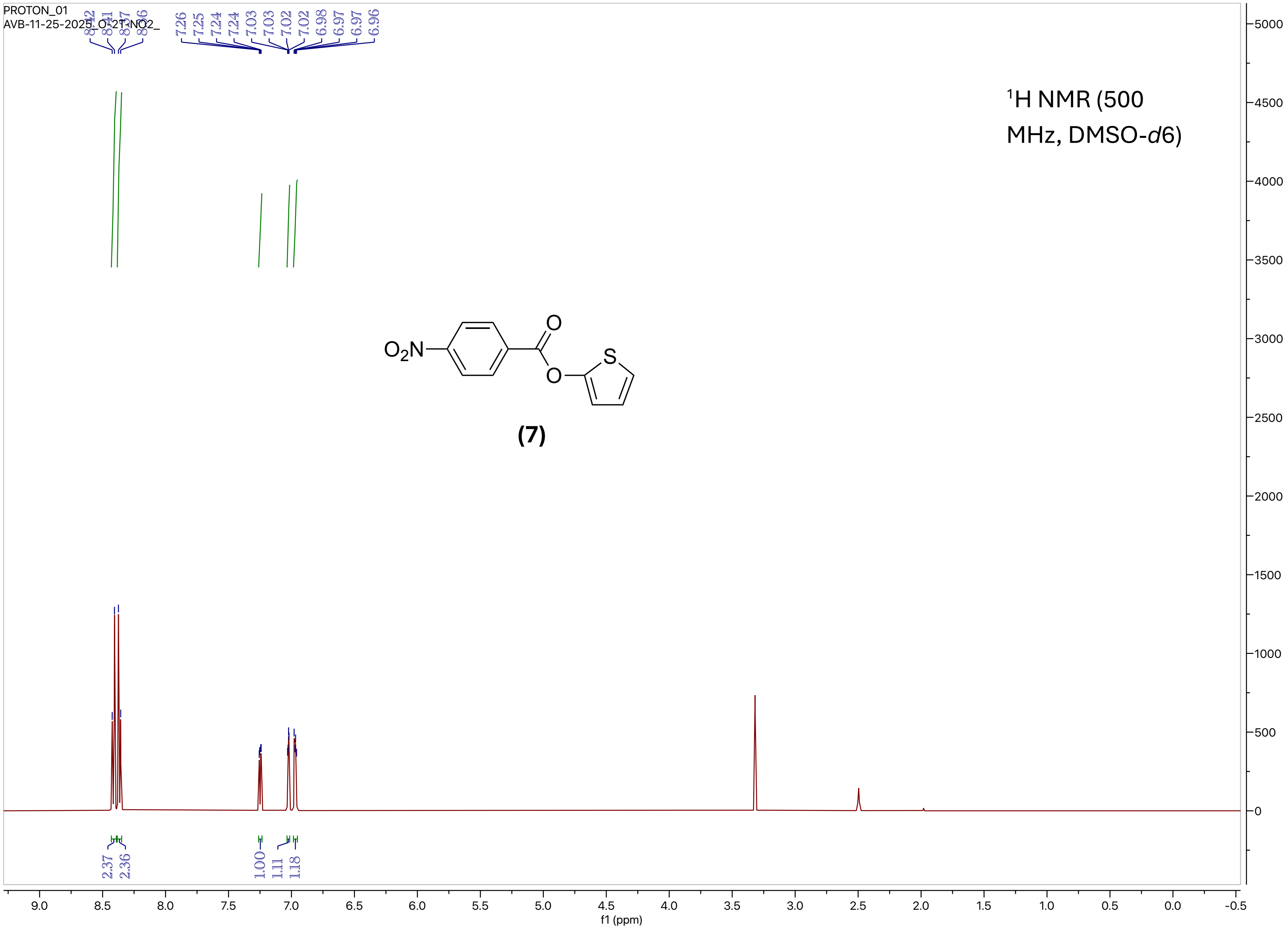
PROTON_01
AVB-11-25-2025_O-2T-NO2_
8.42
8.41
8.37
8.36
7.26
7.25
7.24
7.24
7.03
7.03
7.02
7.02
6.98
6.97
6.97
6.96
1H NMR (500 MHz, DMSO-d6)
O2N
O
O
S
(7)
2.37
2.36
1.00
1.11
1.18
f1 (ppm)

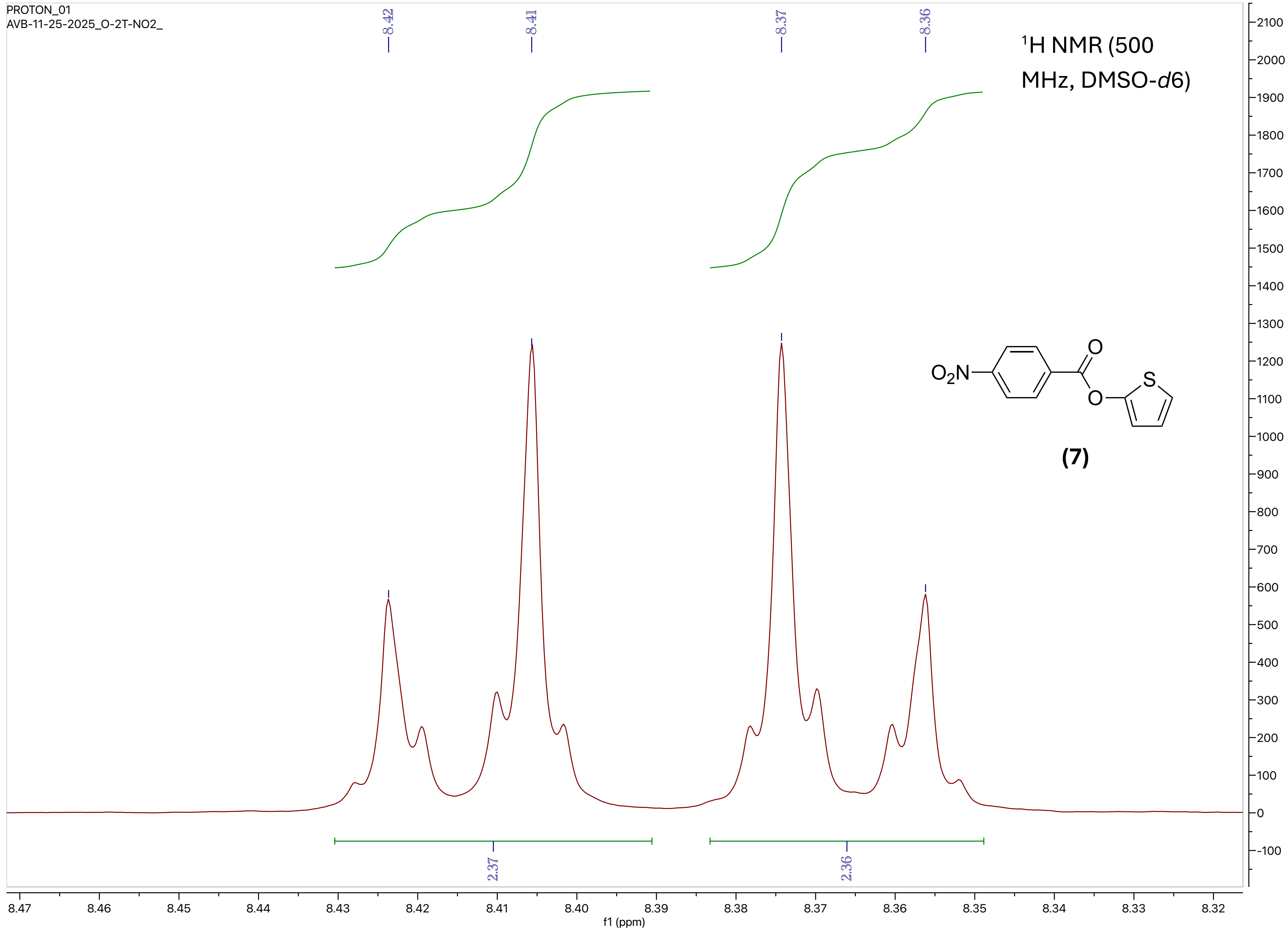
PROTON_01
AVB-11-25-2025_O-2T-NO2_
8.42
8.41
8.37
8.36
^{1}H NMR (500 MHz, DMSO-*d6*)
O
O_2N
S
O
(7)
2.37
2.36
f1 (ppm)

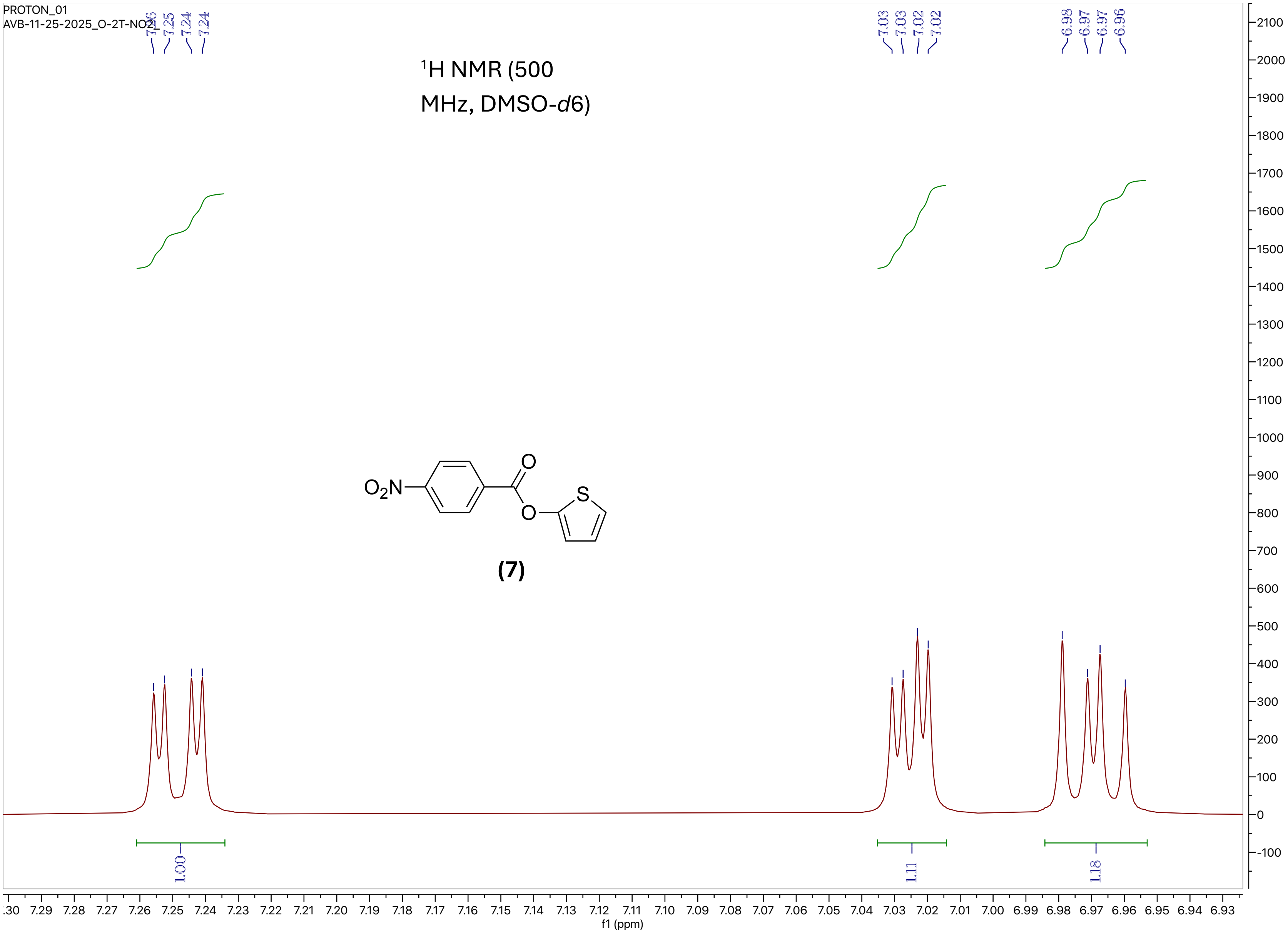
PROTON_01
AVB-11-25-2025_O-2T-NO2
7.26
7.25
7.24
7.24
7.03
7.03
7.02
7.02
6.98
6.97
6.97
6.96
¹H NMR (500 MHz, DMSO-d6)
O2N
O
O
S
(7)
1.00
1.11
1.18
f1 (ppm)

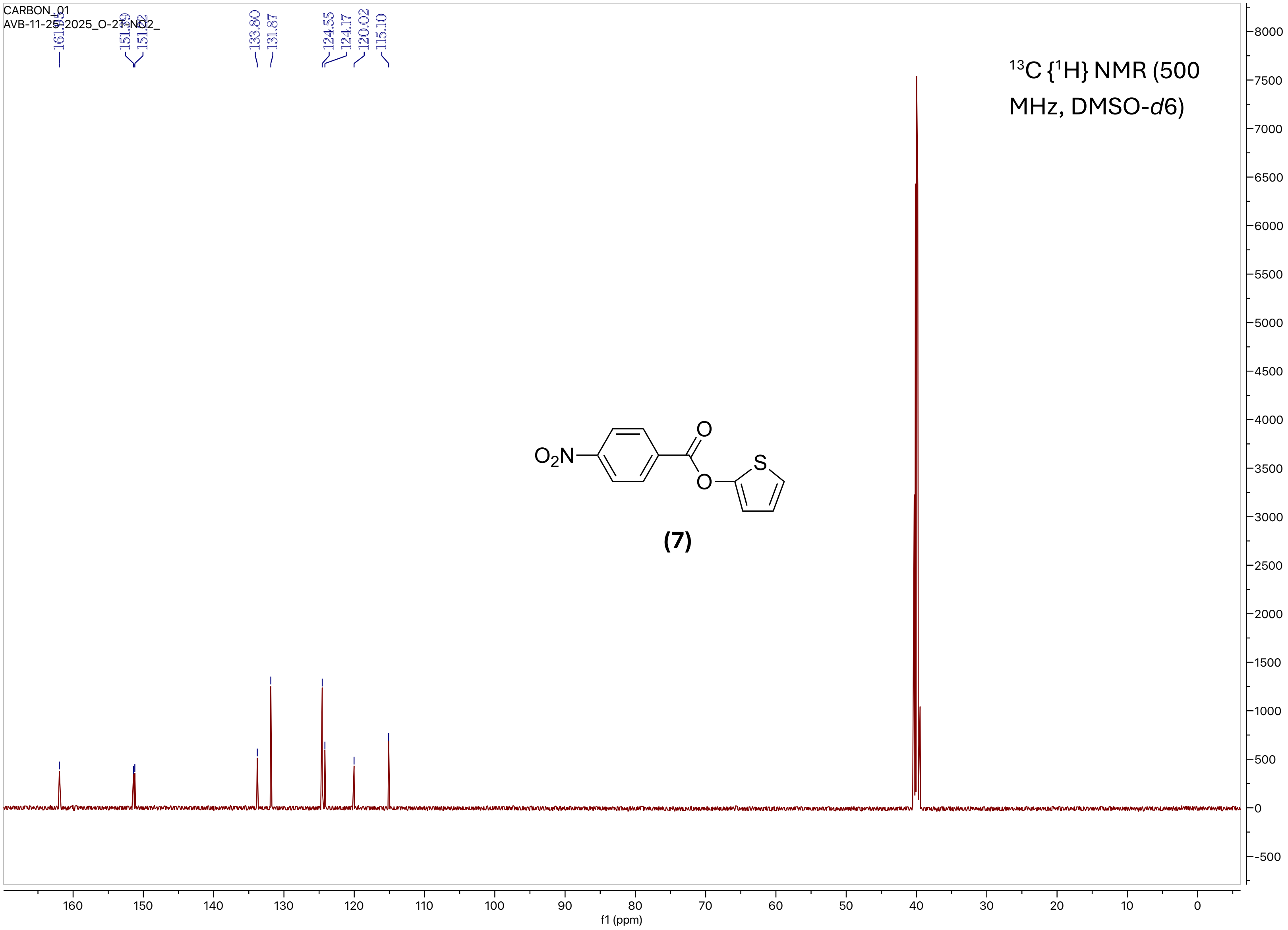
CARBON_01
AVB-11-25-2025_O-2T-NO2_
161.95
151.39
151.32
133.80
131.87
124.55
124.17
120.02
115.10
13C {1H} NMR (500 MHz, DMSO-d6)
O2N
O
O
S
(7)
f1 (ppm)

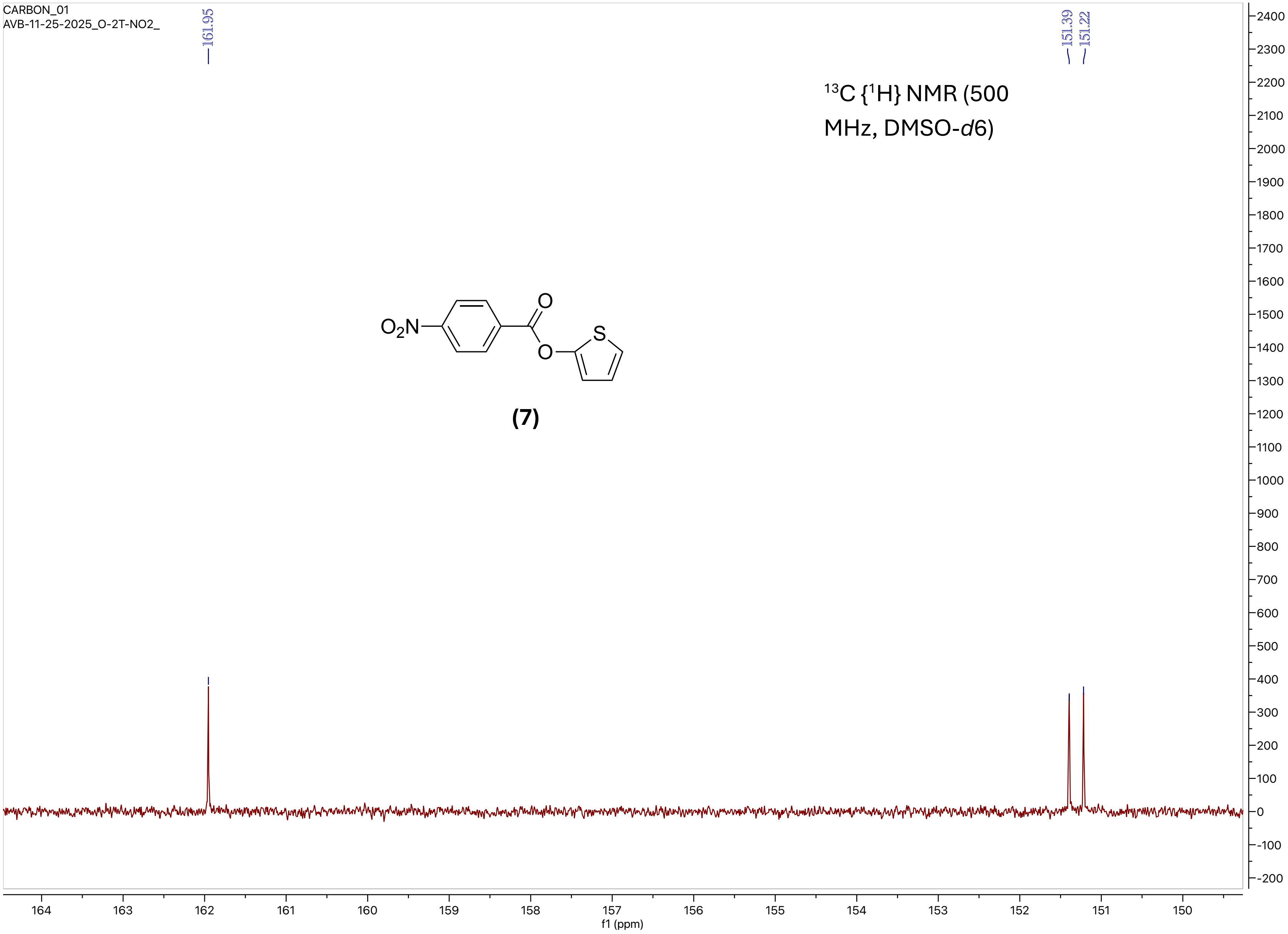
CARBON_01
AVB-11-25-2025_O-2T-NO2_
161.95
151.39
151.22
$^{13}C\{^1H\}$ NMR (500 MHz, DMSO-*d*6)
O_2N
O
O
S
(7)
f1 (ppm)

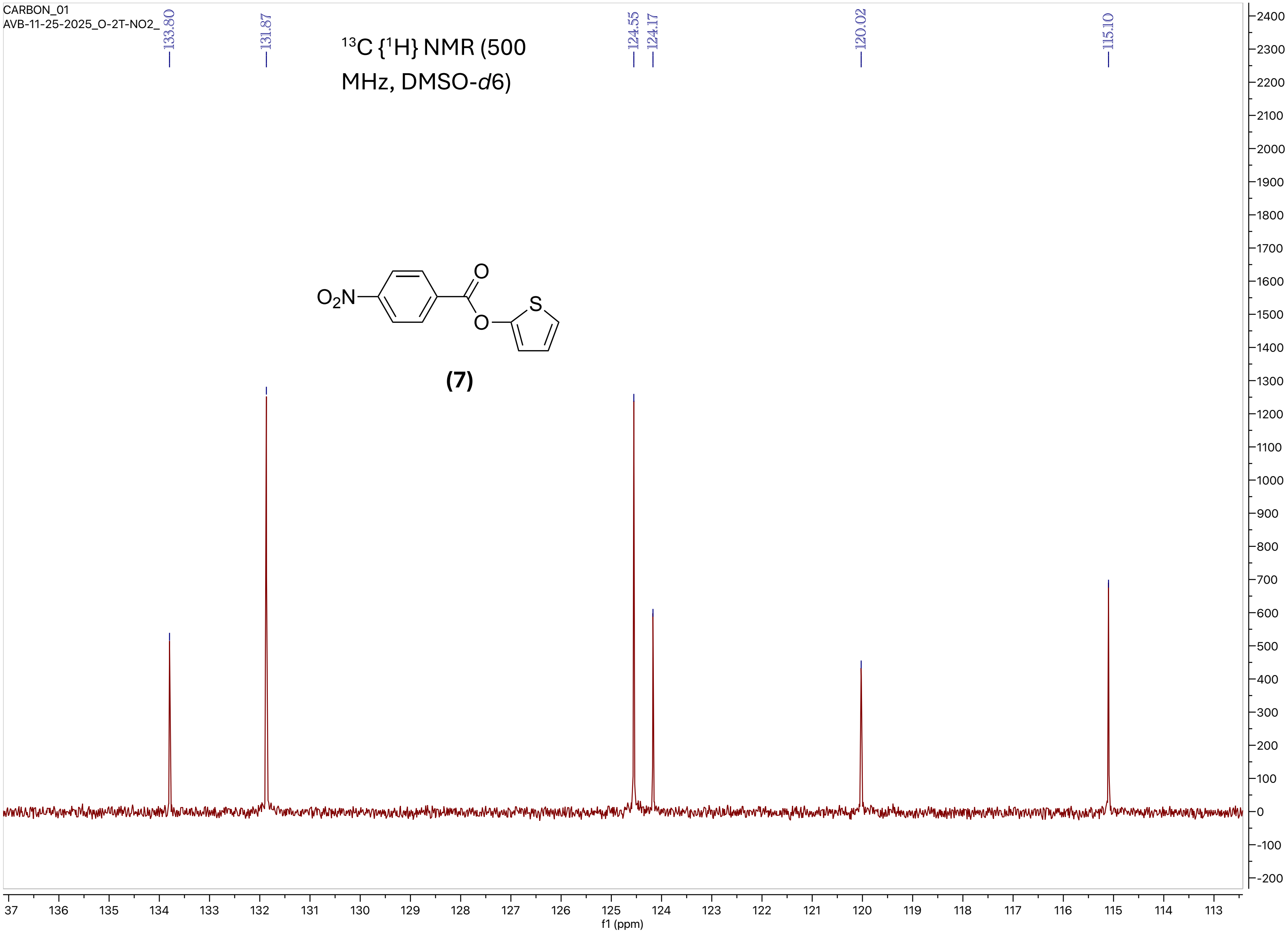

CARBON_01
AVB-11-25-2025_O-2T-NO2_
133.80
131.87
124.55
124.17
120.02
115.10
$^{13}C\ \{^{1}H\}$ NMR (500 MHz, DMSO-*d6*)
O_2N
O
O
S
(7)
f1 (ppm)

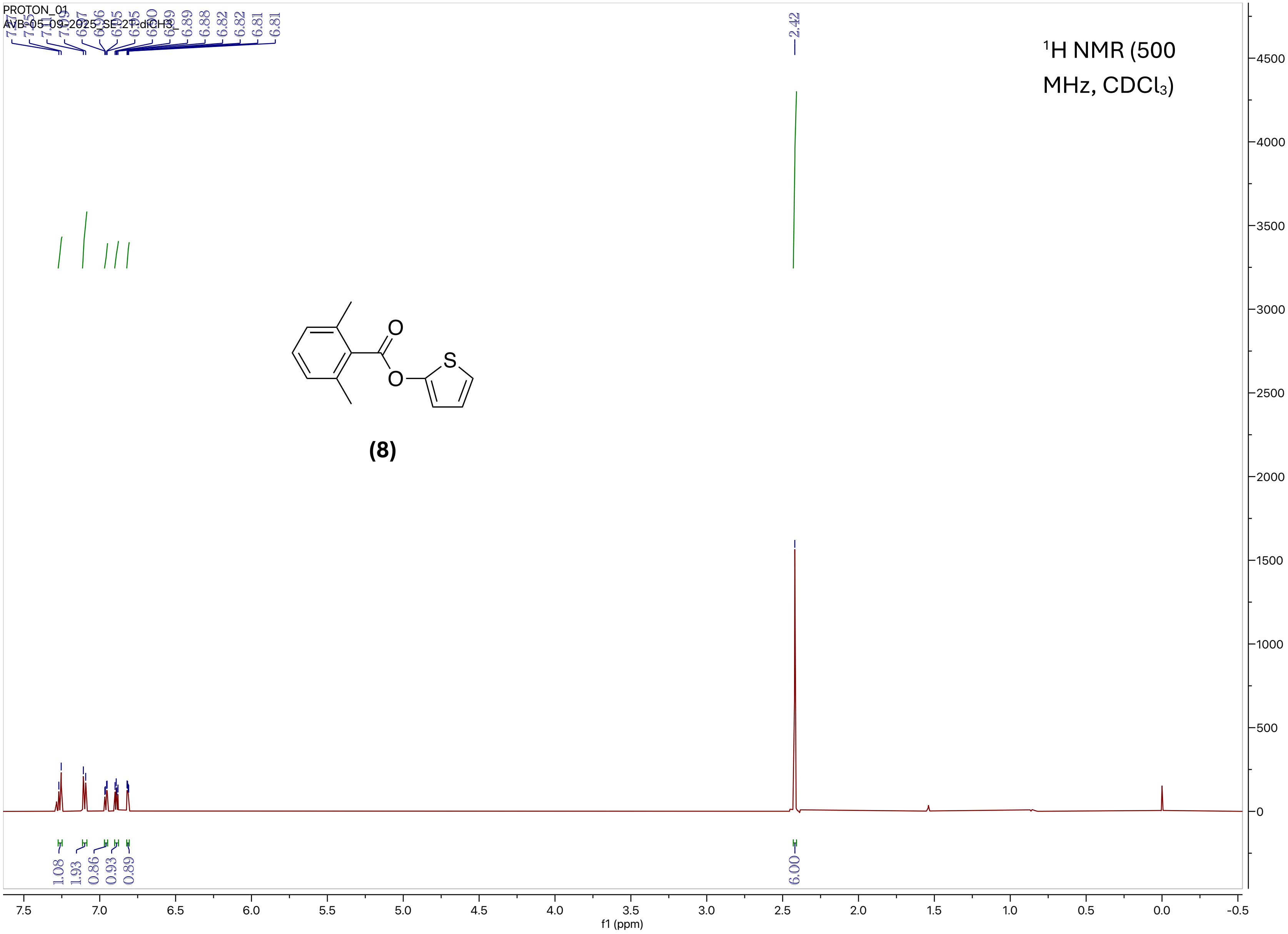
PROTON_01
AVB-05-09-2025_SE-2T-diCH3_
6.96
6.95
6.89
6.88
6.82
6.82
6.81
6.81
2.42
1H NMR (500 MHz, CDCl3)
O
S
O
(8)
1.08
1.93
0.86
0.93
0.89
6.00
f1 (ppm)

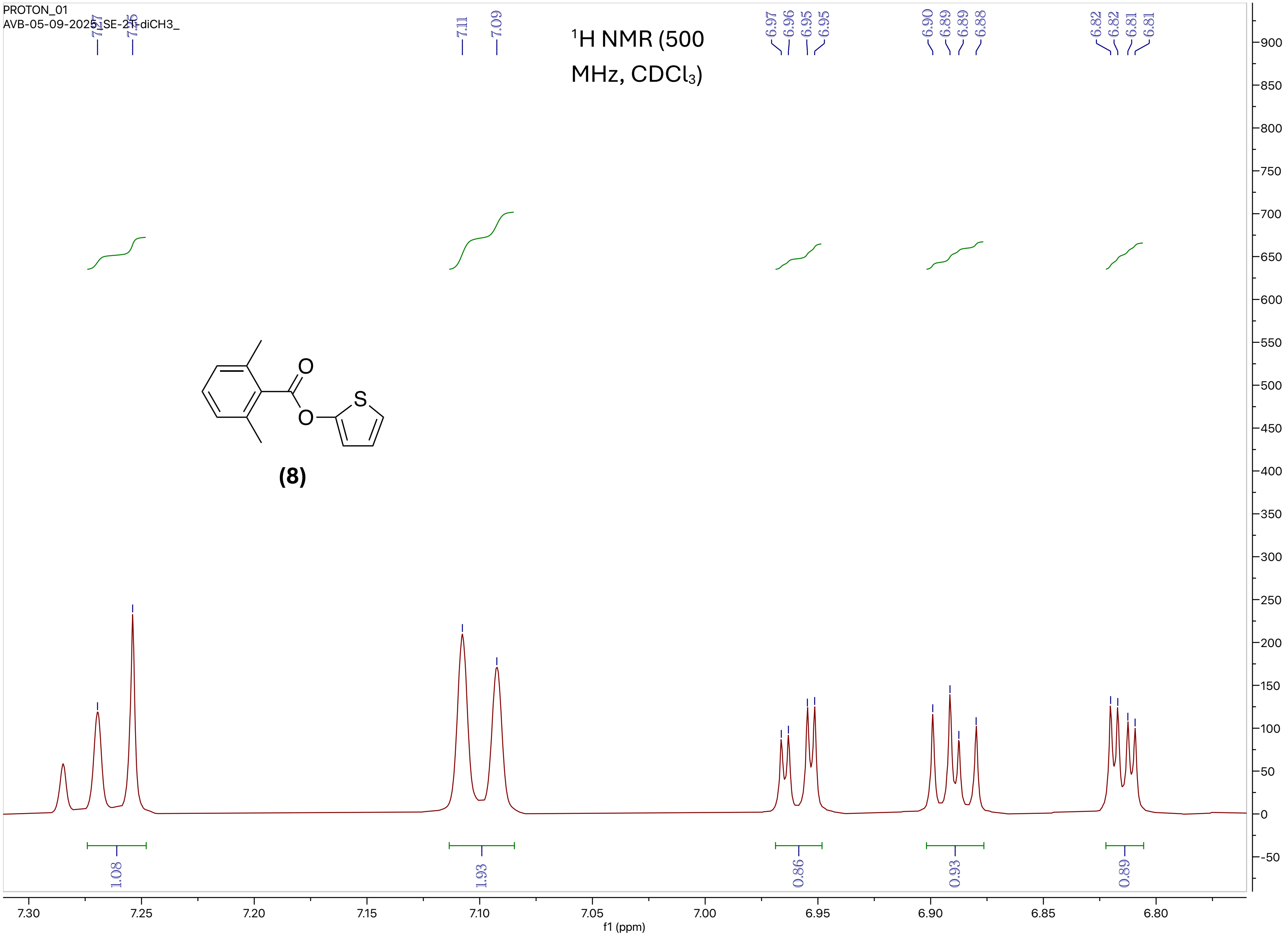
PROTON_01
AVB-05-09-2025_SE-2T-diCH3_
1H NMR (500 MHz, CDCl3)
(8)
7.27
7.25
7.11
7.09
6.97
6.96
6.95
6.95
6.90
6.89
6.89
6.88
6.82
6.82
6.81
6.81
1.08
1.93
0.86
0.93
0.89
f1 (ppm)

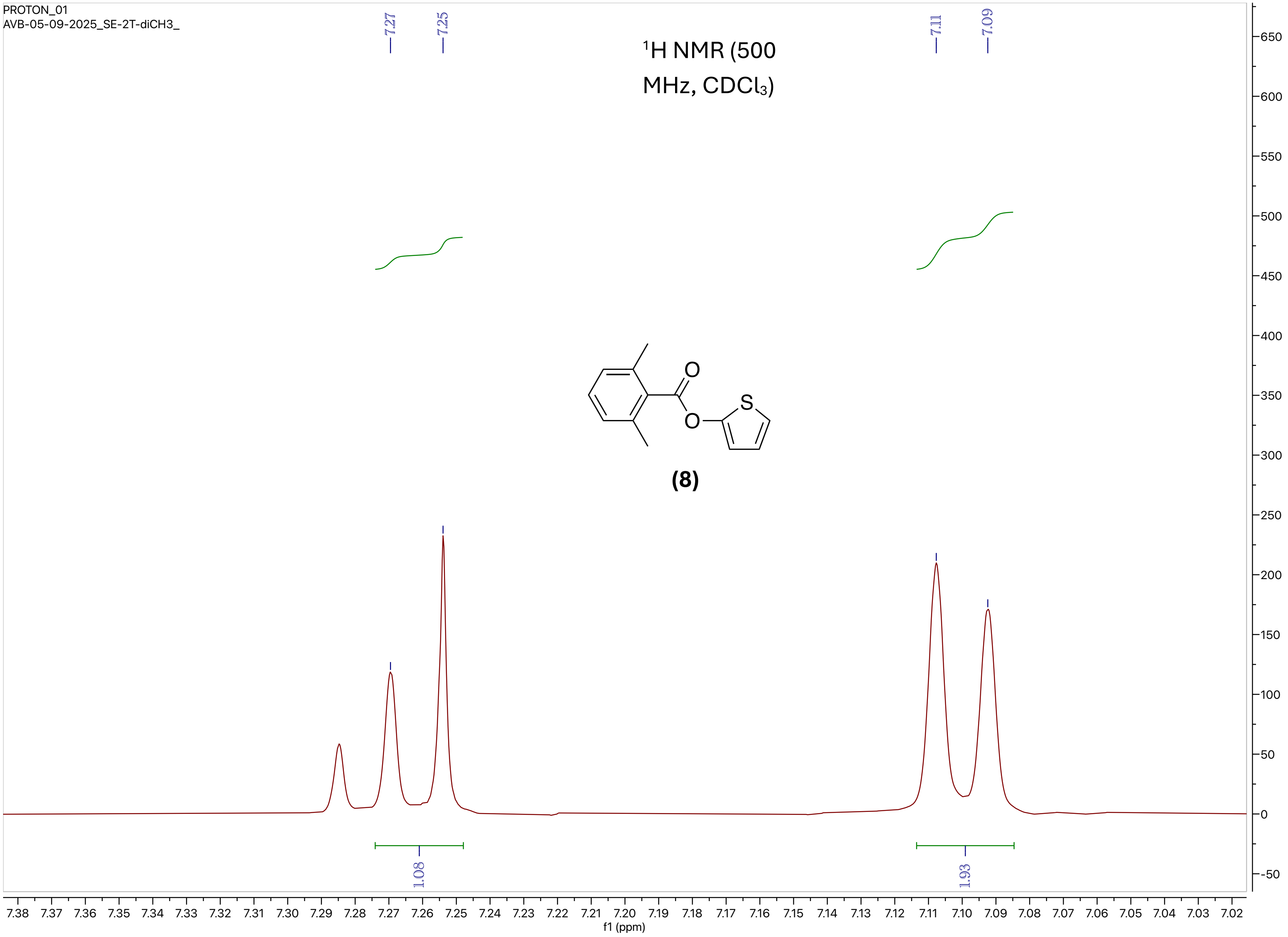
PROTON_01
AVB-05-09-2025_SE-2T-diCH3_
7.27
7.25
7.11
7.09
^{1}H NMR (500 MHz, $CDCl_3$)
1.08
1.93
O
O
S
(8)
f1 (ppm)

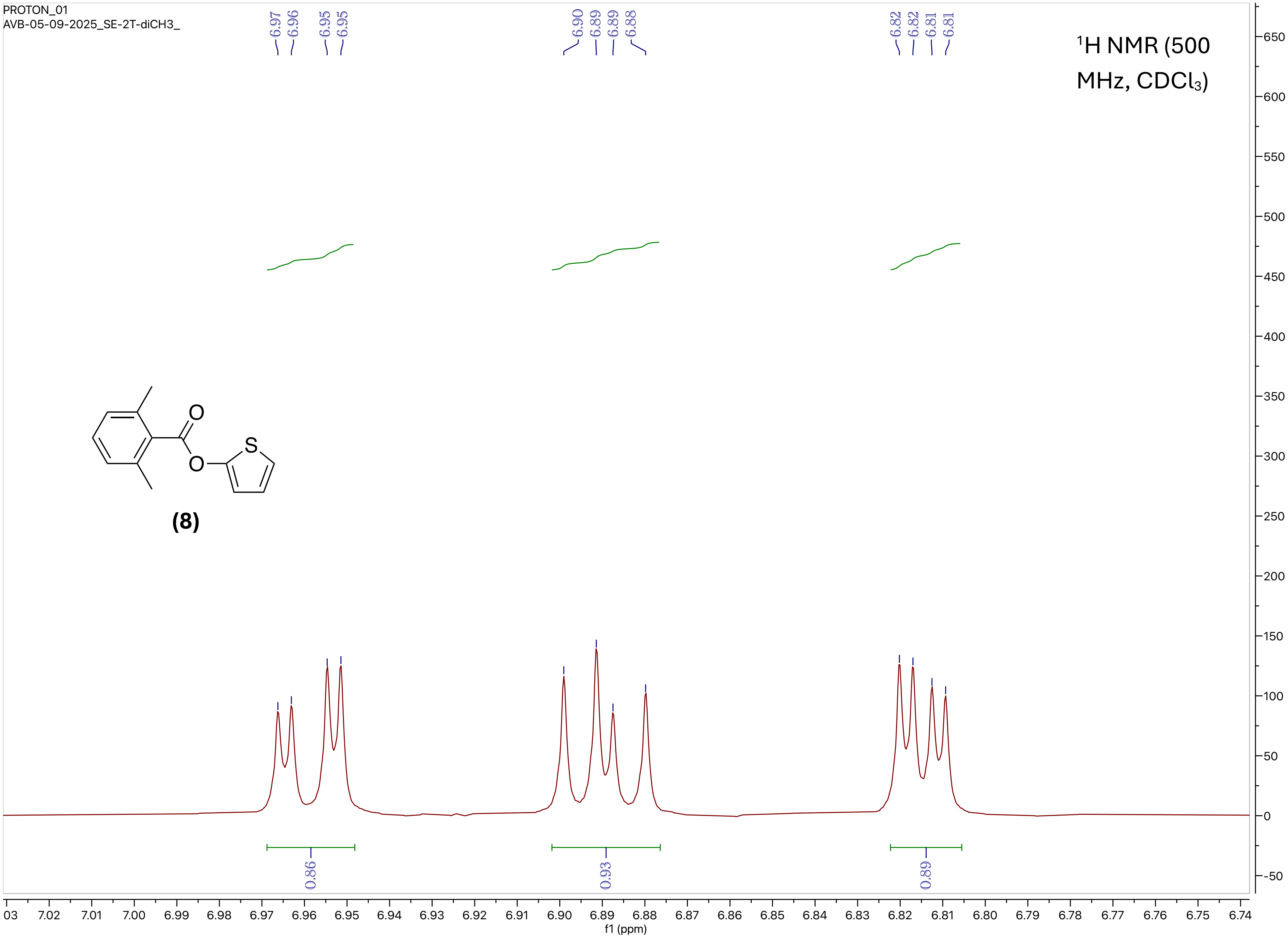

PROTON_01
AVB-05-09-2025_SE-2T-diCH3_
6.97
6.96
6.95
6.95
6.90
6.89
6.89
6.88
6.82
6.82
6.81
6.81
¹H NMR (500 MHz, CDCl₃)
O
O
S
(8)
0.86
0.93
0.89
f1 (ppm)

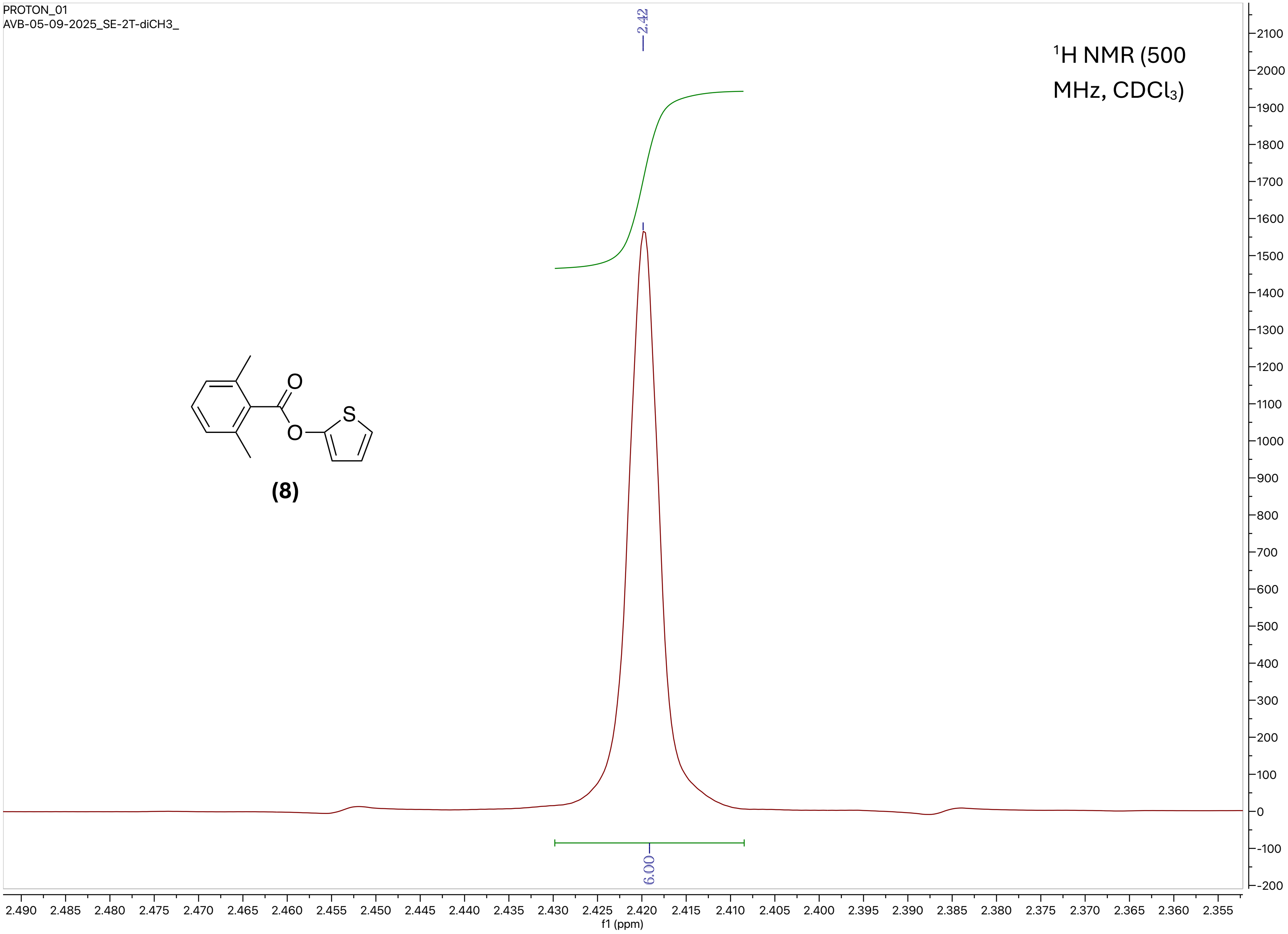
PROTON_01
AVB-05-09-2025_SE-2T-diCH3_
2.42
¹H NMR (500 MHz, CDCl₃)
O
S
O
(8)
6.00
f1 (ppm)

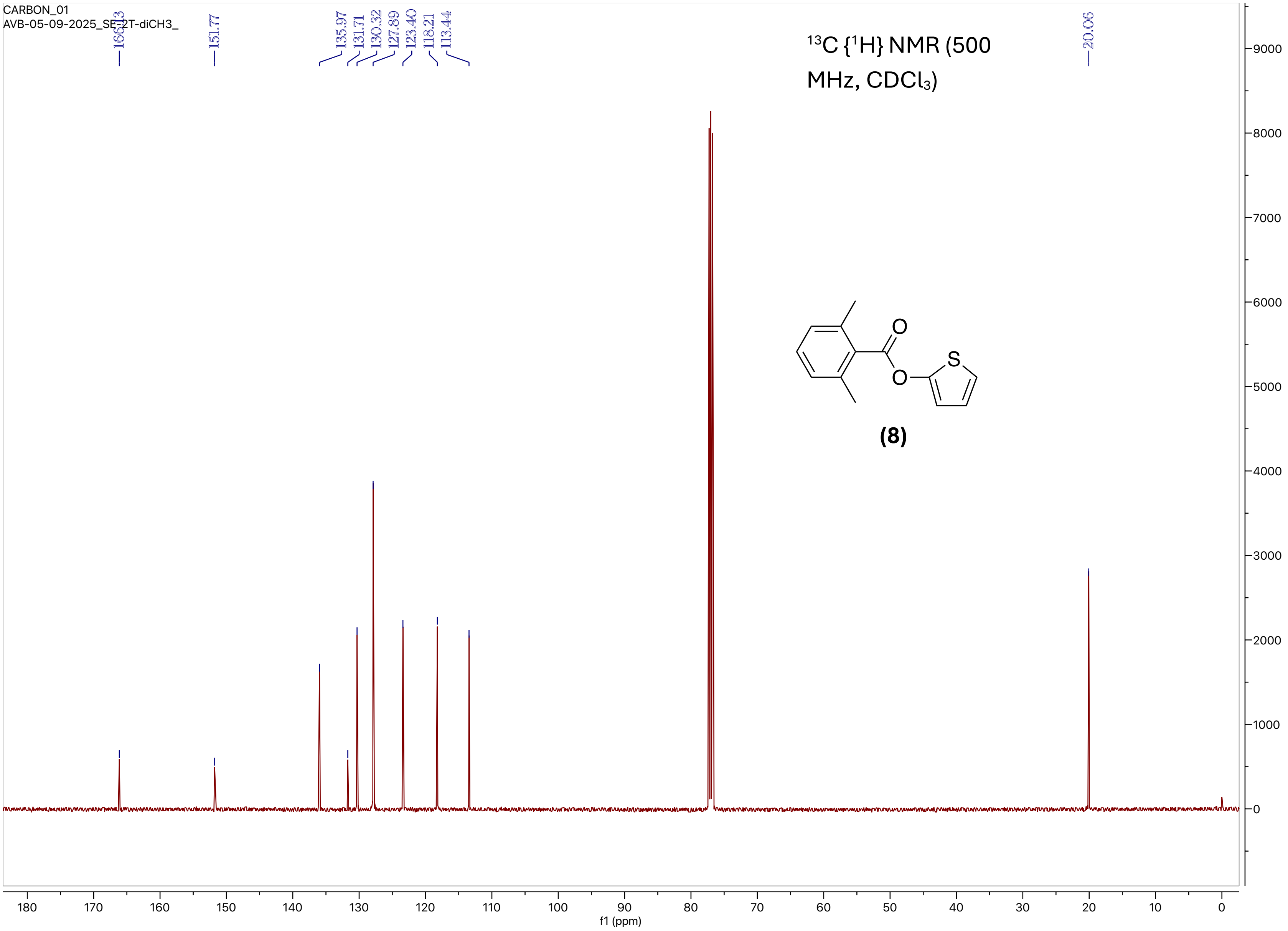
CARBON_01
AVB-05-09-2025_SE-2T-diCH3_
166.13
151.77
135.97
131.71
130.32
127.89
123.40
118.21
113.44
20.06
13C {1H} NMR (500 MHz, CDCl3)
O
O
S
(8)
9000
8000
7000
6000
5000
4000
3000
2000
1000
0
180
170
160
150
140
130
120
110
100
90
80
70
60
50
40
30
20
10
0
f1 (ppm)

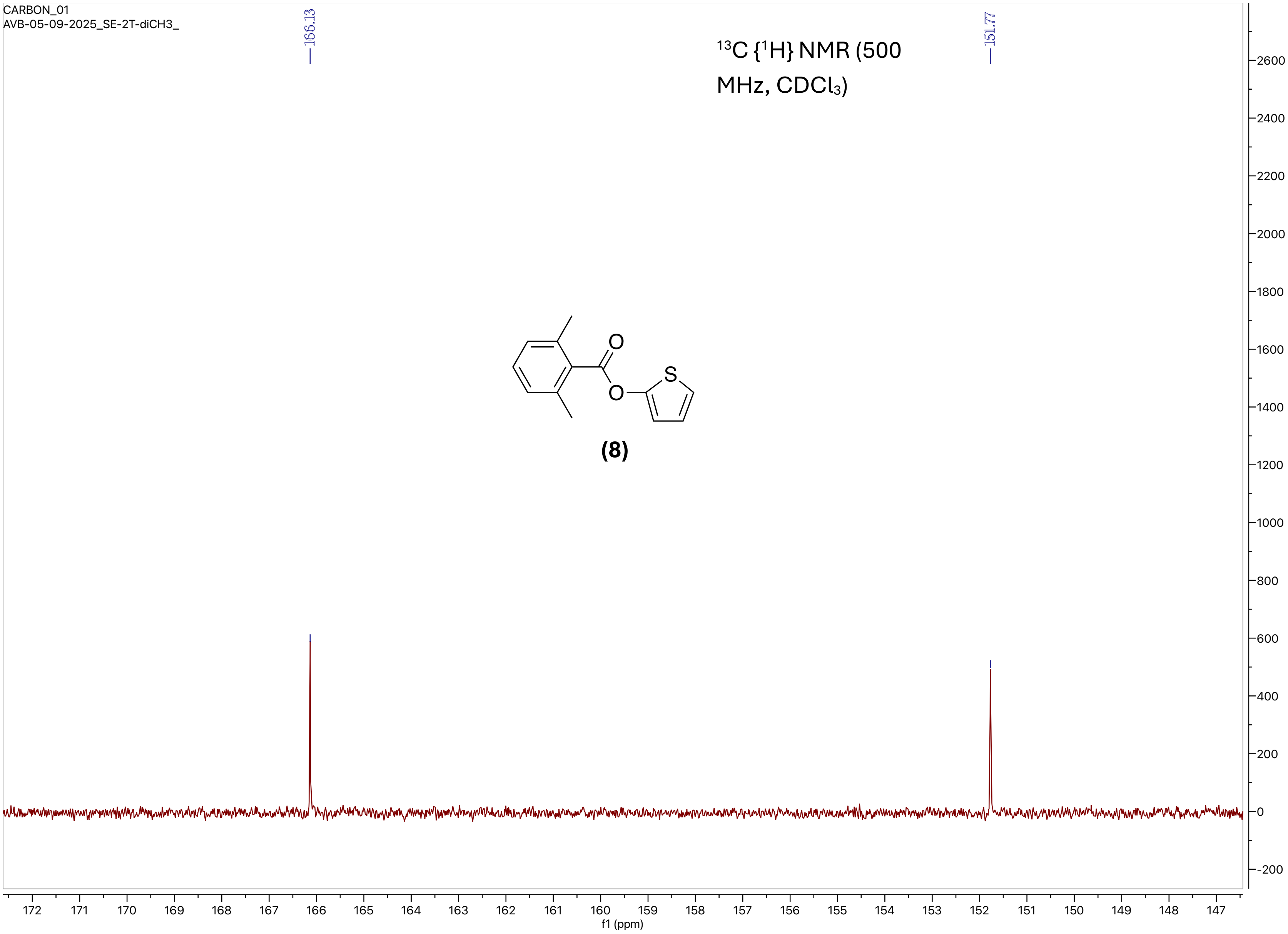
CARBON_01
AVB-05-09-2025_SE-2T-diCH3_
166.13
151.77
$^{13}C\{^{1}H\}$ NMR (500 MHz, $CDCl_3$)
O
O
S
(8)
2600
2400
2200
2000
1800
1600
1400
1200
1000
800
600
400
200
0
-200
172 171 170 169 168 167 166 165 164 163 162 161 160 159 158 157 156 155 154 153 152 151 150 149 148 147
f1 (ppm)

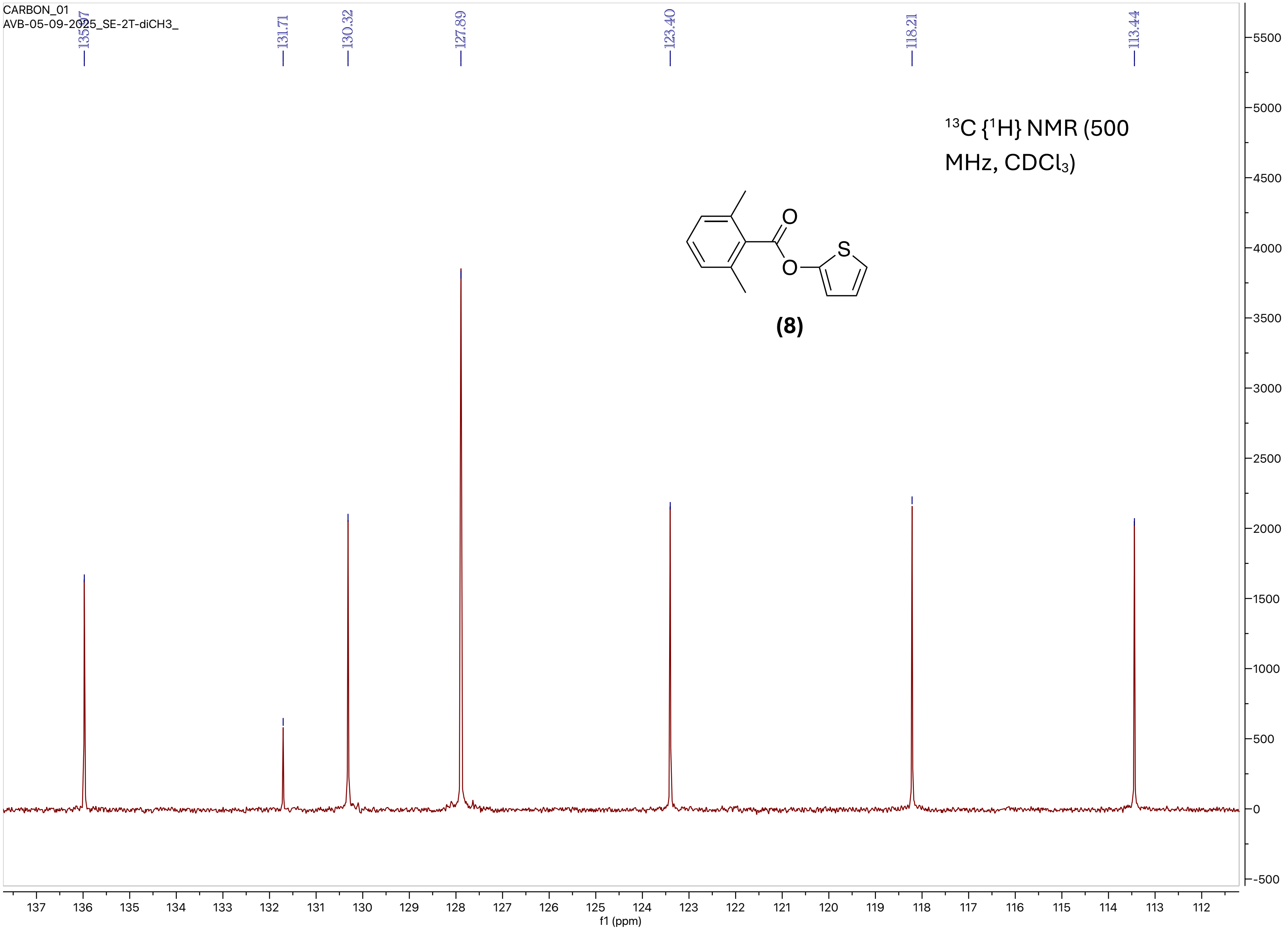
CARBON_01
AVB-05-09-2025_SE-2T-diCH3_
135.97
131.71
130.32
127.89
123.40
118.21
113.44
$^{13}C\{^{1}H\}$ NMR (500 MHz, $CDCl_3$)
O
O
S
(8)
137 136 135 134 133 132 131 130 129 128 127 126 125 124 123 122 121 120 119 118 117 116 115 114 113 112
f1 (ppm)
5500 5000 4500 4000 3500 3000 2500 2000 1500 1000 500 0 -500

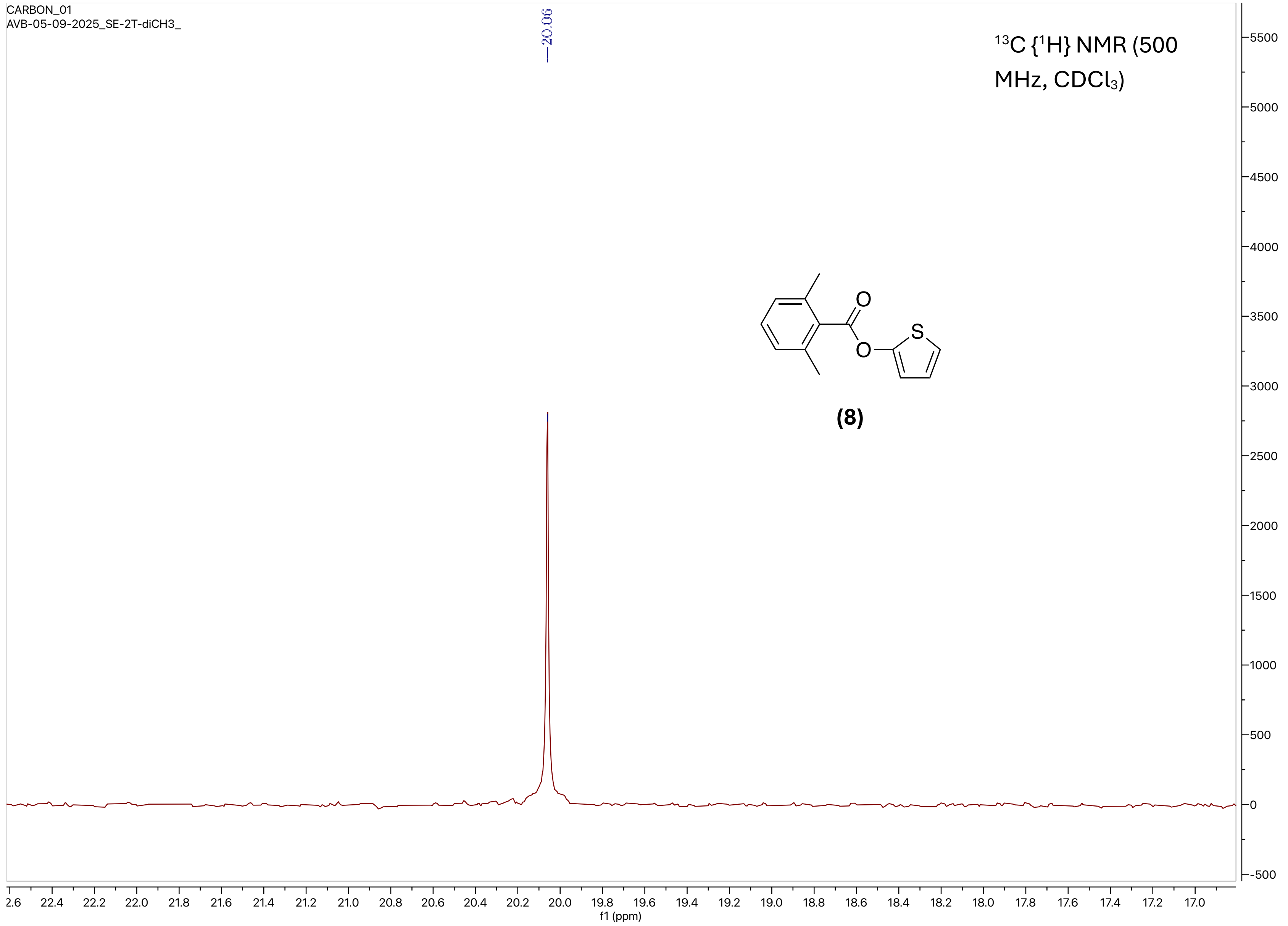
CARBON_01
AVB-05-09-2025_SE-2T-diCH3_
20.06
$^{13}C\{^1H\}$ NMR (500 MHz, $CDCl_3$)
O
O
S
(8)
f1 (ppm)

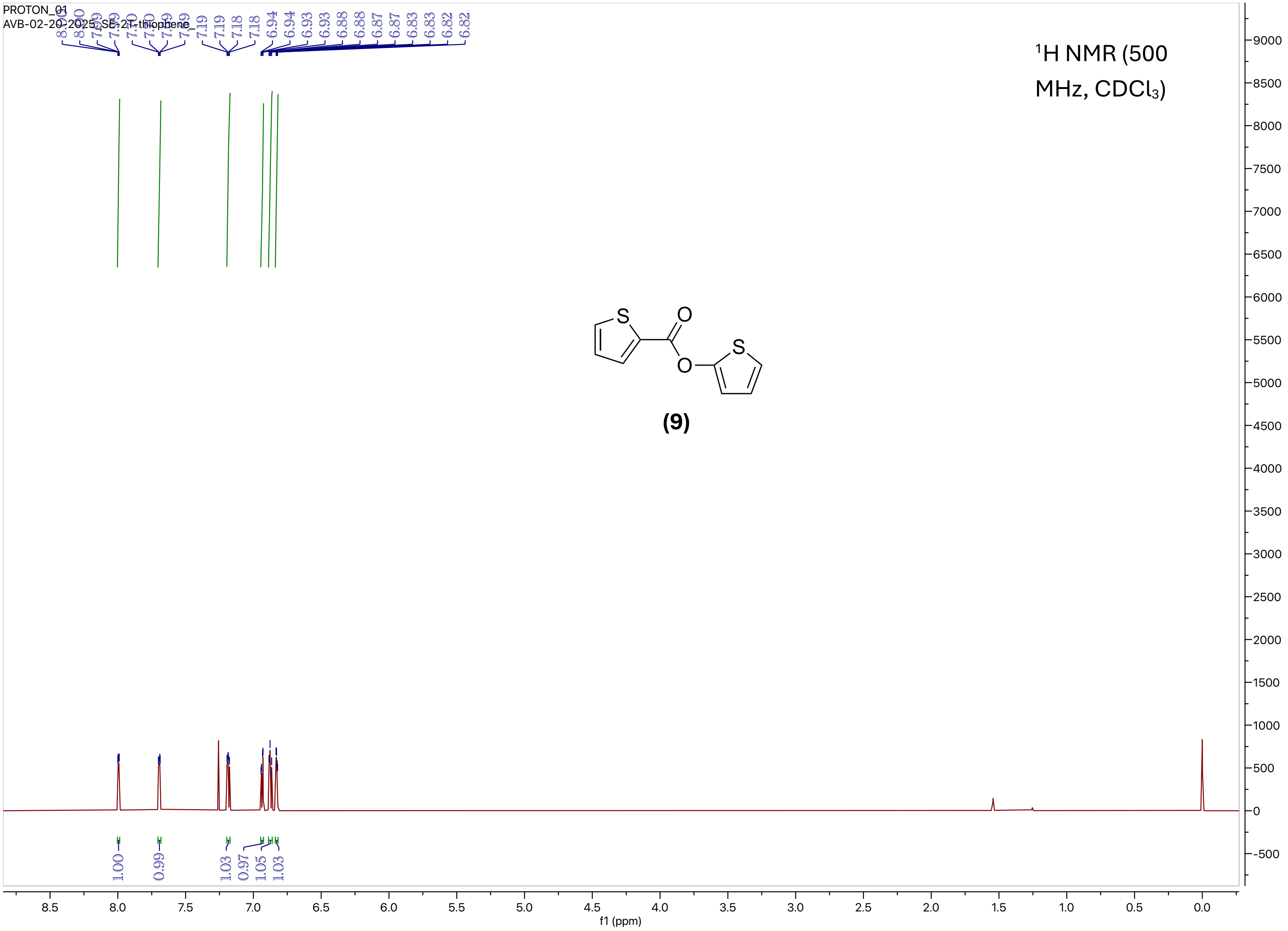
PROTON_01
AVB-02-20-2025_SE-2T-thiophene_
8.00
8.00
7.99
7.99
7.70
7.70
7.69
7.69
7.19
7.19
7.18
7.18
6.94
6.94
6.93
6.93
6.88
6.88
6.87
6.87
6.83
6.83
6.82
6.82
1H NMR (500 MHz, CDCl3)
S
O
O
S
(9)
1.00
0.99
1.03
0.97
1.05
1.03
f1 (ppm)

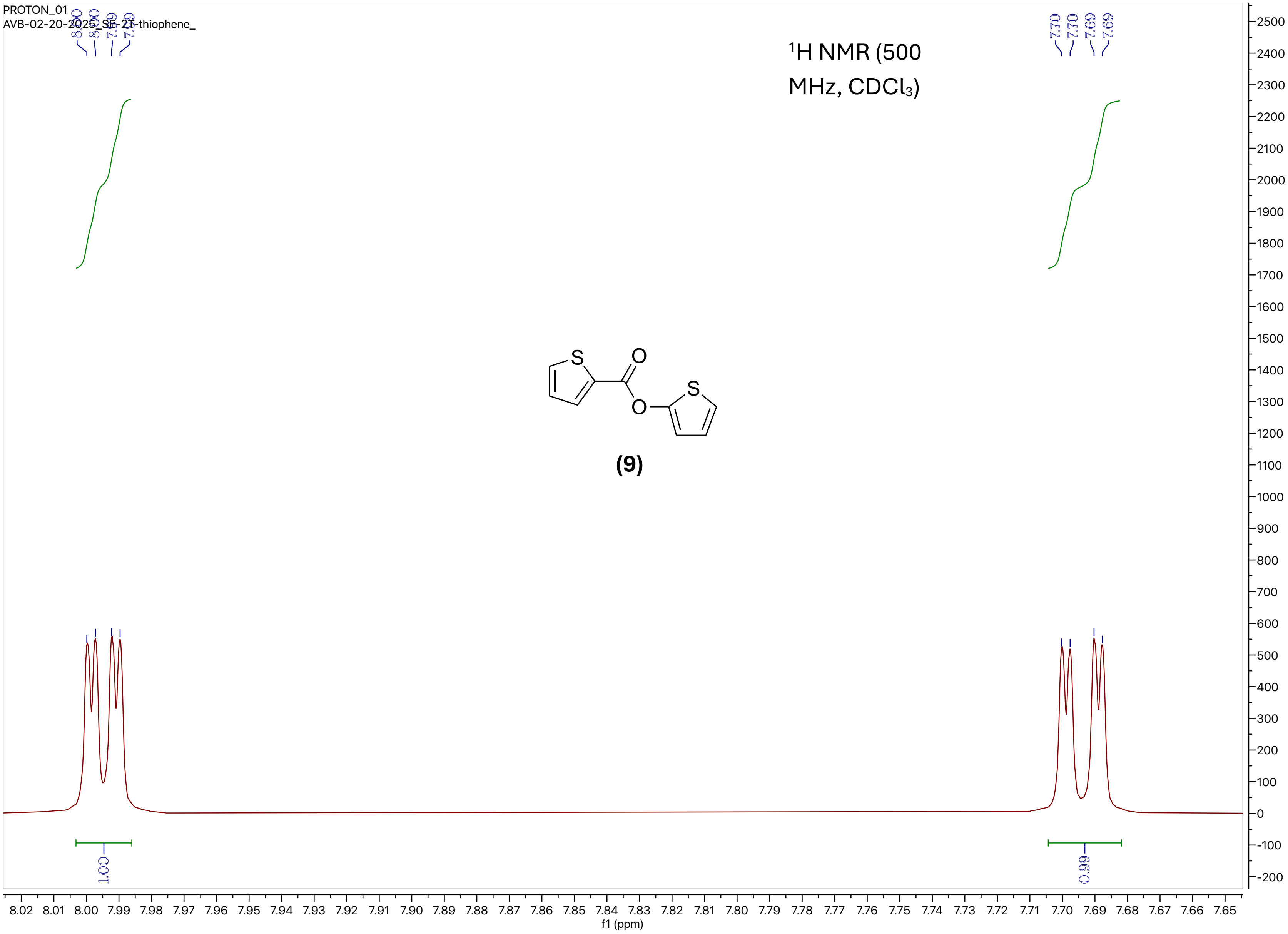

PROTON_01
AVB-02-20-2025_SE-21-thiophene_
^{1}H NMR (500 MHz, $CDCl_3$)
(9)
f1 (ppm)

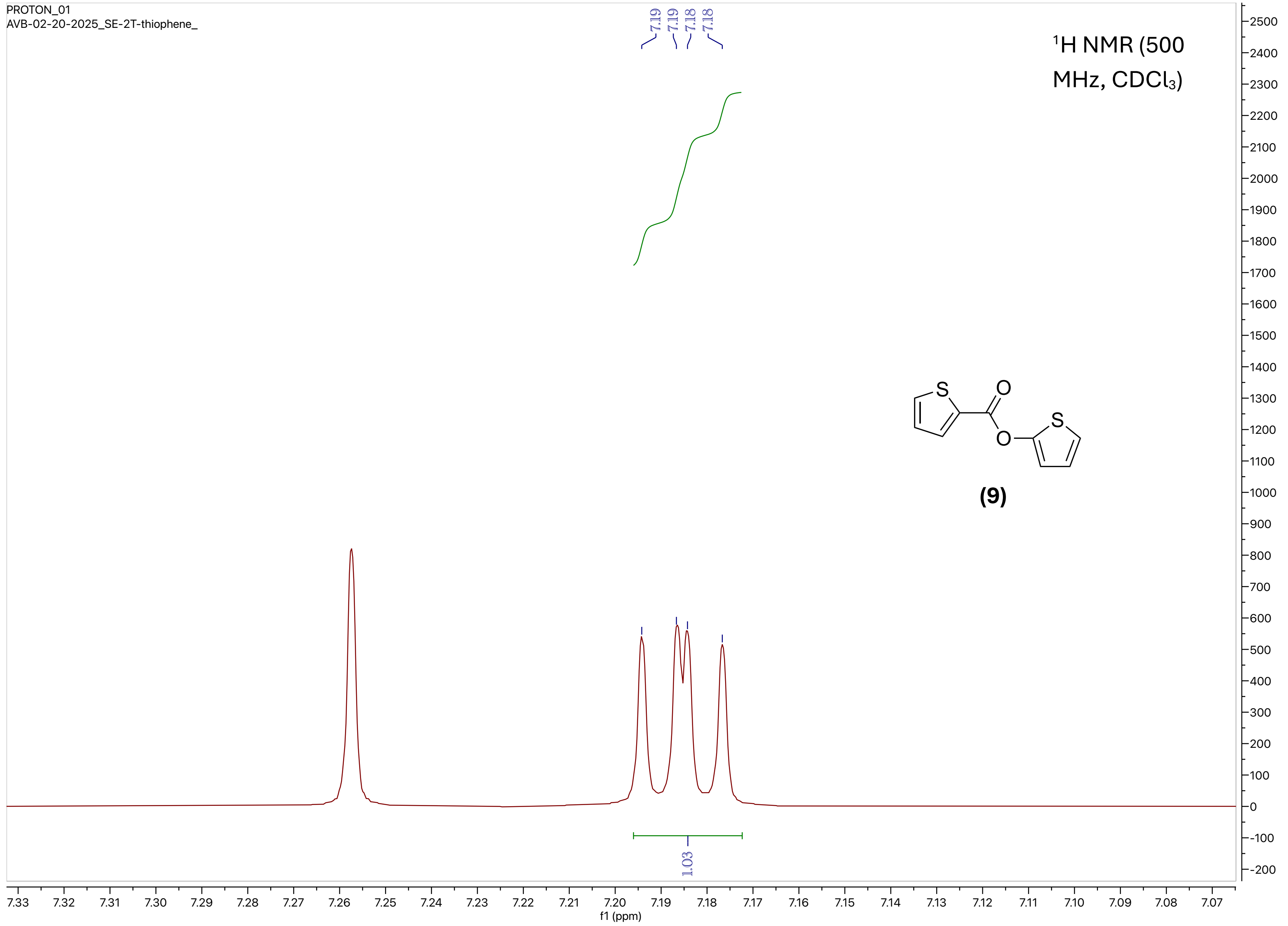
PROTON_01
AVB-02-20-2025_SE-2T-thiophene_
7.19
7.19
7.18
7.18
1H NMR (500 MHz, CDCl3)
S
O
O
S
(9)
1.03
f1 (ppm)

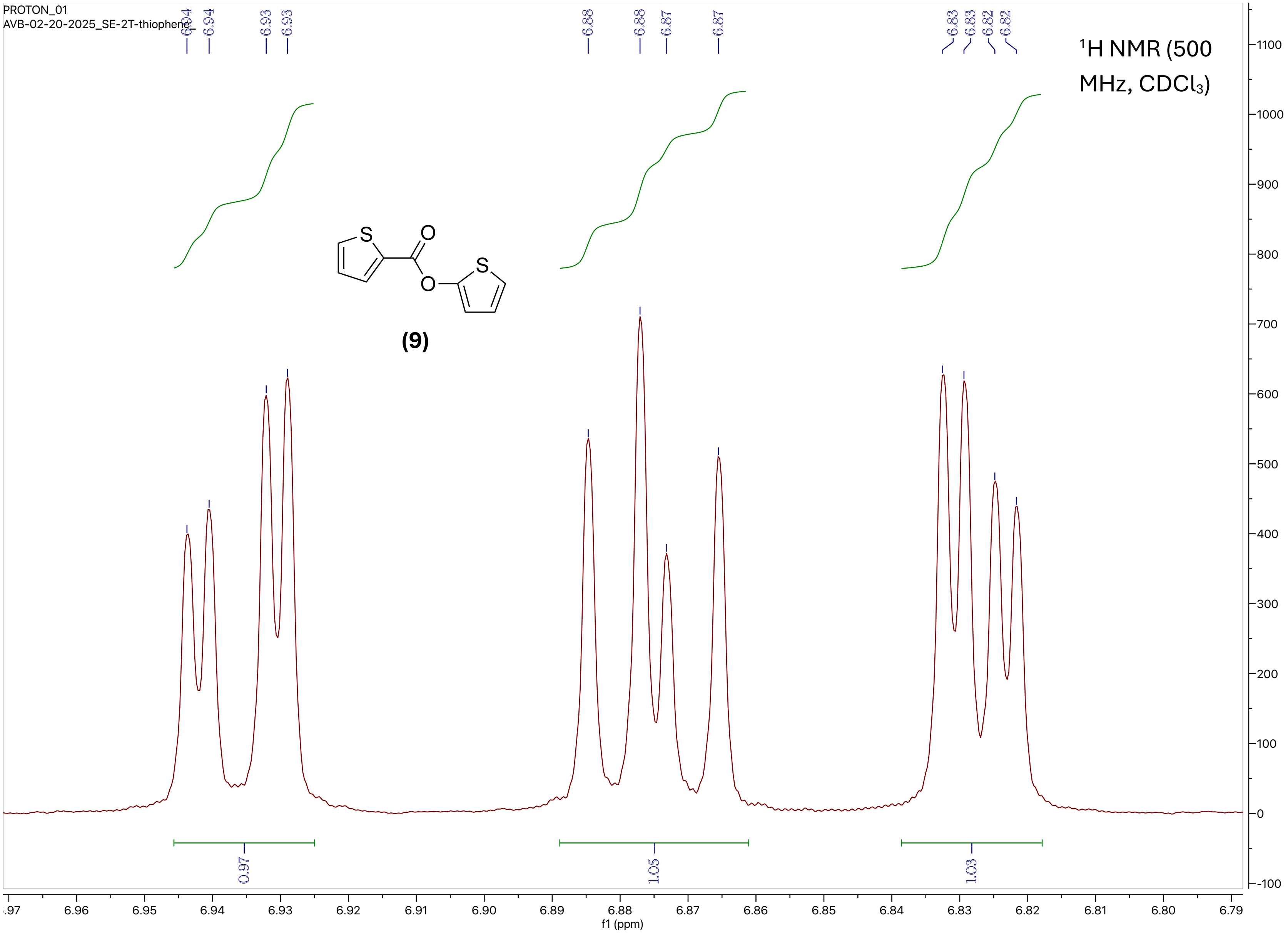
PROTON_01
AVB-02-20-2025_SE-2T-thiophene_
6.94
6.94
6.93
6.93
6.88
6.88
6.87
6.87
6.83
6.83
6.82
6.82
^{1}H NMR (500 MHz, $CDCl_3$)
(9)
0.97
1.05
1.03
f1 (ppm)

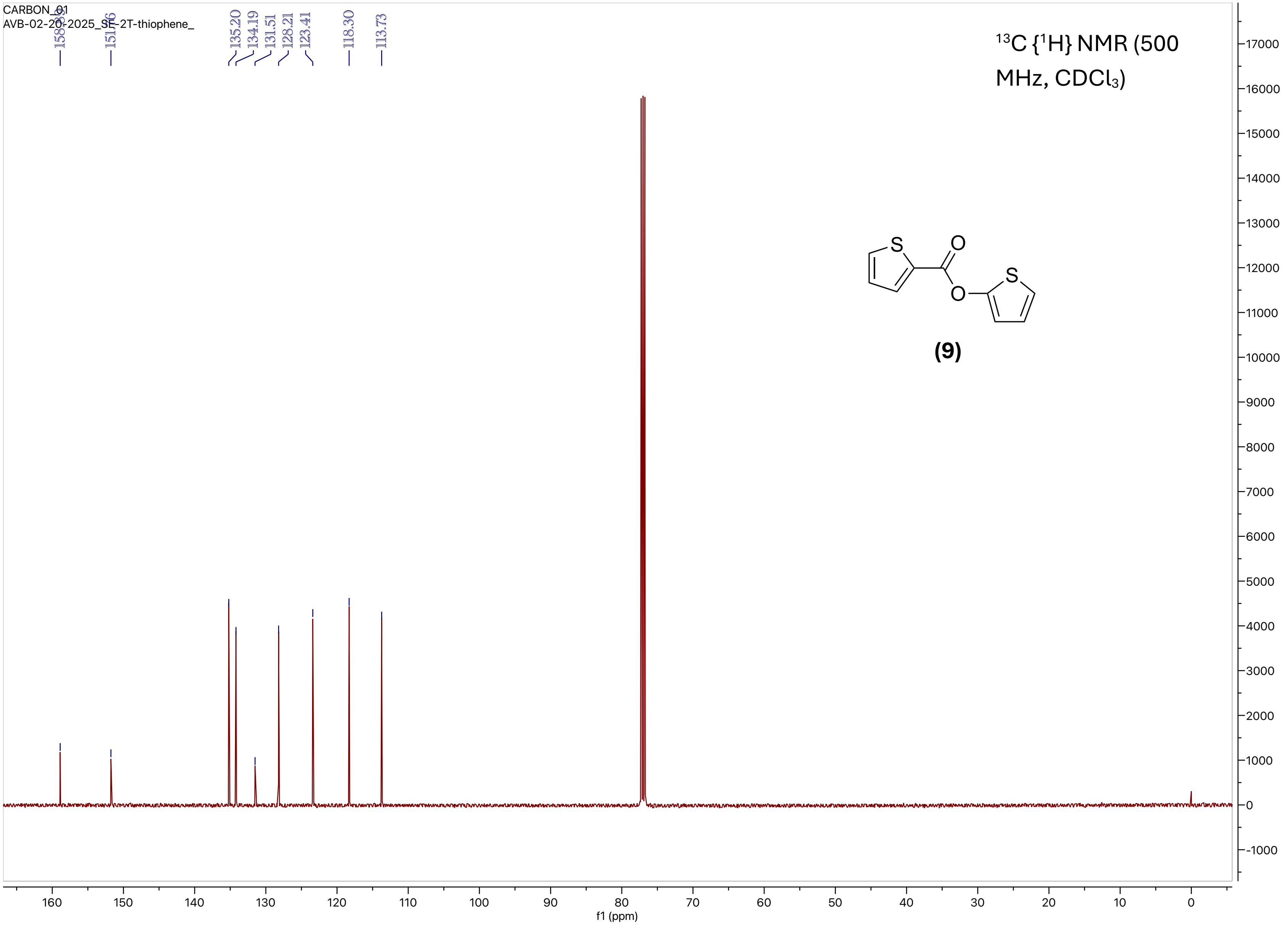
CARBON_01
AVB-02-20-2025_SE-2T-thiophene_
135.20
134.19
131.51
128.21
123.41
118.30
113.73
13C {1H} NMR (500 MHz, CDCl3)
(9)
f1 (ppm)

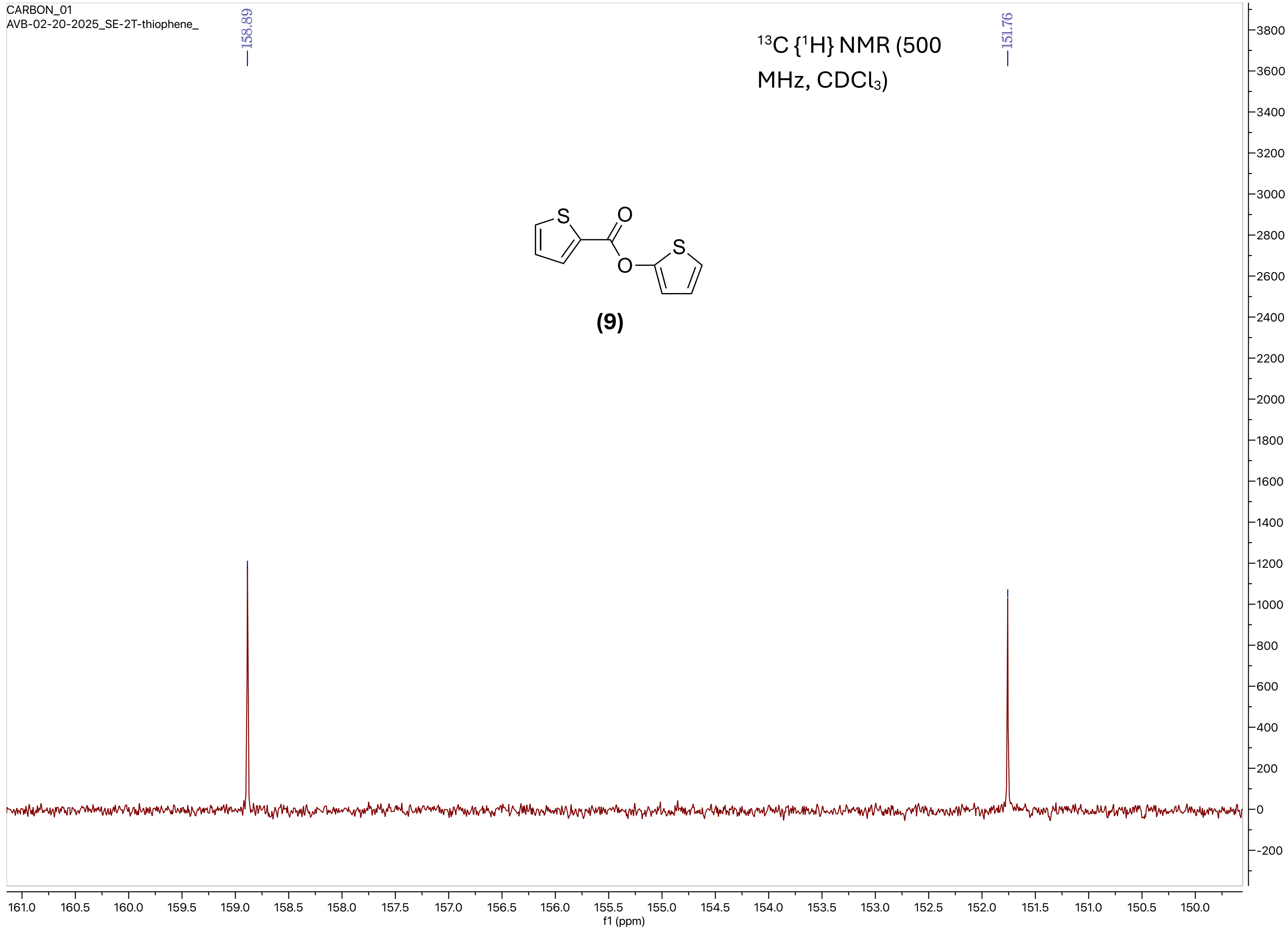

CARBON_01
AVB-02-20-2025_SE-2T-thiophene_
158.89
151.76
13C {1H} NMR (500 MHz, CDCl3)
S
O
O
S
(9)
f1 (ppm)

$^{13}C\{^1H\}$ NMR (500 MHz, $CDCl_3$)

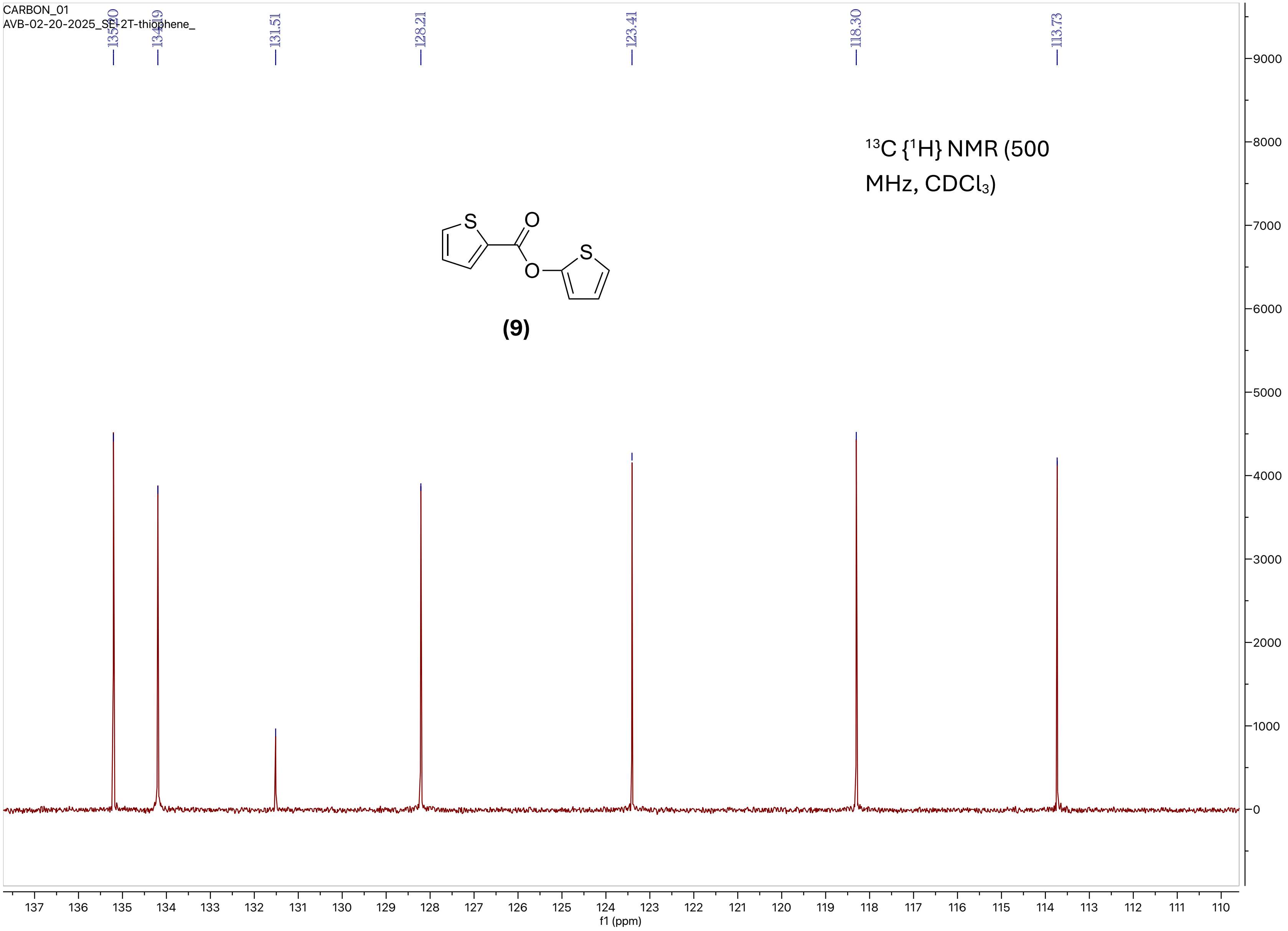

(9)

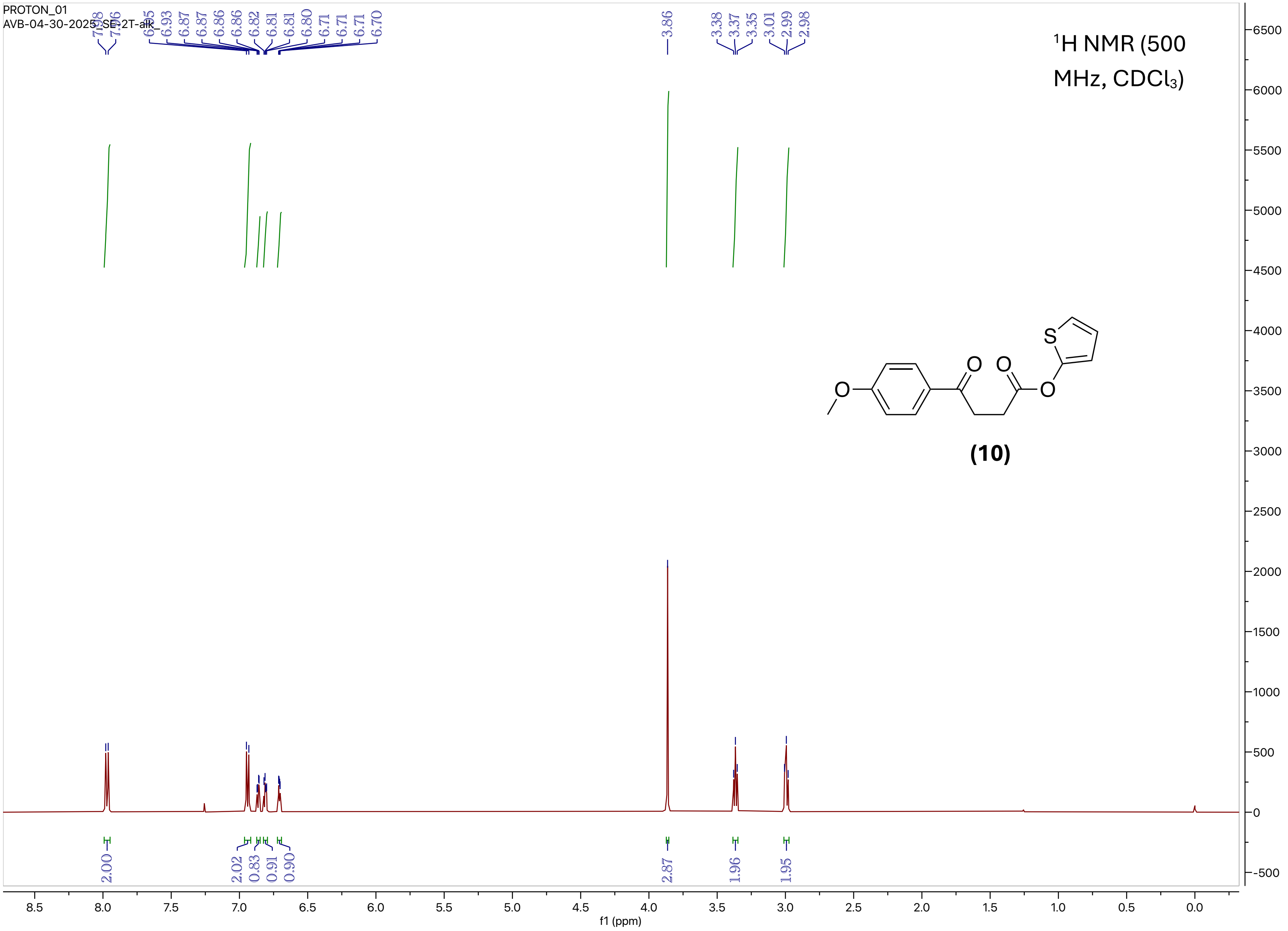

^{1}H NMR (500 MHz, $CDCl_3$)

(10)

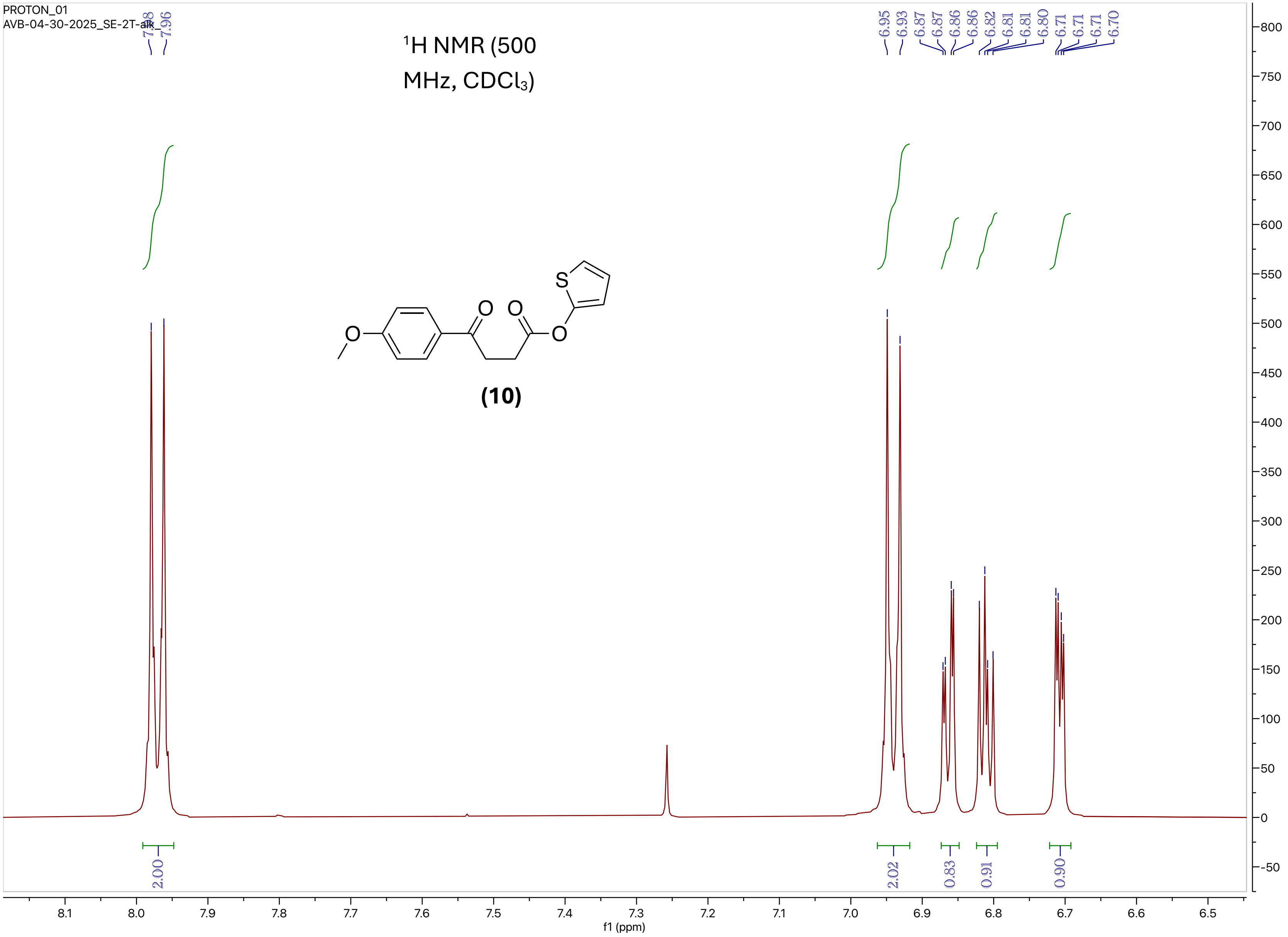
PROTON_01
AVB-04-30-2025_SE-2T-alk_
7.98
7.96
6.95
6.93
6.87
6.87
6.86
6.86
6.82
6.81
6.81
6.80
6.71
6.71
6.71
6.70
1H NMR (500 MHz, CDCl3)
S
O
O
O
O
(10)
2.00
2.02
0.83
0.91
0.90
f1 (ppm)

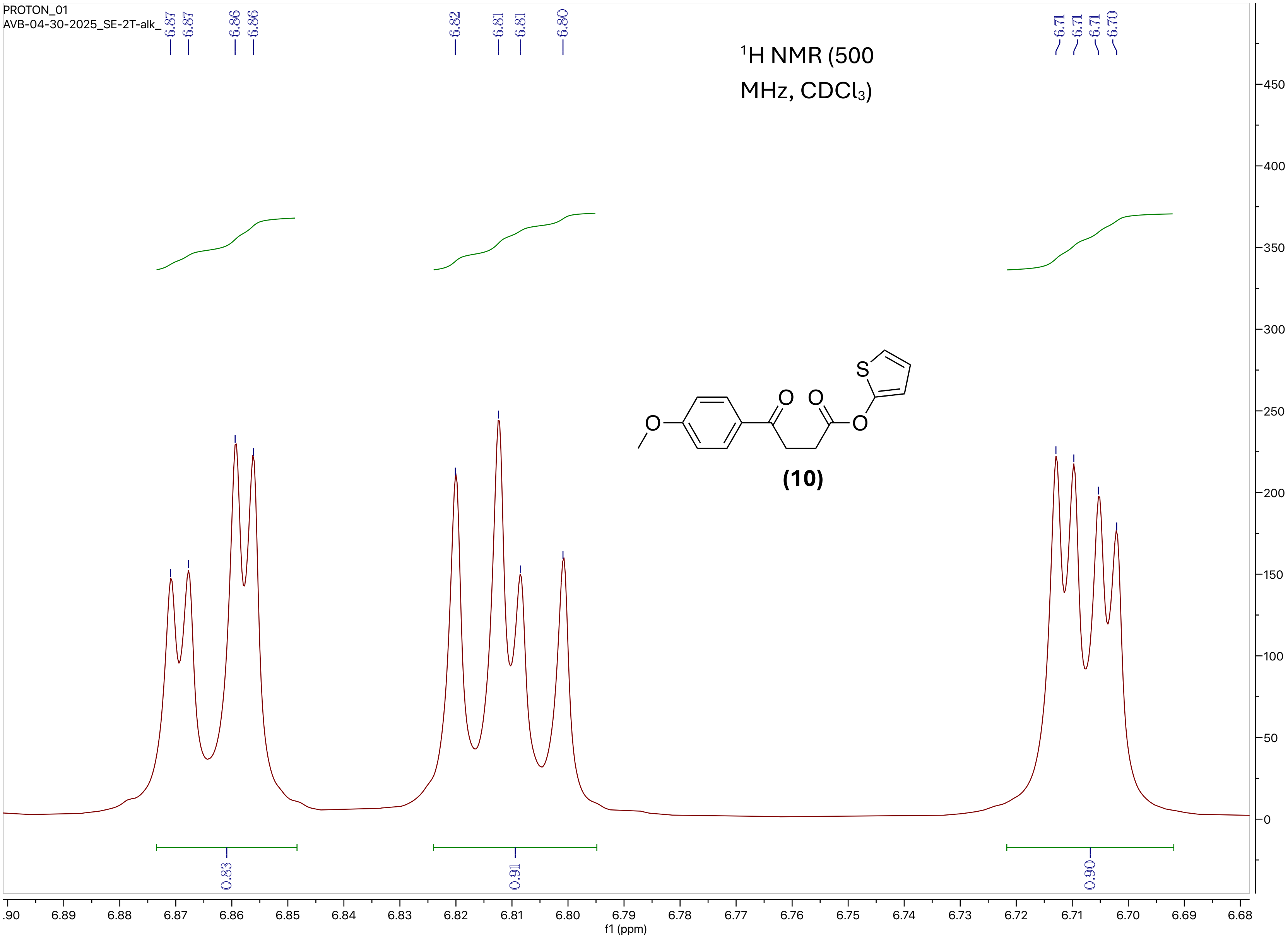
PROTON_01
AVB-04-30-2025_SE-2T-alk_
6.87
6.87
6.86
6.86
6.82
6.81
6.81
6.80
6.71
6.71
6.71
6.70
1H NMR (500 MHz, CDCl3)
O
O
O
O
S
(10)
0.83
0.91
0.90
f1 (ppm)

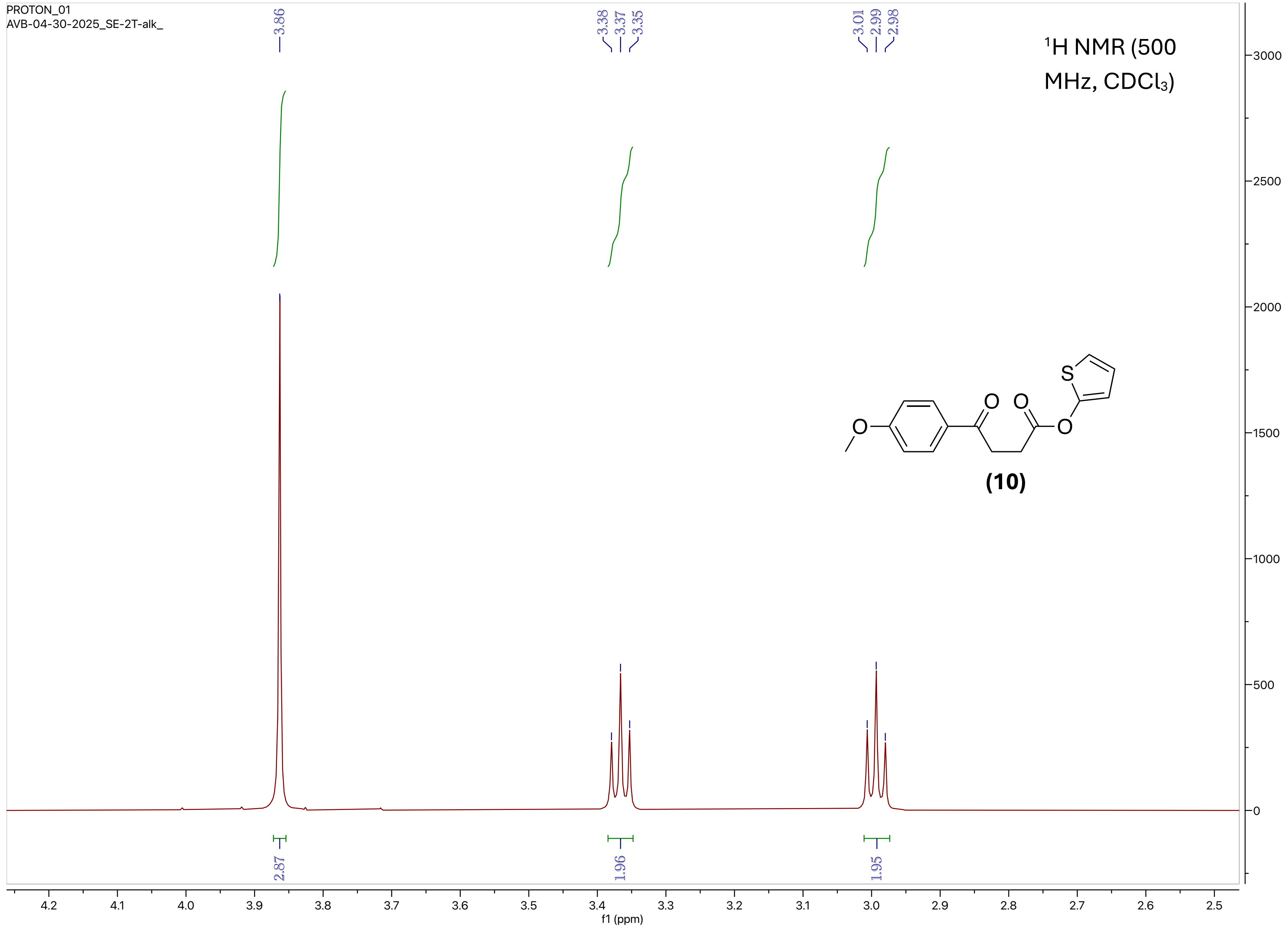
PROTON_01
AVB-04-30-2025_SE-2T-alk_
3.86
3.38
3.37
3.35
3.01
2.99
2.98
1H NMR (500 MHz, CDCl3)
O
O
O
O
S
(10)
2.87
1.96
1.95
f1 (ppm)

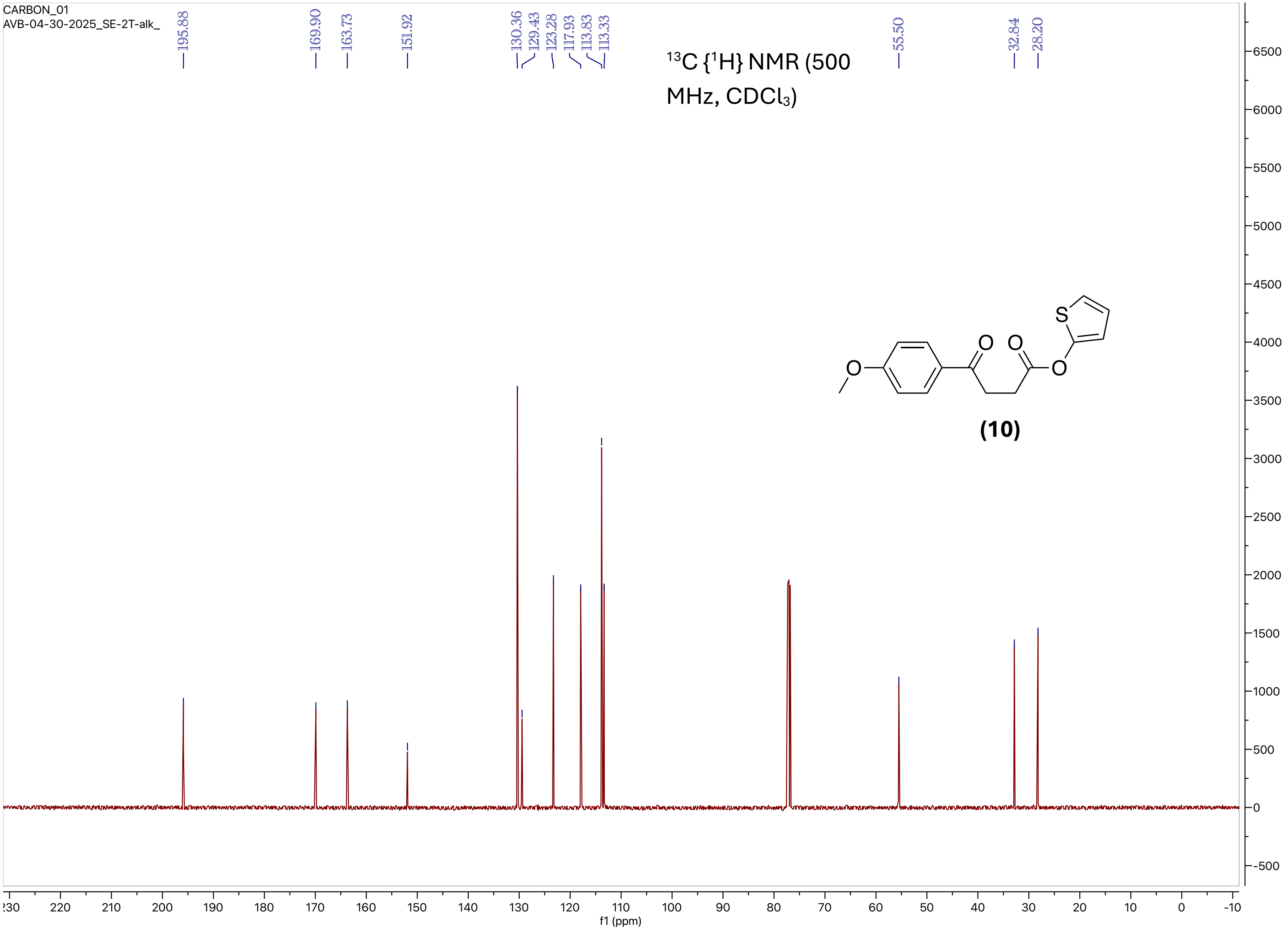
CARBON_01
AVB-04-30-2025_SE-2T-alk_
195.88
169.90
163.73
151.92
130.36
129.43
123.28
117.93
113.83
113.33
55.50
32.84
28.20
13C {1H} NMR (500 MHz, CDCl3)
(10)
f1 (ppm)

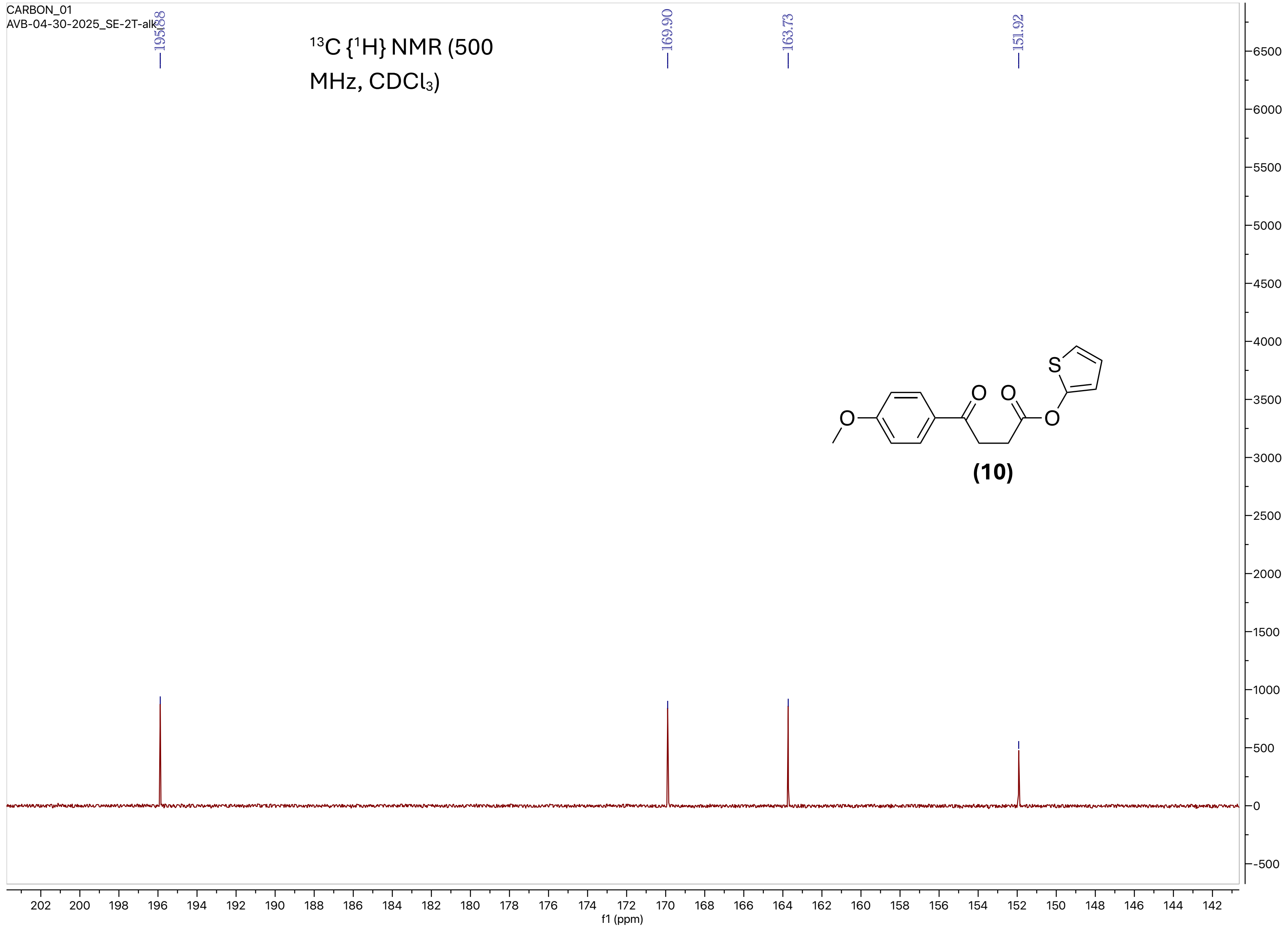

CARBON_01
AVB-04-30-2025_SE-2T-alk
195.88
169.90
163.73
151.92
^{13}C {^{1}H} NMR (500 MHz, $CDCl_3$)
(10)
f1 (ppm)

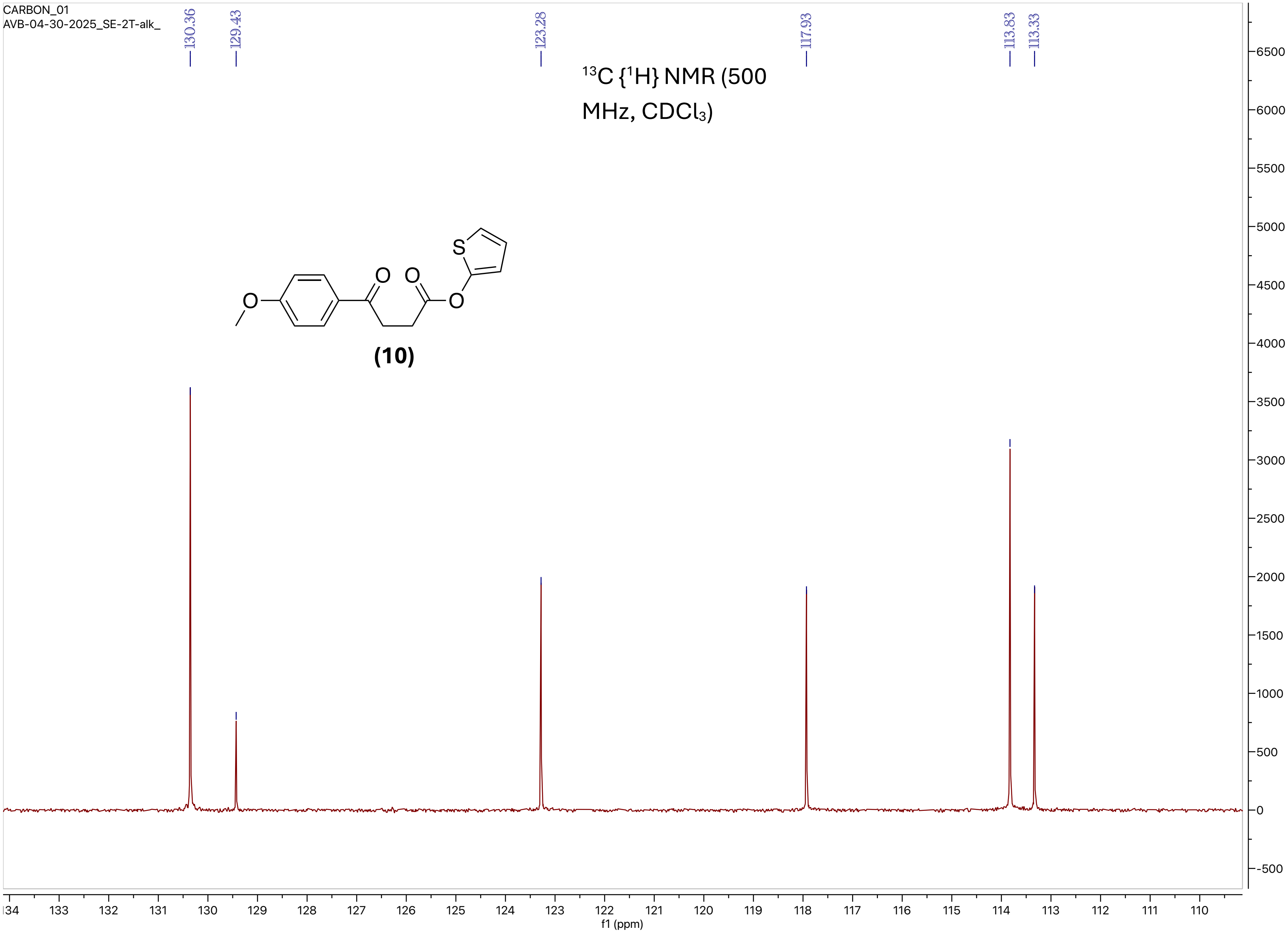

CARBON_01
AVB-04-30-2025_SE-2T-alk_
130.36
129.43
123.28
117.93
113.83
113.33
¹³C {¹H} NMR (500 MHz, CDCl₃)
(10)
f1 (ppm)

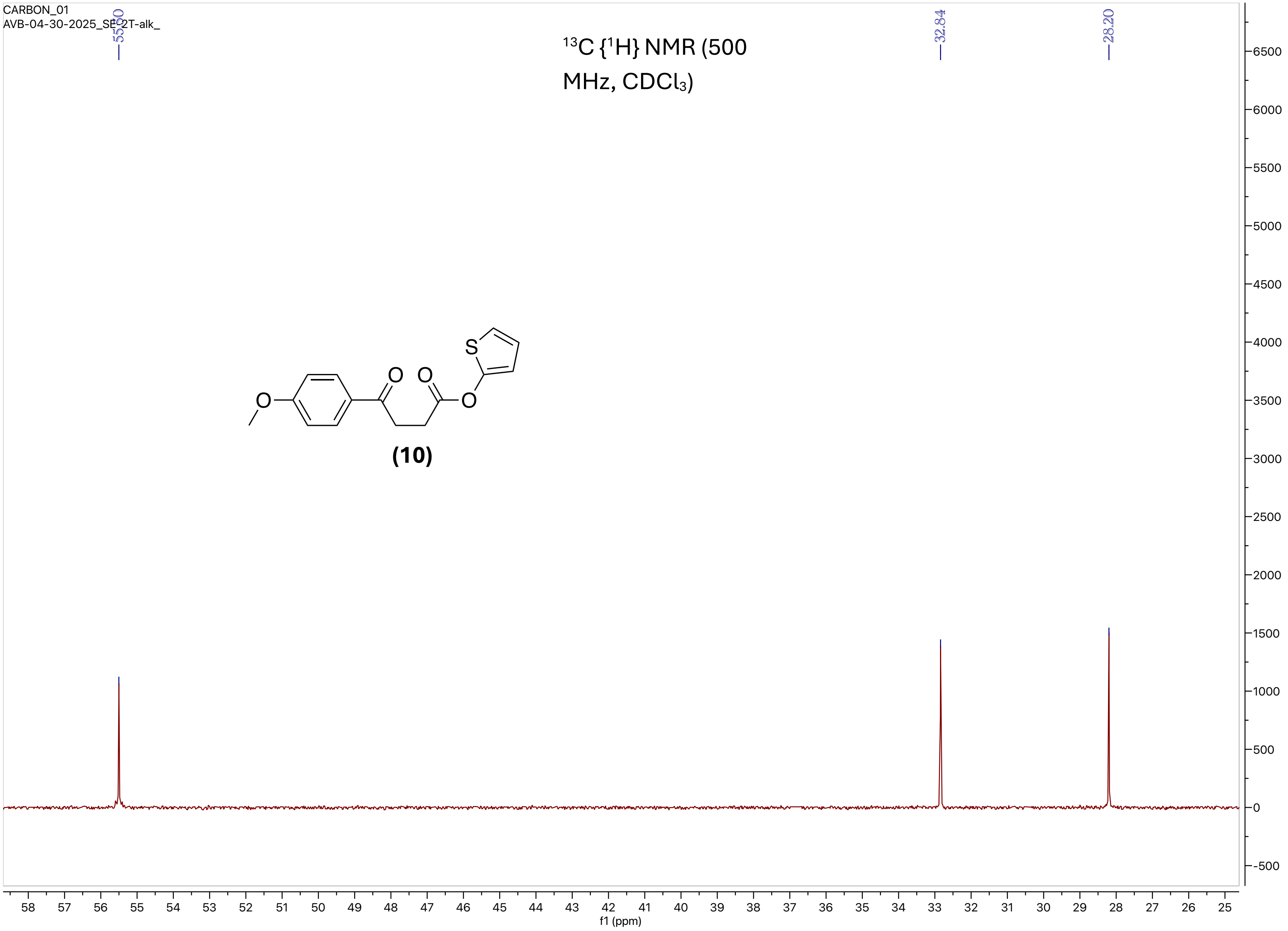
CARBON_01
AVB-04-30-2025_SE-2T-alk_
13C {1H} NMR (500 MHz, CDCl3)
32.84
28.20
(10)
f1 (ppm)

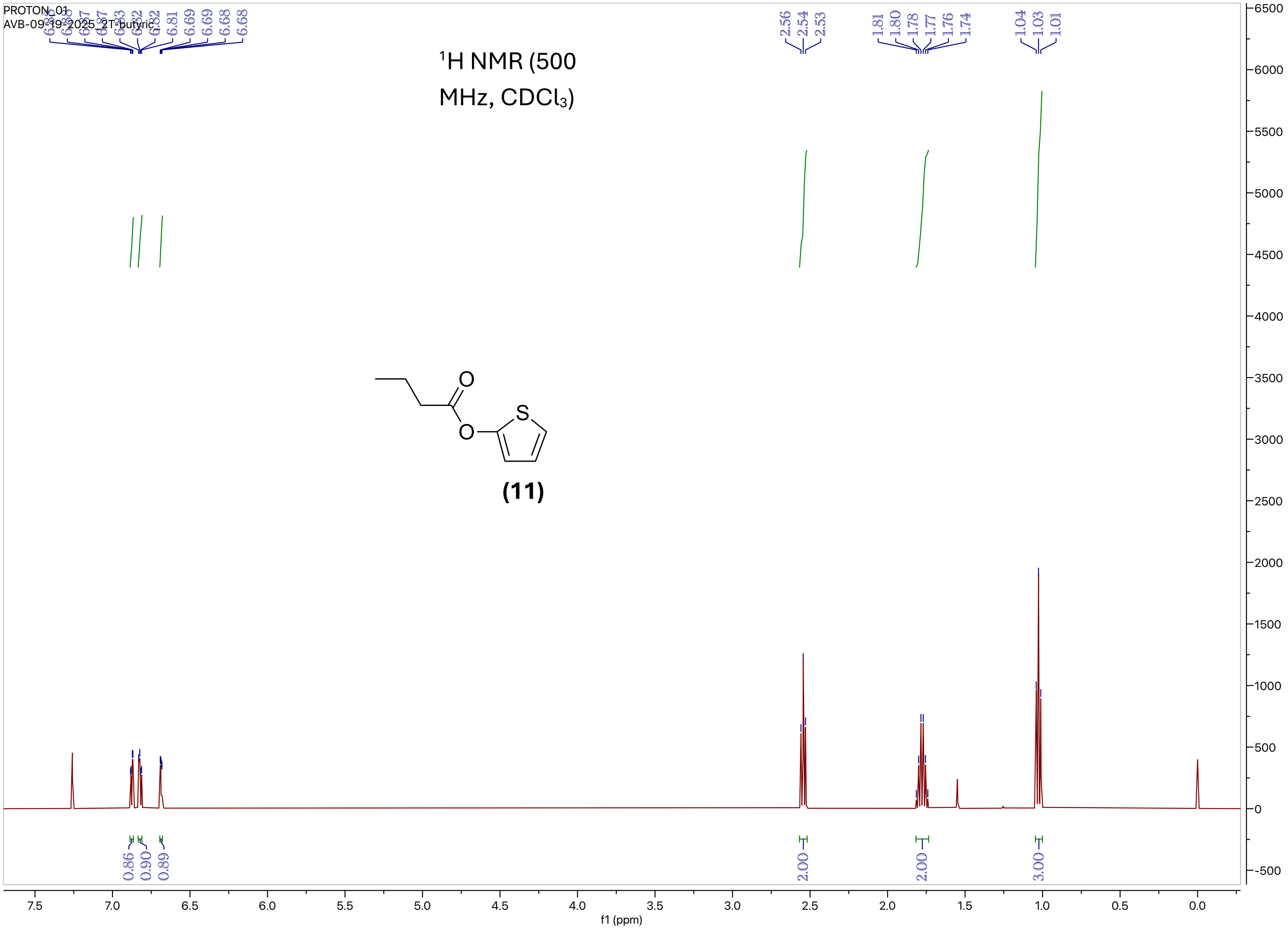
PROTON_01
AVB-09-19-2025_2T-butyric
^{1}H NMR (500 MHz, CDCl$_3$)
(11)
2.56
2.54
2.53
1.81
1.80
1.78
1.77
1.76
1.74
1.04
1.03
1.01
6.82
6.81
6.69
6.69
6.68
6.68
0.86
0.90
0.89
2.00
2.00
3.00
f1 (ppm)

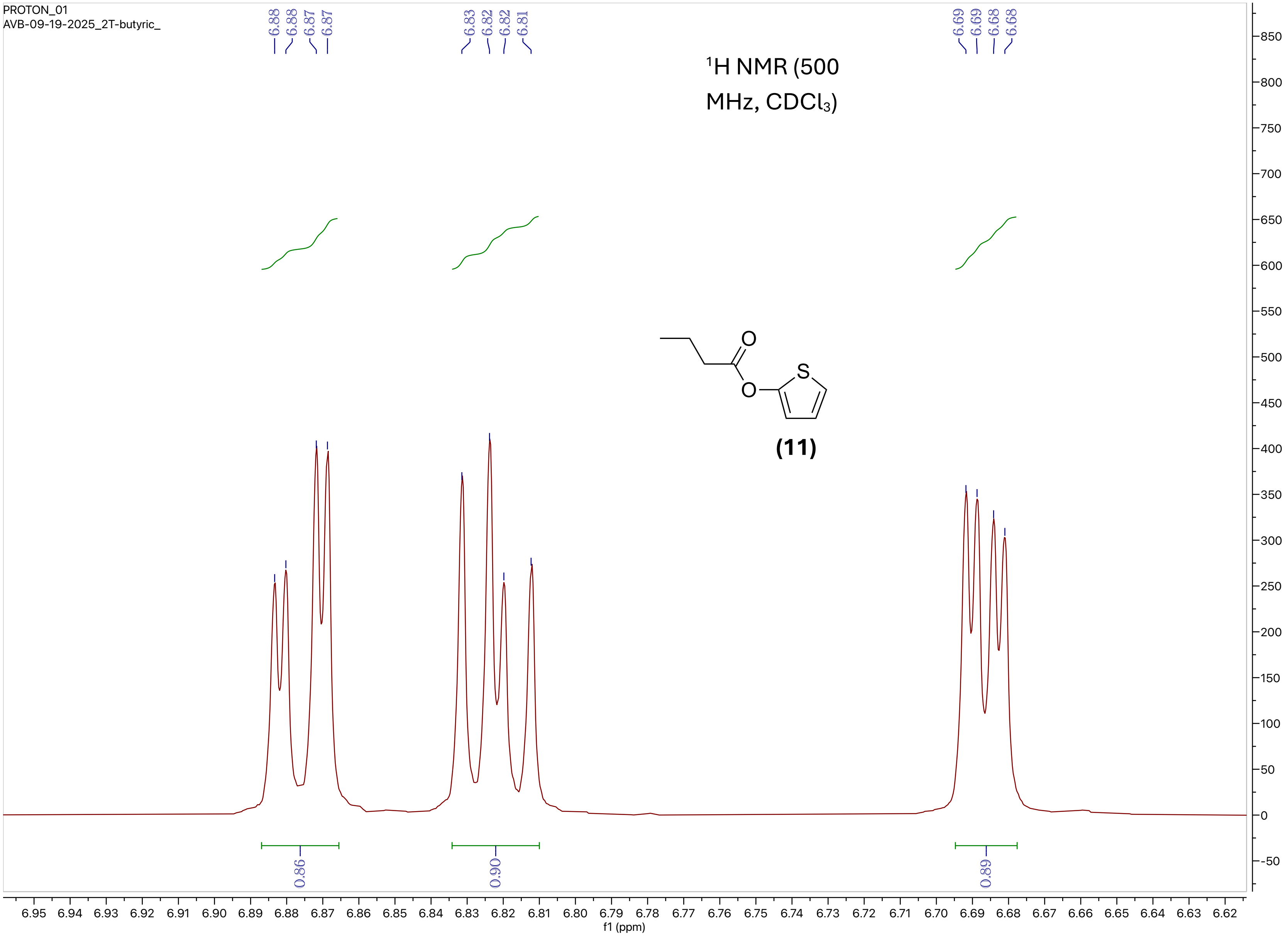

PROTON_01
AVB-09-19-2025_2T-butyric_
6.88
6.88
6.87
6.87
6.83
6.82
6.82
6.81
6.69
6.69
6.68
6.68
^{1}H NMR (500 MHz, $CDCl_3$)
O
S
O
(11)
0.86
0.90
0.89
f1 (ppm)

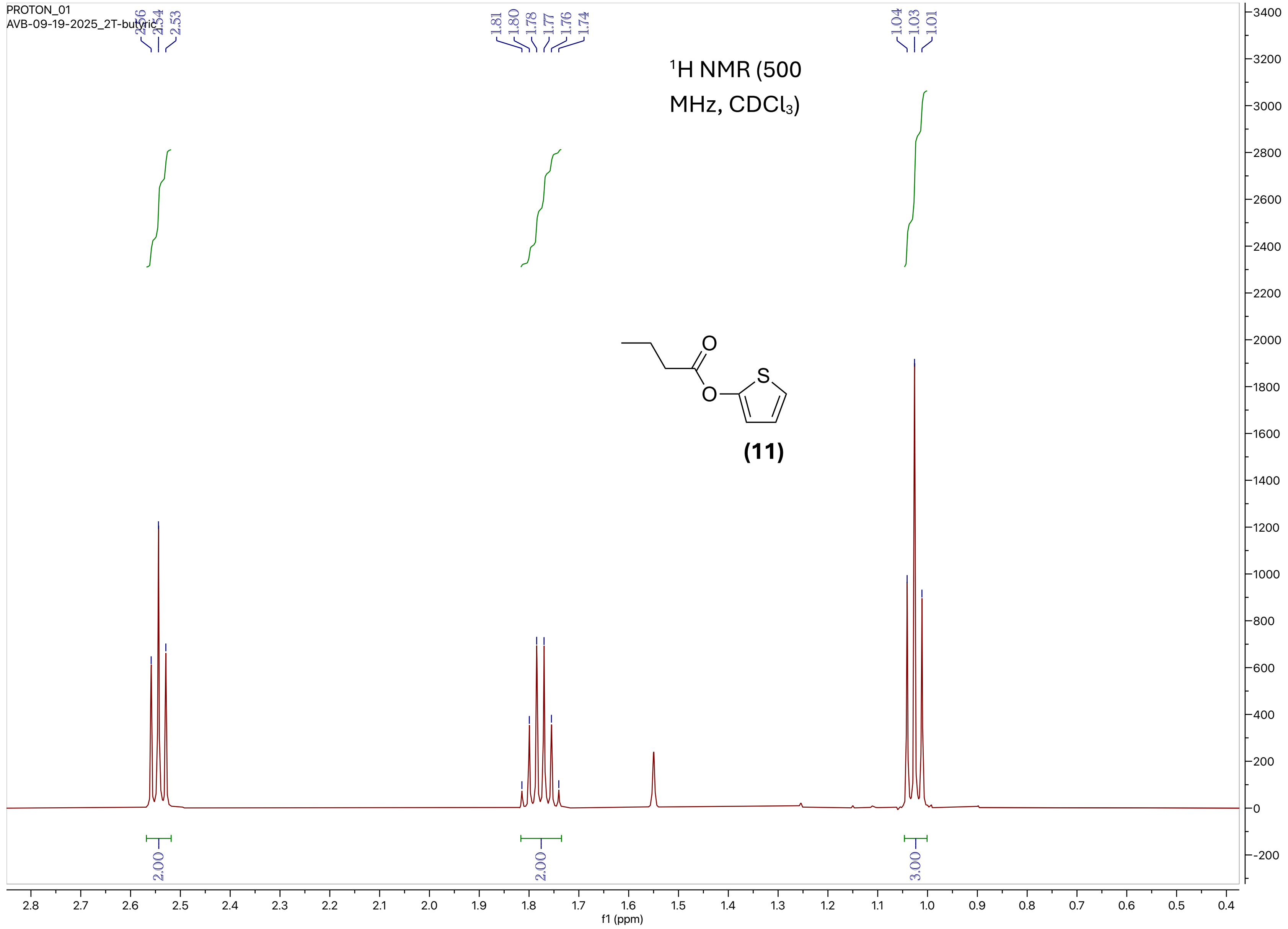
PROTON_01
AVB-09-19-2025_2T-butyric
2.56
2.54
2.53
1.81
1.80
1.78
1.77
1.76
1.74
1.04
1.03
1.01
¹H NMR (500 MHz, CDCl₃)
O
O
S
(11)
2.00
2.00
3.00
f1 (ppm)

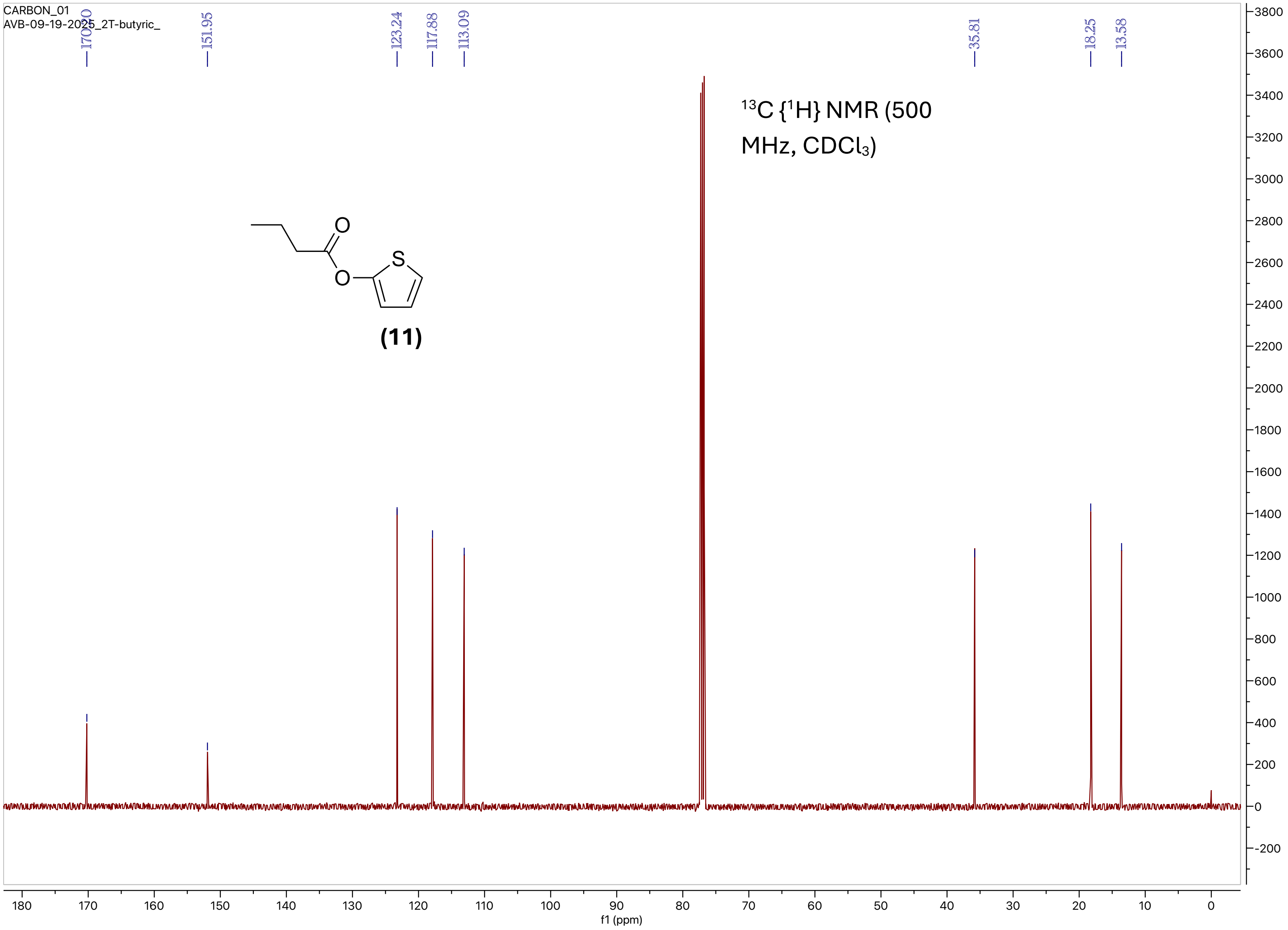
CARBON_01
AVB-09-19-2025_2T-butyric_
170.20
151.95
123.24
117.88
113.09
35.81
18.25
13.58
$^{13}C\{^{1}H\}$ NMR (500 MHz, $CDCl_3$)
O
S
O
(11)
f1 (ppm)

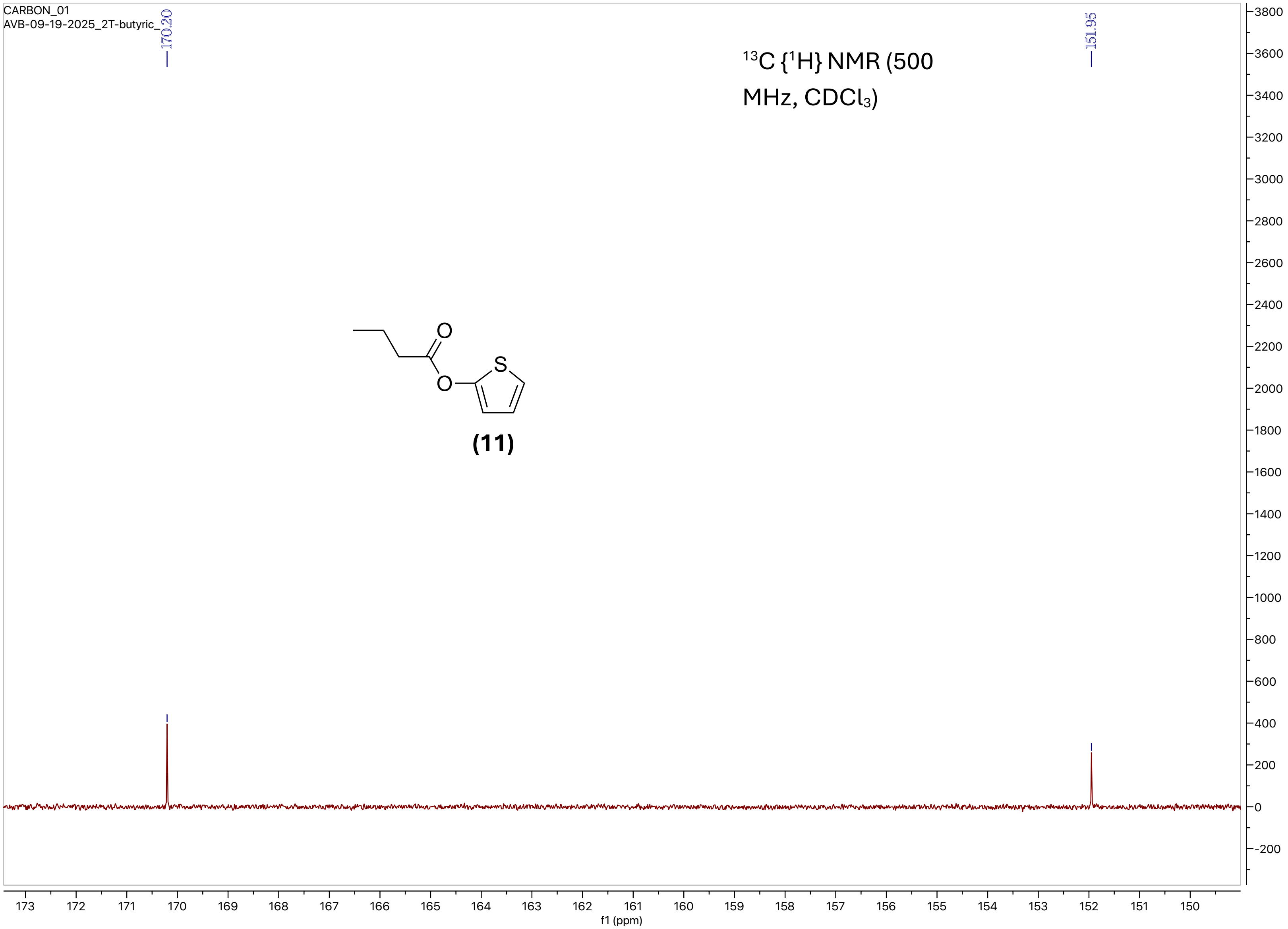
CARBON_01
AVB-09-19-2025_2T-butyric_
170.20
151.95
$^{13}C\{^1H\}$ NMR (500 MHz, $CDCl_3$)
O
O
S
(11)
f1 (ppm)

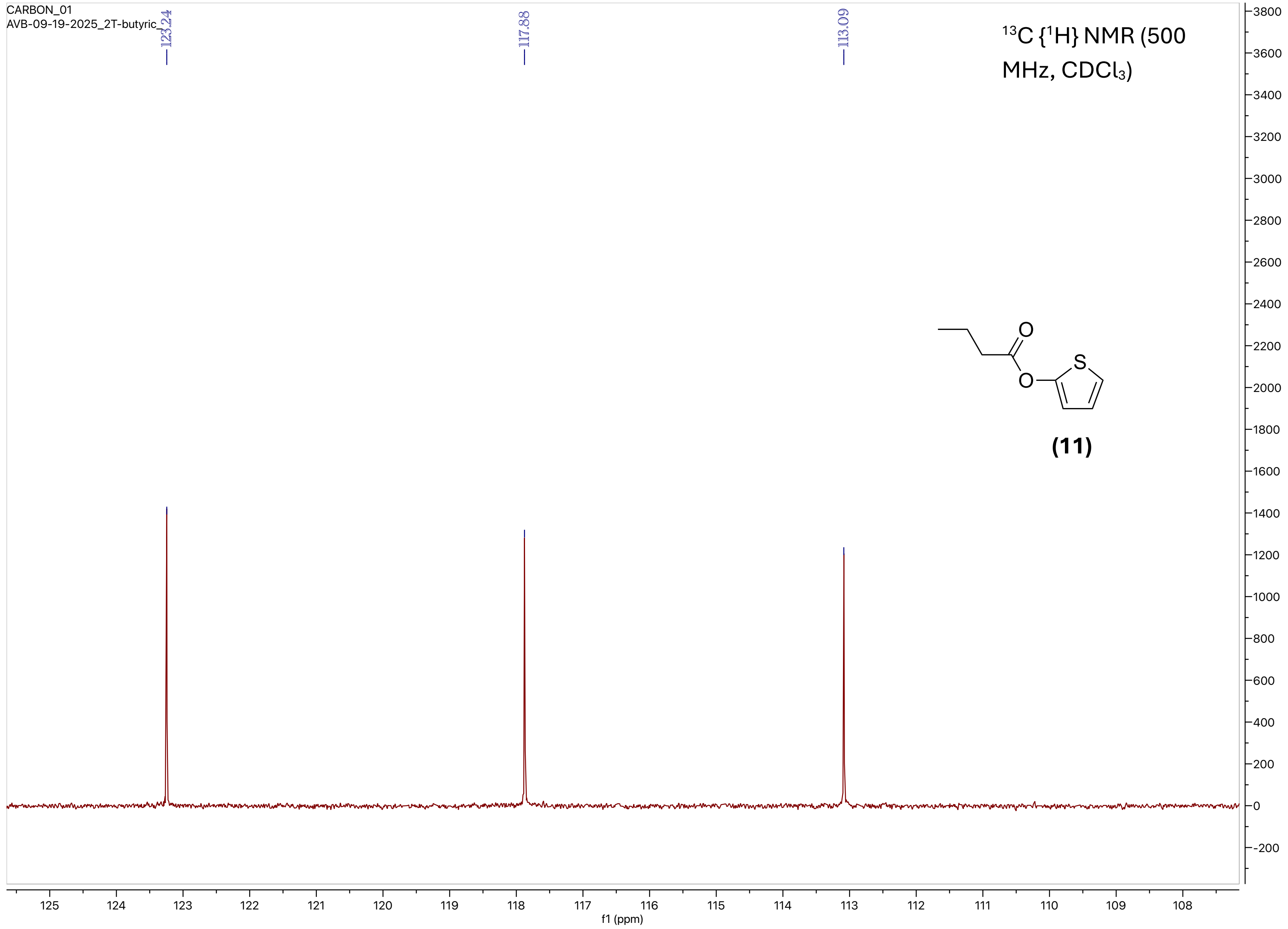
CARBON_01
AVB-09-19-2025_2T-butyric
123.24
117.88
113.09
$^{13}C\{^1H\}$ NMR (500 MHz, $CDCl_3$)
O
S
O
(11)
f1 (ppm)
125
124
123
122
121
120
119
118
117
116
115
114
113
112
111
110
109
108
3800
3600
3400
3200
3000
2800
2600
2400
2200
2000
1800
1600
1400
1200
1000
800
600
400
200
0
-200

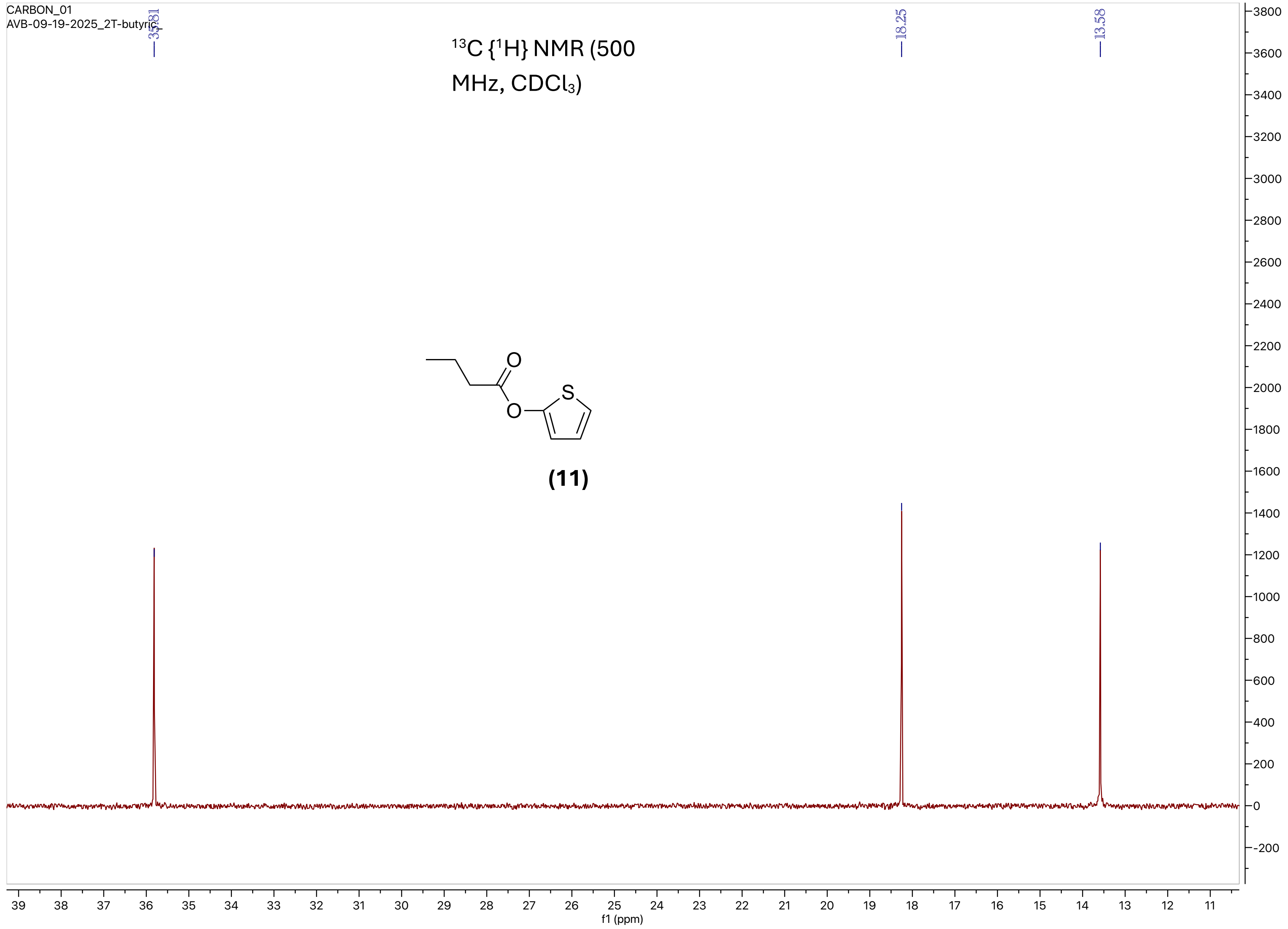

CARBON_01
AVB-09-19-2025_2T-butyric
35.81
18.25
13.58
$^{13}C\{^{1}H\}$ NMR (500 MHz, $CDCl_3$)
O
O
S
(11)
3800
3600
3400
3200
3000
2800
2600
2400
2200
2000
1800
1600
1400
1200
1000
800
600
400
200
0
-200
39 38 37 36 35 34 33 32 31 30 29 28 27 26 25 24 23 22 21 20 19 18 17 16 15 14 13 12 11
f1 (ppm)

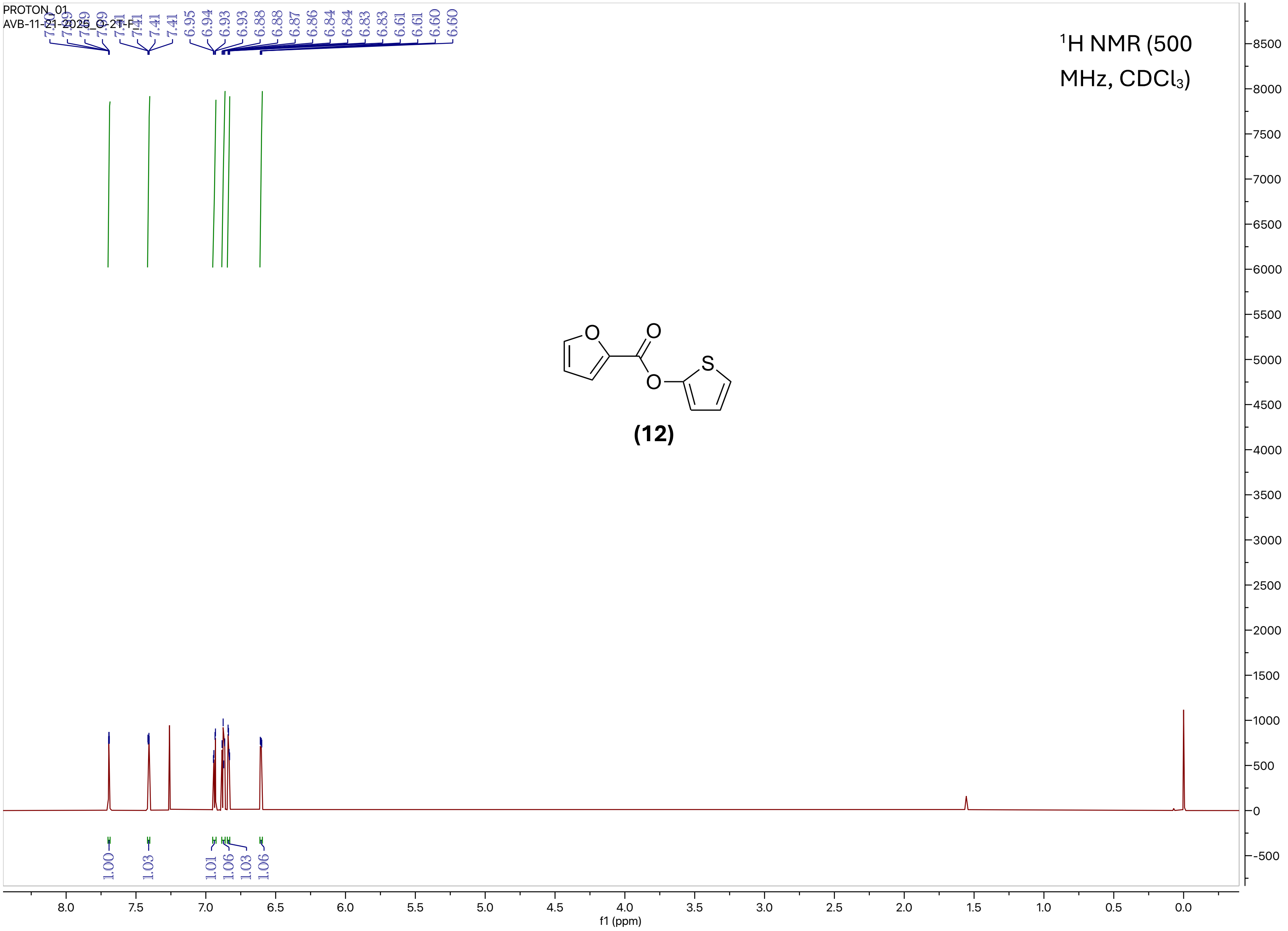
PROTON_01
AVB-11-21-2025_O-2T-F
^{1}H NMR (500 MHz, CDCl3)
(12)
f1 (ppm)

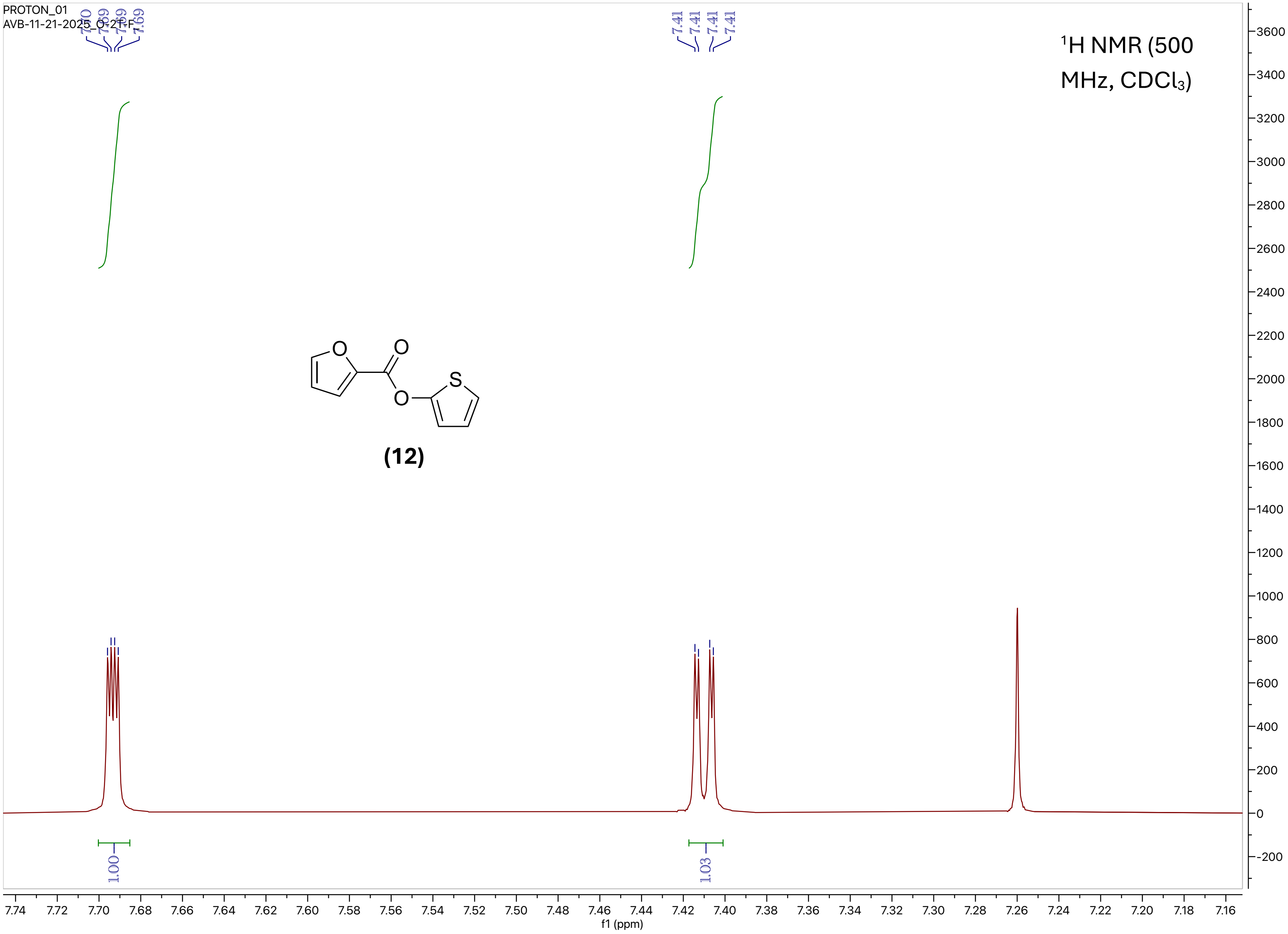
PROTON_01
AVB-11-21-2025_O-2T-F
7.70
7.69
7.69
7.69
7.41
7.41
7.41
7.41
^{1}H NMR (500 MHz, $CDCl_3$)
O
O
O
S
(12)
1.00
1.03
f1 (ppm)

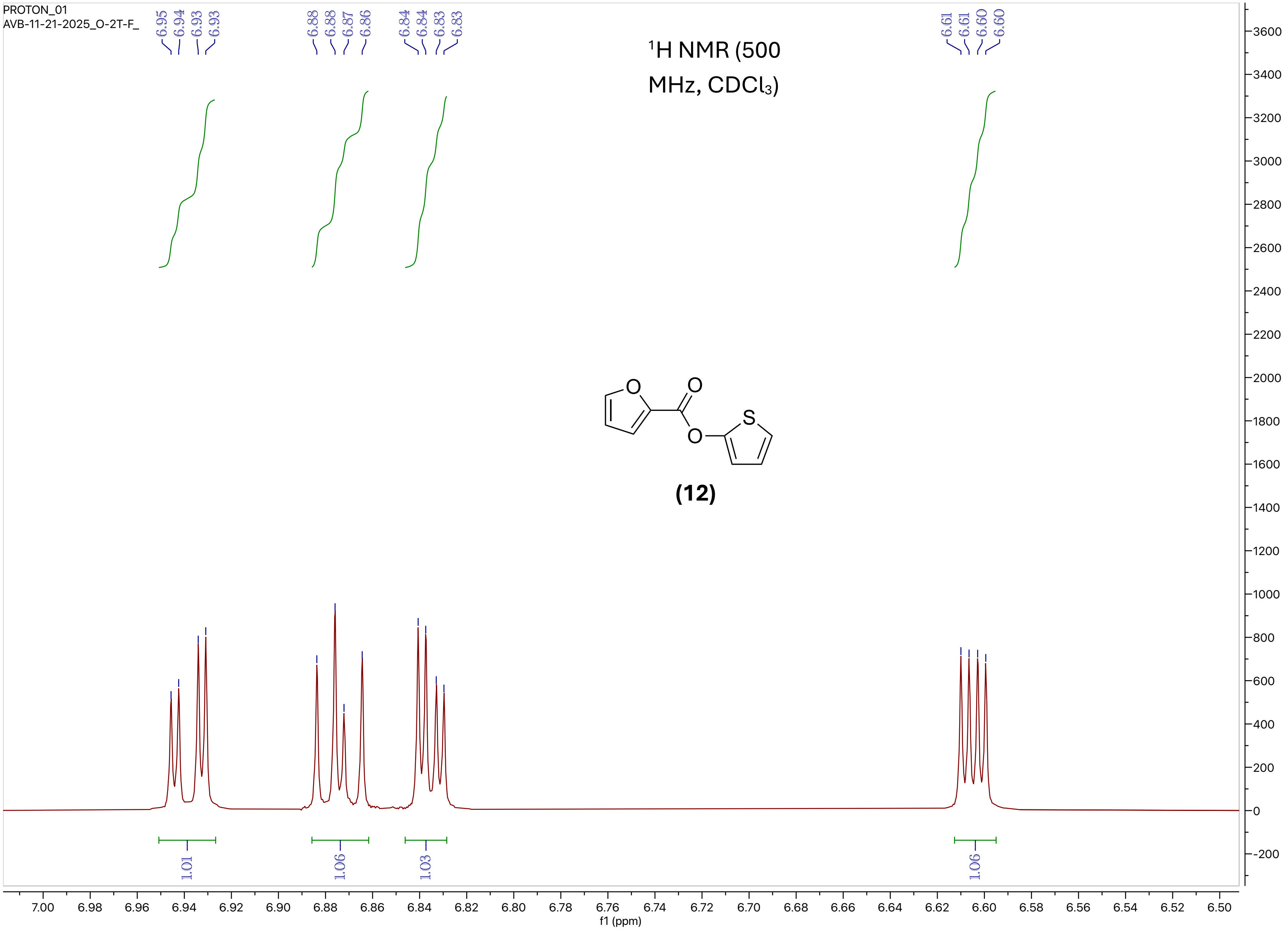
PROTON_01
AVB-11-21-2025_O-2T-F_
6.95
6.94
6.93
6.93
6.88
6.88
6.87
6.86
6.84
6.84
6.83
6.83
6.61
6.61
6.60
6.60
¹H NMR (500 MHz, CDCl₃)
O
O
O
S
(12)
1.01
1.06
1.03
1.06
f1 (ppm)

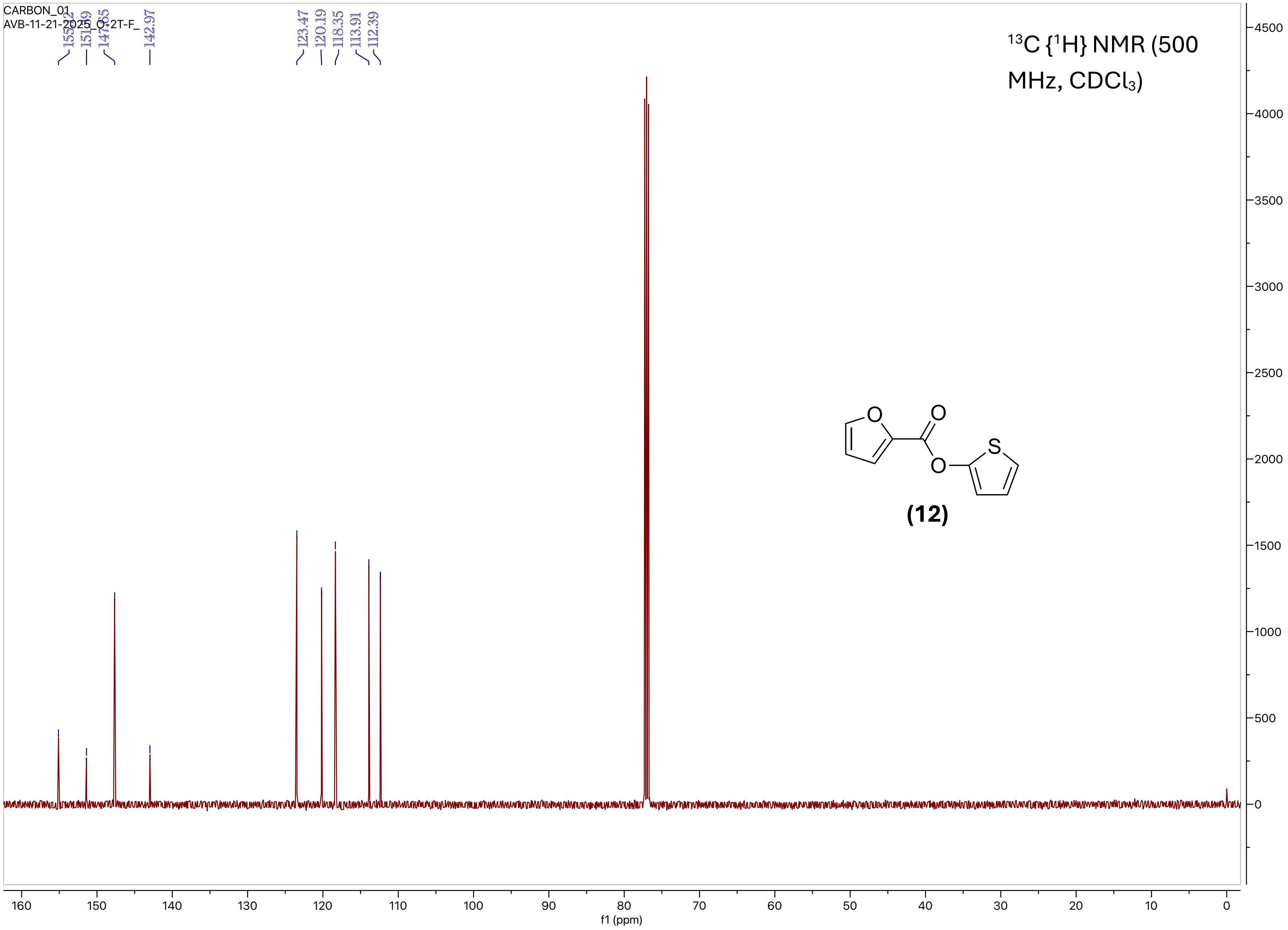

CARBON_01
AVB-11-21-2025_O-2T-F_
142.97
123.47
120.19
118.35
113.91
112.39
$^{13}C\{^{1}H\}$ NMR (500 MHz, $CDCl_3$)
O
O
O
S
(12)
f1 (ppm)

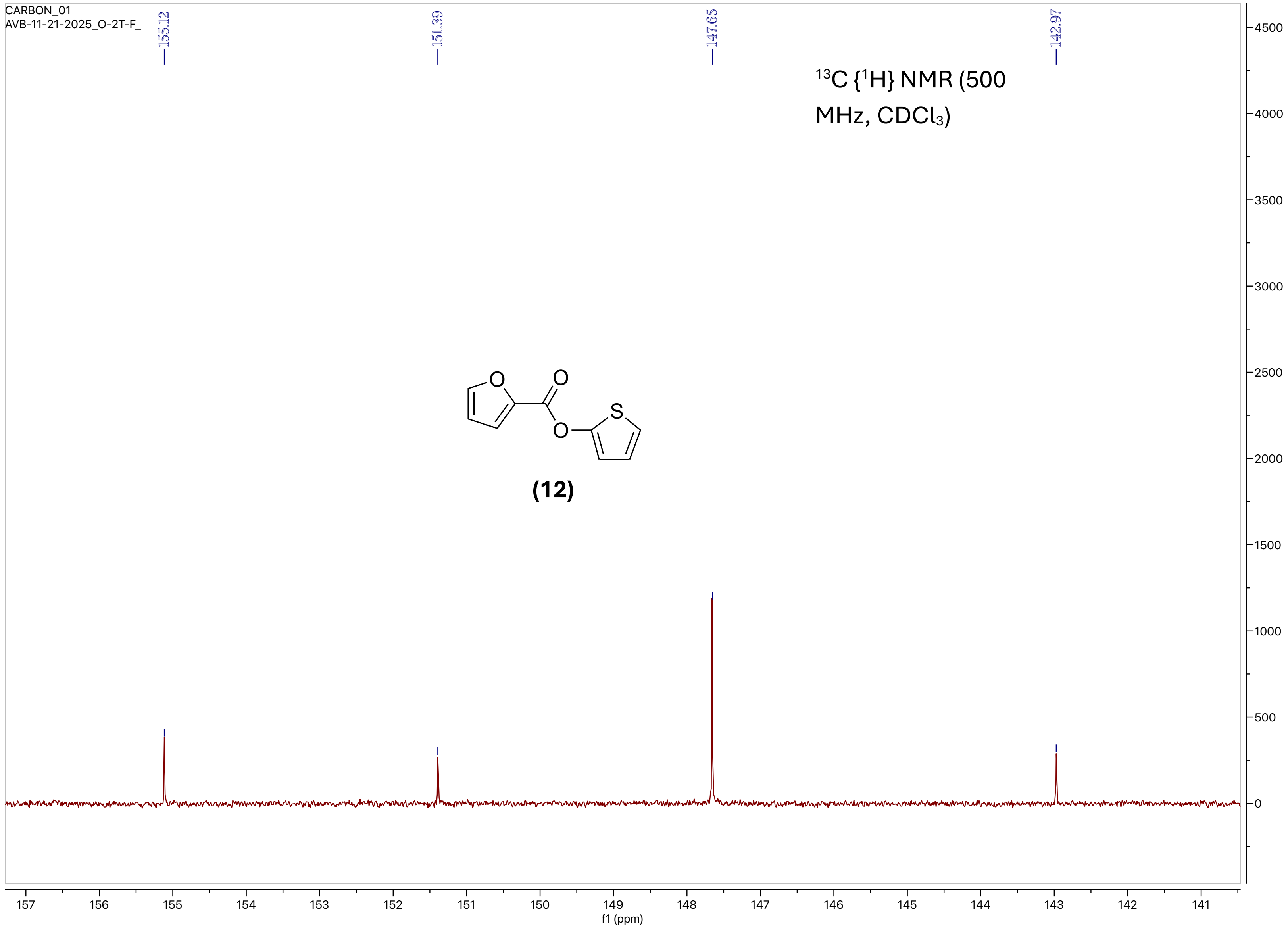
CARBON_01
AVB-11-21-2025_O-2T-F_
155.12
151.39
147.65
142.97
13C {1H} NMR (500 MHz, CDCl3)
O
O
O
S
(12)
4500
4000
3500
3000
2500
2000
1500
1000
500
0
157
156
155
154
153
152
151
150
149
148
147
146
145
144
143
142
141
f1 (ppm)

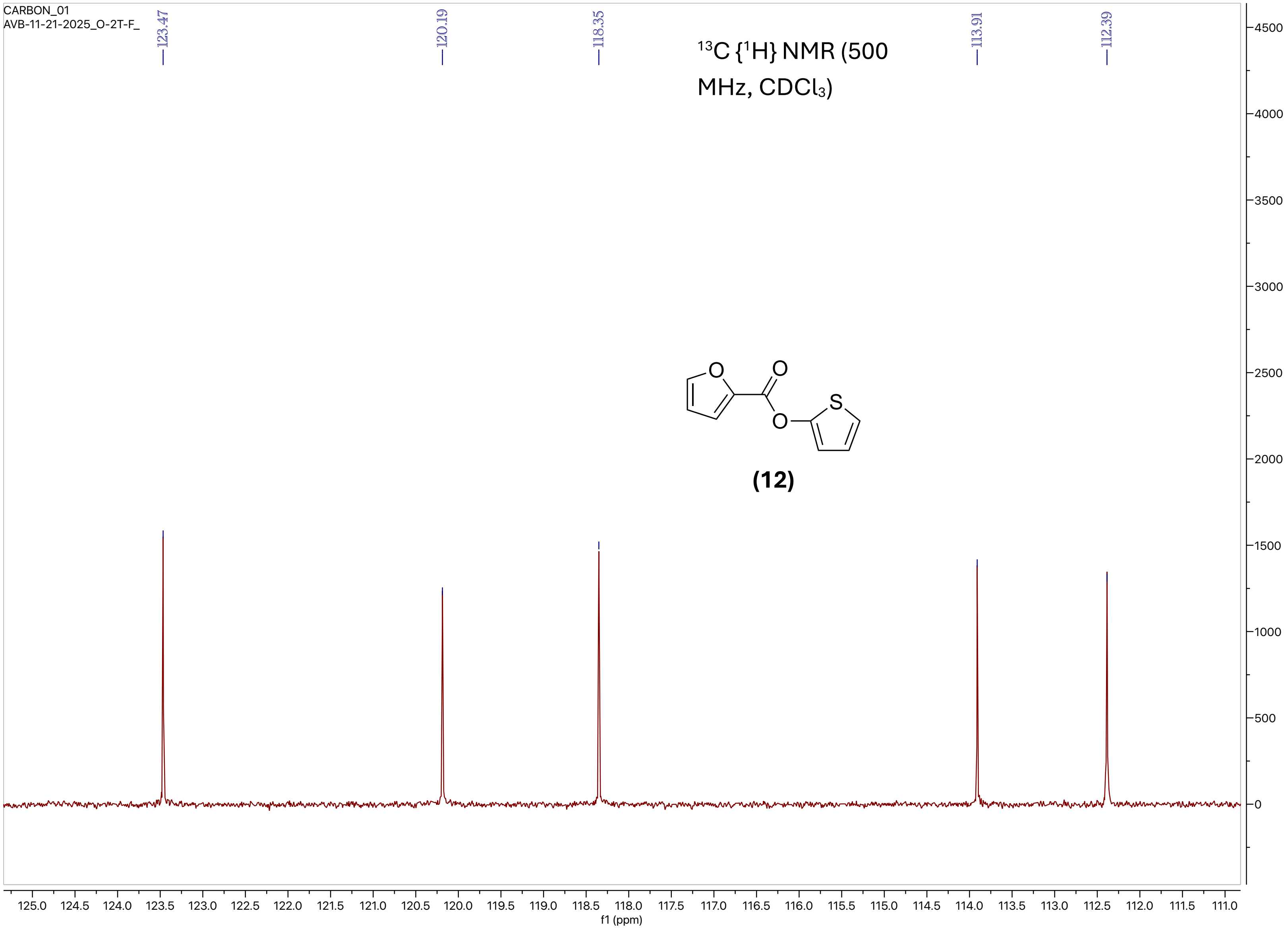
CARBON_01
AVB-11-21-2025_O-2T-F_
123.47
120.19
118.35
113.91
112.39
13C {1H} NMR (500 MHz, CDCl3)
(12)
f1 (ppm)

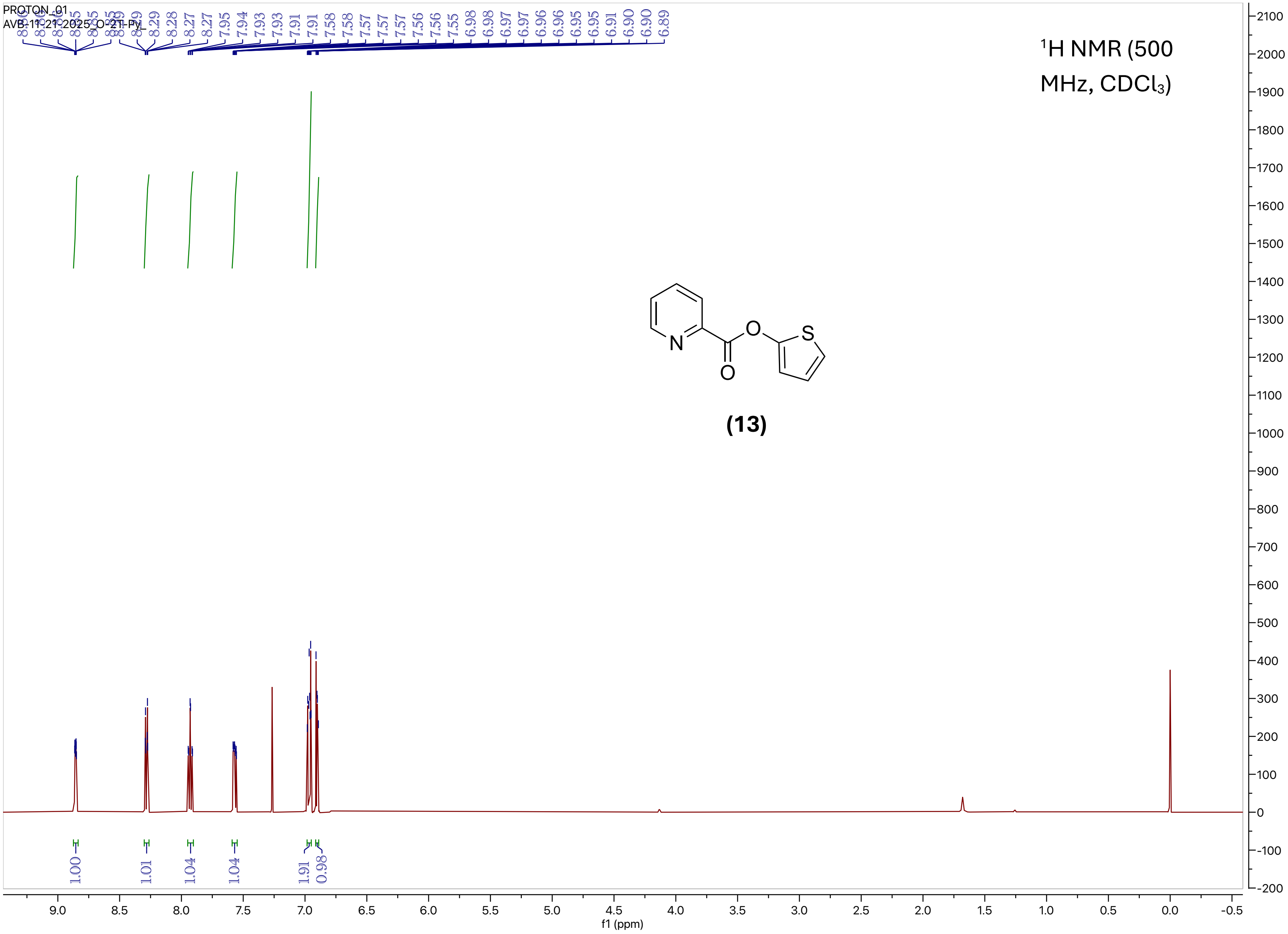
PROTON_01
AVB-11-21-2025_O-2T-Py
¹H NMR (500 MHz, CDCl₃)
N
O
O
S
1.00
1.01
1.04
1.04
1.91
0.98
f1 (ppm)

(13)

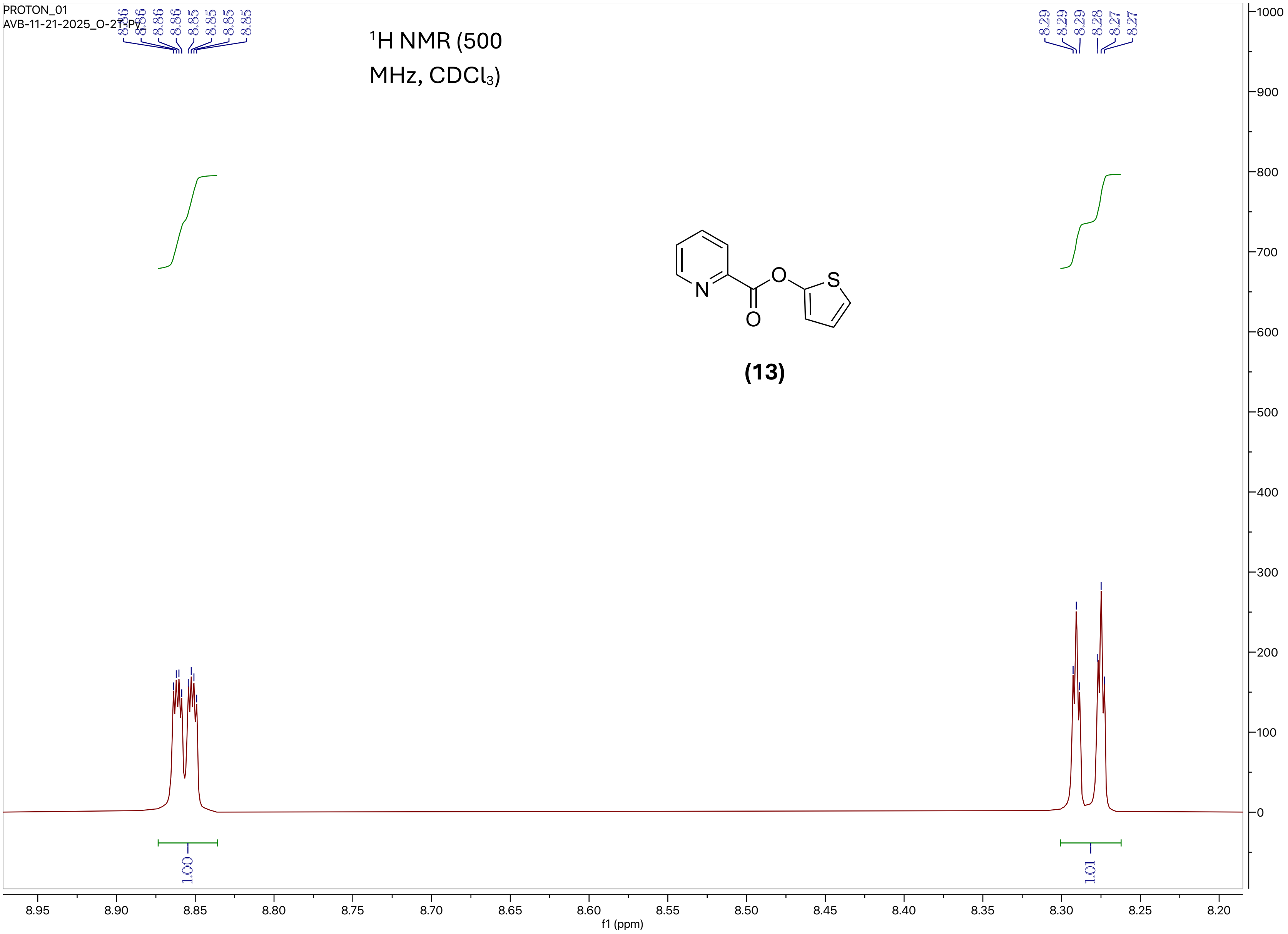
PROTON_01
AVB-11-21-2025_O-2T-Py
^{1}H NMR (500 MHz, $CDCl_3$)
8.86
8.86
8.86
8.86
8.85
8.85
8.85
8.85
8.29
8.29
8.29
8.28
8.27
8.27
1.00
1.01
f1 (ppm)

(13)

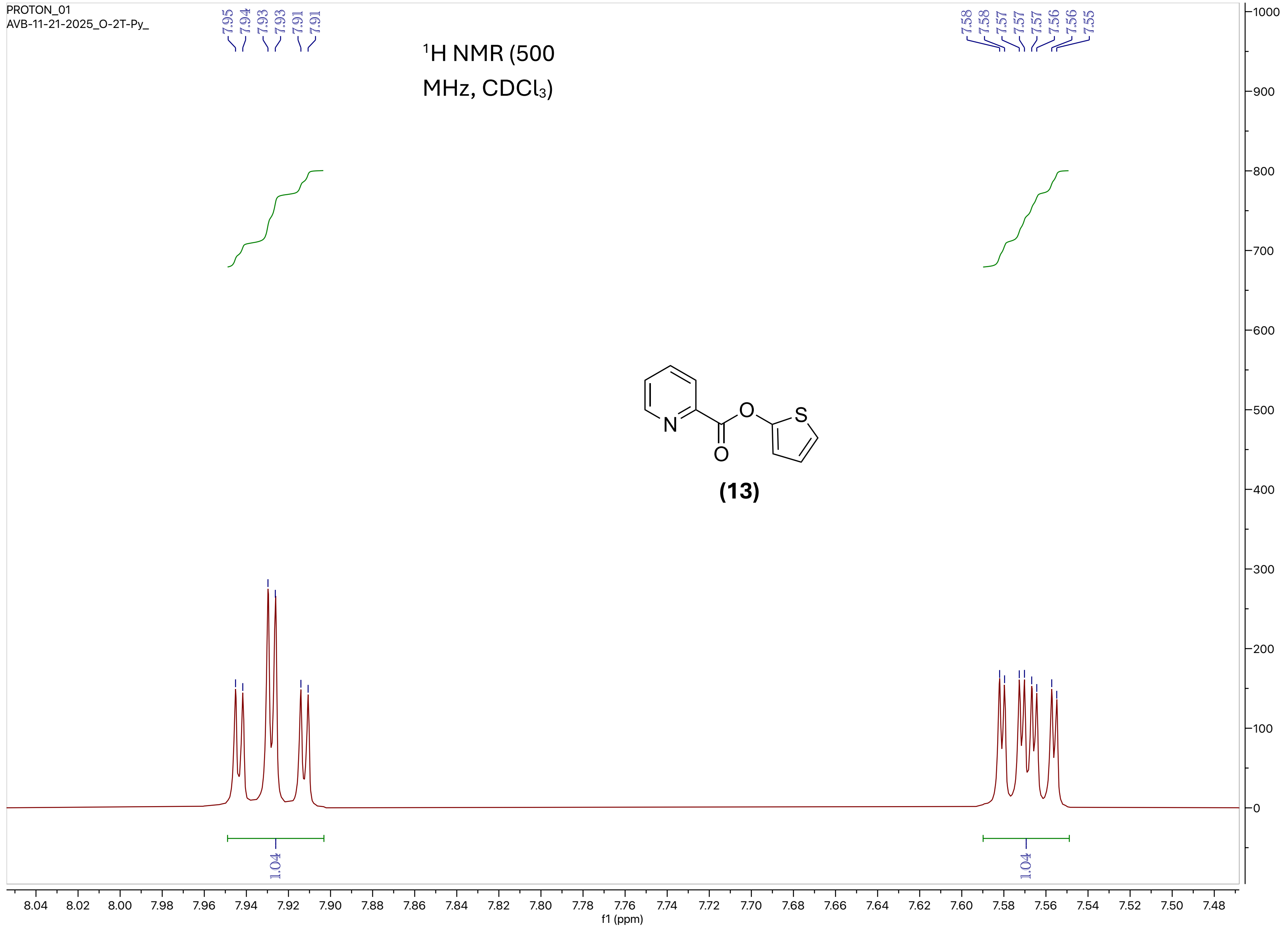
PROTON_01
AVB-11-21-2025_O-2T-Py_
7.95
7.94
7.93
7.93
7.91
7.91
7.58
7.58
7.57
7.57
7.57
7.56
7.56
7.55
1H NMR (500 MHz, CDCl3)
N
O
O
S
1.04
1.04
f1 (ppm)

(13)

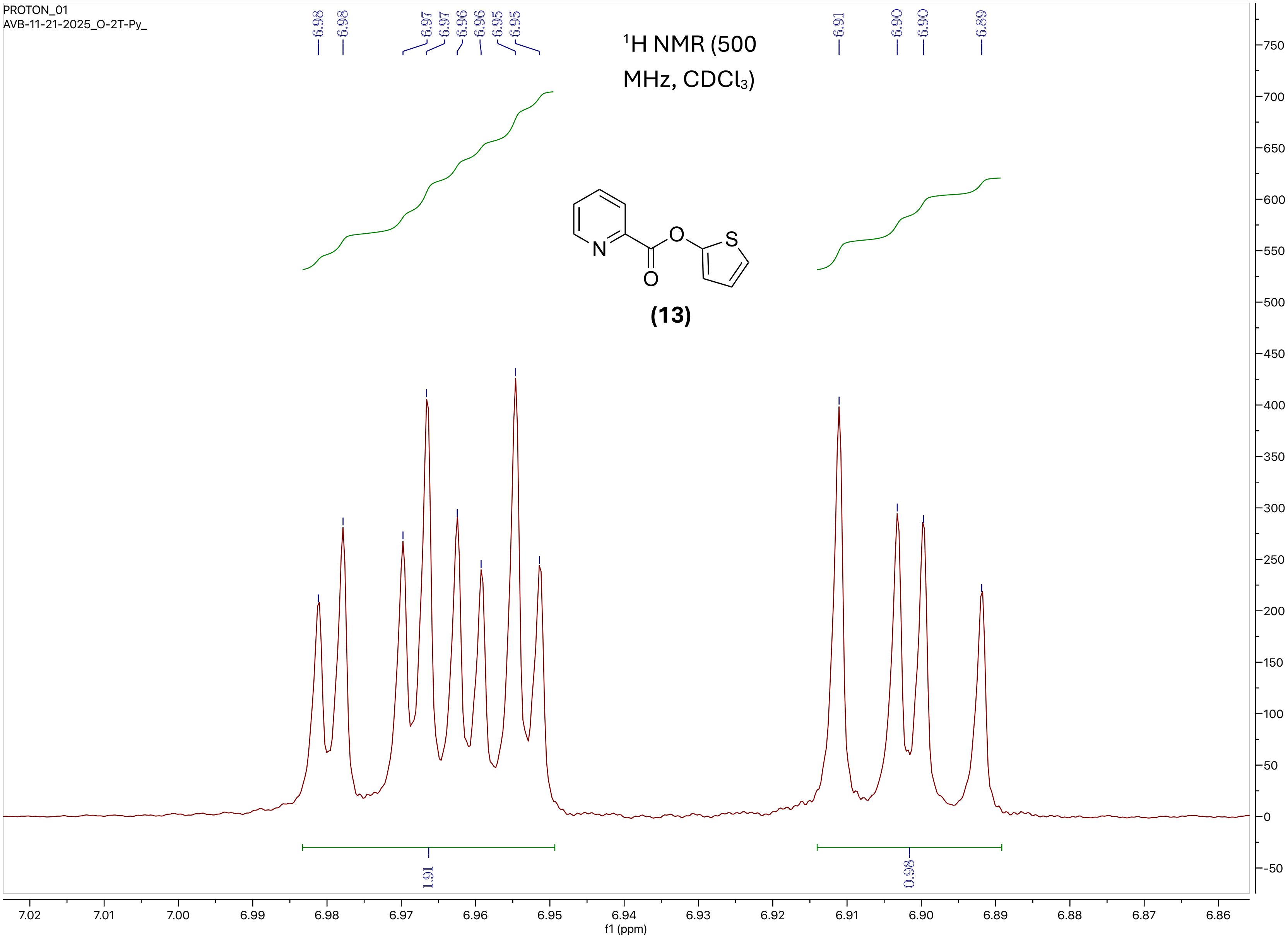
PROTON_01
AVB-11-21-2025_O-2T-Py_
1H NMR (500 MHz, CDCl3)
(13)
6.98
6.98
6.97
6.97
6.96
6.96
6.95
6.95
6.91
6.90
6.90
6.89
1.91
0.98
f1 (ppm)

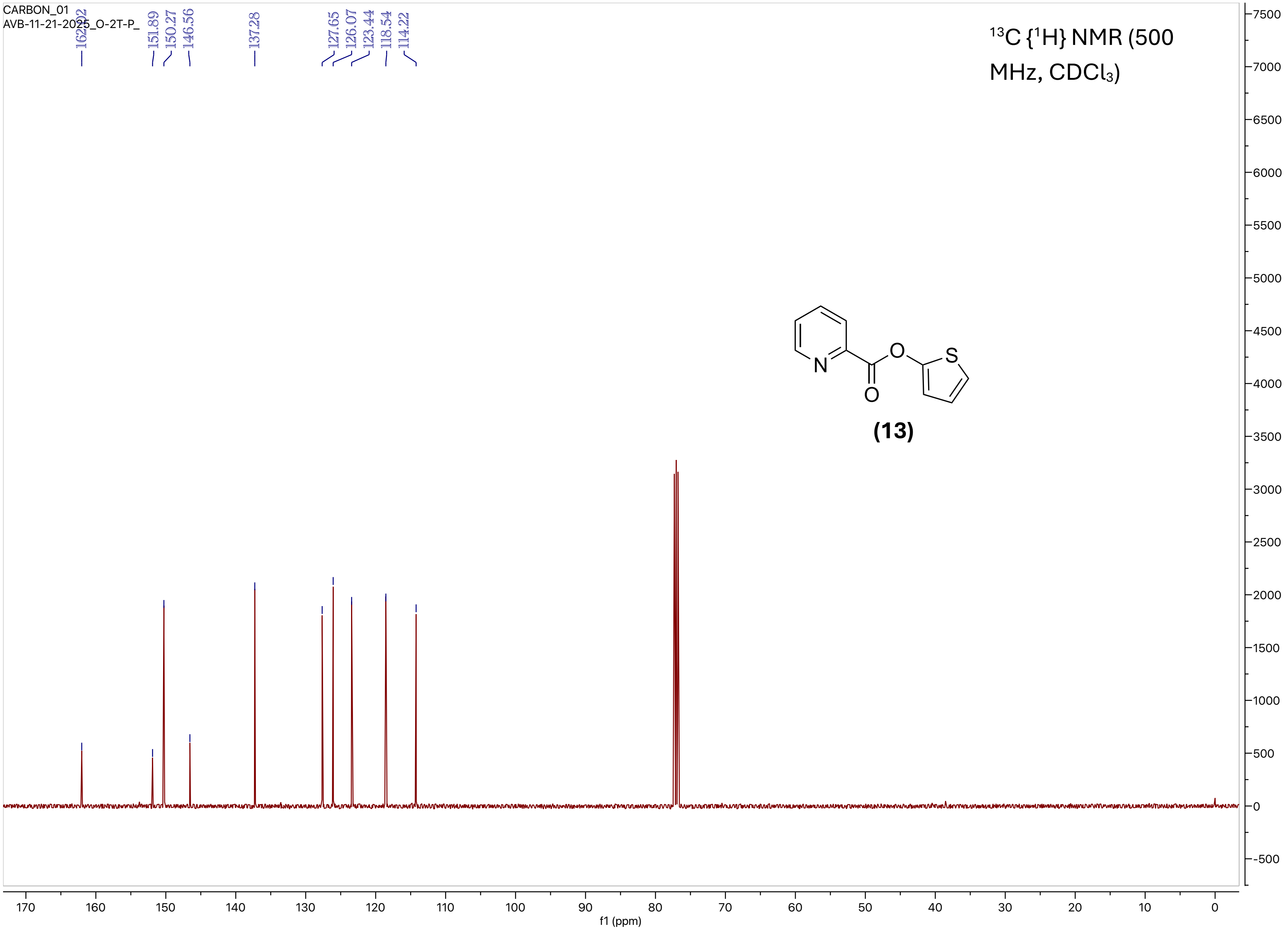
CARBON_01
AVB-11-21-2025_O-2T-P_
162.02
151.89
150.27
146.56
137.28
127.65
126.07
123.44
118.54
114.22
^{13}C {^{1}H} NMR (500 MHz, $CDCl_3$)
N
O
O
S
(13)
f1 (ppm)

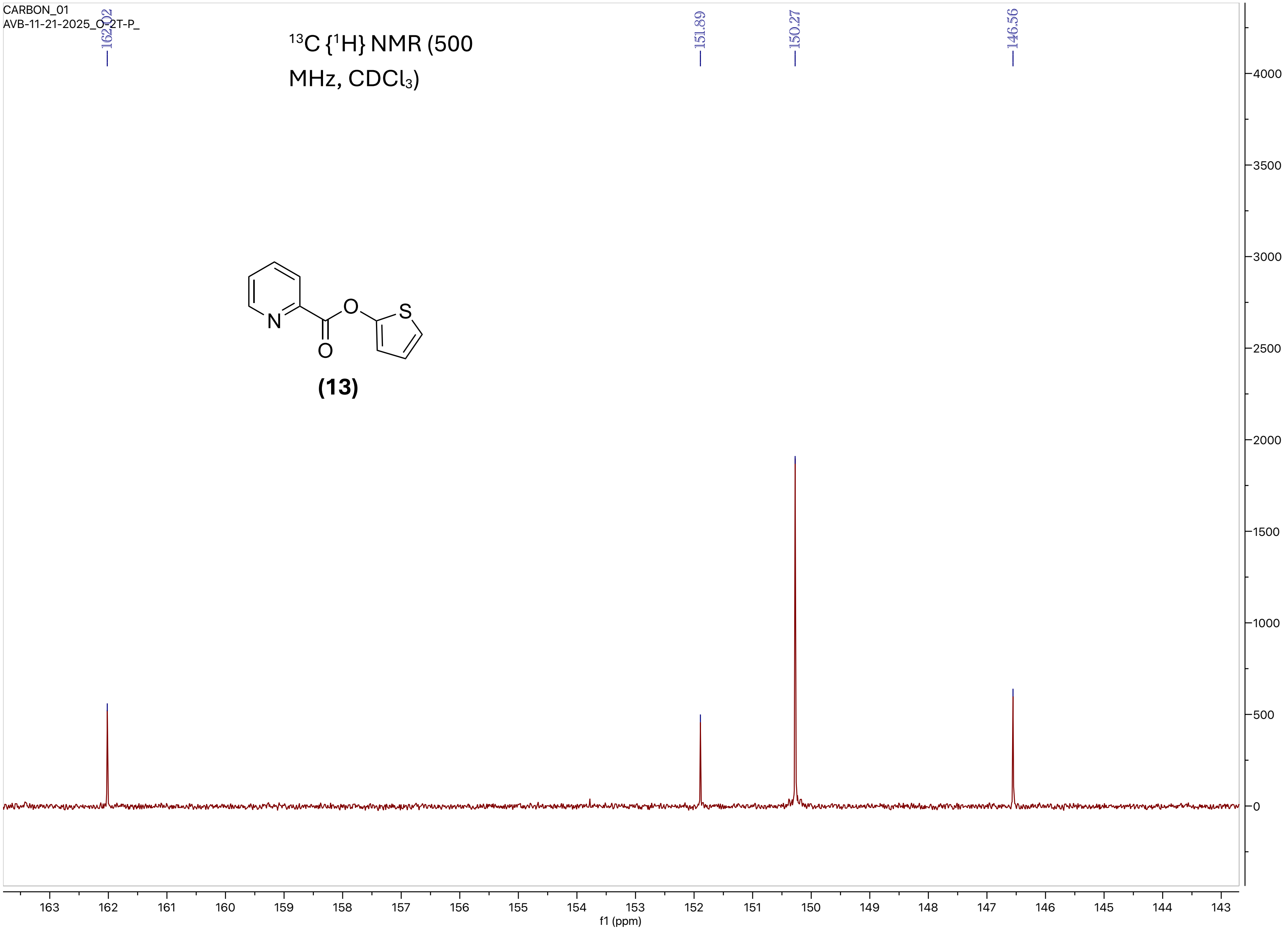
CARBON_01
AVB-11-21-2025_O-2T-P_
162.02
151.89
150.27
146.56
13C {1H} NMR (500 MHz, CDCl3)
N
O
O
S
(13)
f1 (ppm)

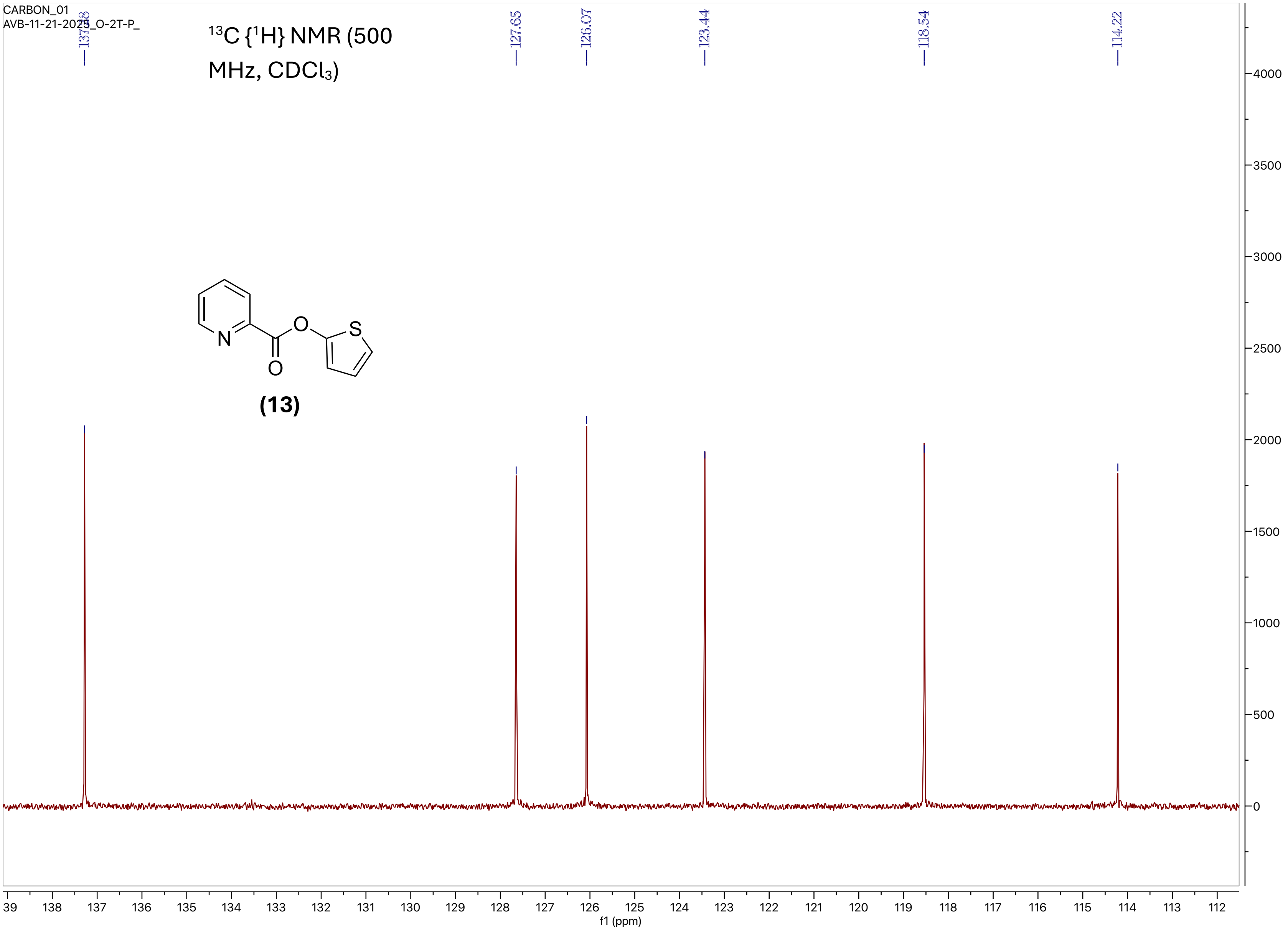
CARBON_01
AVB-11-21-2025_O-2T-P_
13C {1H} NMR (500 MHz, CDCl3)
127.65
126.07
123.44
118.54
114.22
N
O
O
S
(13)
4000
3500
3000
2500
2000
1500
1000
500
0
39 138 137 136 135 134 133 132 131 130 129 128 127 126 125 124 123 122 121 120 119 118 117 116 115 114 113 112
f1 (ppm)
